# Fine particles in homes of predominantly low-income families with children and smokers: Key physical and behavioral determinants to inform indoor-air-quality interventions

**DOI:** 10.1371/journal.pone.0177718

**Published:** 2017-05-17

**Authors:** Neil E. Klepeis, John Bellettiere, Suzanne C. Hughes, Benjamin Nguyen, Vincent Berardi, Sandy Liles, Saori Obayashi, C. Richard Hofstetter, Elaine Blumberg, Melbourne F. Hovell

**Affiliations:** 1 Center for Behavioral Epidemiology and Community Health (C-BEACH), San Diego State University Research Foundation (SDSURF), Graduate School of Public Health, San Diego State University, San Diego, California, United States of America; 2 San Diego State University/University of California, San Diego Joint Doctoral Program in Public Health (Epidemiology), University of California San Diego, La Jolla, California, United States of America; Texas A&M University College Station, UNITED STATES

## Abstract

Children are at risk for adverse health outcomes from occupant-controllable indoor airborne contaminants in their homes. Data are needed to design residential interventions for reducing low-income children's pollutant exposure. Using customized air quality monitors, we continuously measured fine particle counts (0.5 to 2.5 microns) over a week in living areas of predominantly low-income households in San Diego, California, with at least one child (under age 14) and at least one cigarette smoker. We performed retrospective interviews on home characteristics, and particle source and ventilation activities occurring during the week of monitoring. We explored the relationship between weekly mean particle counts and interview responses using graphical visualization and multivariable linear regression (base sample *n* = 262; complete cases *n* = 193). We found associations of higher weekly mean particle counts with reports of indoor smoking of cigarettes or marijuana, as well as with frying food, using candles or incense, and house cleaning. Lower particle levels were associated with larger homes. We did not observe an association between lower mean particle counts and reports of opening windows, using kitchen exhaust fans, or other ventilation activities. Our findings about sources of fine airborne particles and their mitigation can inform future studies that investigate more effective feedback on residential indoor-air-quality and better strategies for reducing occupant exposures.

## Introduction

This article explores physical characteristics of residences and common occupant behaviors (related to air-particle generation and mitigation) that may influence weekly fine airborne particles (0.5 to 2.5 micrometers) in homes of predominantly low-income families containing one or more smokers and at least one young child. Low-income families may be at more risk for particulate matter exposure from smoking and other indoor air contaminants [1,2]. Indoor levels of fine particles in residences are derived from indoor sources or from infiltration of outdoor particles, and can be controlled by occupants [3]. Over 85% of people’s time, on average, is spent indoors and mostly at home [4]. In a seminal review of particles in homes, Wallace et al. (1996) reported smoking and cooking to be important indoor sources [5].

Household occupants can experience short and long-term health effects associated with acute and cumulative exposure to indoor airborne particles. The health consequences are especially important for children whose lungs are not fully developed and who breathe around three times more air per kilogram of body weight than adults [6]. Fine particle pollution in outdoor air is monitored by the U.S. Environmental Protection Agency as part of its PM_2.5_ National Ambient Air Quality Standard to protect public health [7]; particle pollution has been associated with respiratory and cardiovascular effects, and increased morbidity and mortality [8–11]. Particulate matter is also a commonly-used indicator of secondhand tobacco smoke [12]. The acute and chronic detrimental health effects of exposure to tobacco smoke are well-documented [13,14]. Furthermore, the level of fine airborne particles indicates if other combustion-generated pollutants (e.g., volatile organics in cooking emissions or incense emissions) are present and provides information on the effectiveness of pollutant removal mechanisms (e.g., natural or forced ventilation). The level of fine particles in residences depends on emission source activity, the physical characteristics of the residence, and removal activity.

Research is needed on interventions for private homes that promote strategies to avoid or reduce indoor air pollution from tobacco and other sources. By understanding source and mitigation effects for households with different physical characteristics, we can optimize interventions by identifying specific occupant-modifiable behaviors and characteristics. While models [15] and small-scale field studies (e.g., Ott et al. 2003 [16]) have established physics-based theory and the broad impact of different occupant activities on fine particle levels in homes, empirical data from a large sample are needed to confirm influential factors in homes with varying family types.

This study was motivated by a need to develop effective interventions targeting children exposed to secondhand tobacco smoke (SHS) in homes (pilot study described by Klepeis et al. 2013 [17]). As reported by Rosen et al. (2014) in a meta-analysis of 30 child-SHS intervention studies, while some past intervention studies (primarily based on counseling) have shown moderate impact, improved interventions (e.g., with feedback on SHS) are needed to reduce children's SHS exposure [18]. Recently, some intervention researchers have reported on smoke particle levels and exposures in the home, removal rates, alternate sources of fine particles, or the physical dwelling characteristics (e.g., [19,20]), or provide initial evidence that SHS interventions incorporating feedback on SHS levels can reduce particle count measures and presumably child exposures [17,21]. Continued refinement of real-time instruments and their application in real-time interventions may better prevent or reduce SHS exposures and result in more smoke-free environments [17]. Since SHS is the central intervention interest for the present data set and future studies, our investigation focuses initially on levels of particles contributed by smoking, primarily cigarettes. Exposure to third hand smoke (THS), which occurs by direct contact with surfaces or dust contaminated with SHS emissions, or by inhalation of compounds off-gassing from walls, carpets, drapes etc. [22], may also be targeted by interventions.

Few prior air monitoring studies have considered comprehensive source or ventilation-related determinants of particles in homes or measured fine particles in a large sample of low-income homes. A recent study by Urso et al. (2015) of many building factors and their association with size-differentiated particle levels in 60 mostly non-smoking (70%) households in Italy found that sealing doors and windows, using kitchen air-exhaust systems, building density around the home, and larger home volume lowered levels of fine particles [23]. The main determinants of elevated indoor fine particles were tobacco smoking, burning fireplace wood, the number of occupants, and time spent cleaning. Baxter et al. (2007) measured samples of integrated (filter and pump) fine particulate matter in 43 low socioeconomic-status (SES) homes in the Boston Area, confirming that cooking and stove usages, along with resuspension activities (e.g., dusting, sweeping, vacuuming), contribute to indoor levels, with occupant activities driving indoor levels more than housing characteristics [24]. A number of large monitoring studies in Europe, the U.S., and Canada confirm the general importance of tobacco smoking, cooking, cleaning, and outdoor levels as particle determinants [25–31].

The objective of the present study is to provide new findings on fine particle levels in at-risk homes that will better inform efforts to develop and implement particle-reduction interventions, especially for SHS. The unique advantages of the present study are: (1) its focus on predominantly low-income household residents, who are likely to be at significant risk to smoking and other indoor contaminants; (2) a relatively large analytic sample size (*n* = 262); (3) interview data for many different types of smoking and other particle-generating activities, ventilation, and home size; and (4) week-long continuous monitoring data, which cover weekday and weekend activity cycles. These features of our study allow us to examine a wide range of potential fine particle sources (e.g., tobacco, marijuana, incense, e-cigarettes), home sizes, potential behaviors (e.g., fan use, indoor/outdoor smoking) in predominantly low-income homes that may affect air quality and may be incorporated into feedback provided to intervention participants.

## Materials and methods

### Data source

Data for this study were from participants enrolled in a randomized controlled intervention trial designed to reduce secondhand tobacco smoke (SHS) exposure in children by making use of real-time feedback on particle levels (ClinicalTrials.gov Identifier NCT01634334). The present paper is limited to investigation of particle levels and their determinants in participants’ homes during a baseline period for combined treatment and control groups. Future papers will examine the overall outcome of the intervention trial and the impact of particle feedback, including differential levels by study condition. This study enrolled 298 households (one adult and one child participant per household), who were recruited during 2012–2015 from multiple sources, including Women, Infants, and Children (WIC) Special Supplemental Nutrition Programs sites in San Diego Country, community tabling events, health care professional referrals, local organizations, and advertisement. Eligibility criteria included: parent/guardian 18 years or older; at least one child under 14 years of age in the household; at least one smoker living in the household who smoked inside or outside the home; no plans to move outside the County within 4 months; and speaks English or Spanish. The San Diego State University Institutional Review Board approved the procedures for this study (IRB Study Number 770080). One adult participant in each household signed an informed consent agreement, whereupon the adult and the youngest child in the home were enrolled as participants in the study. The first 36 homes enrolled comprised our pilot study sample and participants in these homes were not administered the final smoking-related questions; the pilot homes were excluded from our analysis, leaving an analytic base sample of 262 households.

The participant characteristics for the analytic sample are summarized in Table 1. Enrolled parents (EP) were 95% female, 38% Hispanic, 27% Non-Hispanic White, and 67% with ages between 25 and 40; 40% were employed; and 36% had no education beyond high school. Enrolled children (EC) were 48% female, 49% Hispanic, 19% Non-Hispanic White, and averaged 4.2 (SD = 3.7) years of age. The median categorical annual family income was $20,000 to $30,000 with 39% of families having income less than $20,000 and 81% of families having income less than $50,000. For reference, according to the US Censor Bureau (2015), the poverty thresholds for households with 3 to 6 people and 1 to 5 children are roughly in the range of $20,000 to $30,000 per year [32].

**Table 1:** Characteristics for enrolled child (EC) and enrolled parent (EP) participants of analytic household sample (*n* = 262).

Characteristic	*n*	(%) ^b^
Anyone smoked tobacco or e-cigs in home, past 7 days	91	(34.6%)
Number of children in the household		
1	94	(35.9%)
2	82	(31.3%)
3	50	(19.1%)
4	22	(8.4%)
5 or more	14	(5.3%)
Number of adults in the household		
1	18	(6.9%)
2	127	(48.5%)
3	64	(24.4%)
4	32	(12.2%)
5 or more	21	(8.0%)
EP is female	250	(95.4%)
EP age, years		
18 to 24.99	44	(16.8%)
25 to 29.99	67	(25.6%)
30 to 39.99	109	(41.6%)
40 to 62	42	(16.0%)
EP race / ethnicity		
Hispanic	100	(38.2%)
Non-Hispanic Black	39	(14.9%)
Non-Hispanic White	71	(27.1%)
Non-Hispanic Other ^a^	52	(19.8%)
EP education, years completed		
<12 years	44	(16.9%)
12 years	49	(18.8%)
>12 years	168	(64.2%)
EP is a single parent	97	(37.0%)
EP is the biological mother of EC	222	(84.7%)
EP is employed	104	(39.7%)
Family Annual Income		
less than $10,000	56	(21.3%)
$10,000–$19,999	46	(17.4%)
$20,000–$29,999	47	(17.9%)
$30,000–$39,999	37	(14.0%)
$40,000–$49,999	27	(10.2%)
$50,000–$59,999	16	(6.0%)
$60,000–$69,999	10	(3.8%)
$70,000–$79,999	9	(3.4%)
$80,000 or more	16	(6.0%)
EC is female	125	(47.7%)
EC age, years		
0 to 2	101	(38.5%)
2 to 6	91	(34.7%)
6 to 14	70	(26.7%)
EC race / ethnicity		
Hispanic	129	(49.2%)
Non-Hispanic Black	32	(12.2%)
Non-Hispanic White	49	(18.7%)
Non-Hispanic Other ^a^	52	(19.8%)

^a^ "Non-Hispanic Other" includes: Native American, Asian, Pacific Islander, mixed, unspecified race.

^b^ The denominator in the calculation of percentages is the total number of households, n = 262.

### Data collection and measures of interest

This article focuses on the first week of baseline data collection, which spanned the time between home Visit 1 and Visit 2, roughly 7 days apart. We collected data on the physical characteristics of the residence, emission source activity, ventilation activity, and continuous particle counts. During Visit 1, trained research assistants used Bosch Laser Distance Measurers (DLR130K, Robert Bosch GmbH, Stuttgart, Germany) to measure the length, width, and height of the room containing the monitor from which we calculated the *room volume*. The physical characteristics (Table 2) were obtained from interviews and extracted from detailed floor plans drawn by trained staff.

**Table 2:** Study variables for home characteristics and occupant behaviors.

Variables	Measurement Method
*Distance from roadway*	(via direct on-site observation) We estimated the distance from the road to the nearest window in three categories: <50 feet; 50–100 feet; >100 feet.
*Home type*	(via direct on-site observation) We categorized homes into: (a) detached house, (b) townhouse, (c) apartment/condo, (d) duplex, or (e) trailer/mobile home. A small number of townhouses, duplexes, and mobile homes were combined into a separate category labeled “other”.
*Number of levels*	(via interview) The number of levels in each home.
*Number of rooms*	(via interview) The total number of rooms in each home.
*Number of bedrooms*	(via interview) The number of bedrooms in each home.
*Number of bathrooms*	(via interview) The number of bathrooms in each home.
*Number of exterior doors*	(via interview) The number of doors leading to a patio or balcony; due to the skewed distribution (6 homes reported between 5 and 9 exterior doors), responses to this question were truncated to 4 doors for analysis.
*Occupant Particle Source Activity—Smoking*	(via interview) For cigarettes, cigars, pipe tobacco, hookah/waterpipe, electronic cigarettes, medicinal or recreational marijuana, other recreational drugs, participants were asked “How often in the past 7 days did anyone smoke _________ in your home?” Responses were coded: never, 1–3 times, 4–6 times, 7–9 times, ≥10 times and dichotomized in the analysis to “0 times” and “> 0 times”.
*Occupant Particle Source Activity—Other than Smoking*	(via interview) Participants were asked the number of days in the past 7 days that each of the following occurred: use of wood burning stove or fireplace, use of gas heater, burning incense or candles, burning food, frying or sautéing food with oil or fat, use of gas/propane appliance to cook or heat food, use of electric appliance to cook/heat food, use of aerosol spray products, and vacuuming-dusting-sweeping. Responses were dichotomized in the analysis to “0 days” and “> 0 days”.
*Occupant Ventilation Activity*	(via interview) Participants were asked the number of days in the past 7 days that each of the following occurred: use of central air handling system, use of an air purifier, use of an exhaust fan in the kitchen, use of a window fan or window air conditioner, opened a window, and opened an exterior door. Responses were dichotomized in the analysis to “0 days” and “> 0 days”.

At Visit 1, a customized Dylos™ DC1700 Air Quality Monitor (Dylos Corporation, Riverside, CA USA) was placed in homes to continuously measure counts per 0.01 cubic feet of particles with diameters between 0.5 and 2.5 micrometers. As described by Klepeis et al. 2013 [17] for a similar instrument used in an SHS intervention, the Dylos unit had expanded data storage, special firmware, wireless connectivity, and lights and sounds triggered by particle level thresholds. Lights and sounds [33] were disabled for the baseline data used in the present analysis. The monitors recorded 10-second average counts (derived from 1-second average readings in internal memory) in the room closest to the usual cigarette smoking location (as reported by the participants). Monitors were placed at least 3 feet from the floor and a minimum of 1 foot from the nearest wall to provide consistent air flow. The feasibility of using Dylos-brand monitors to measure fine particle levels in homes or under controlled conditions has been demonstrated [17,34–40]. For fresh emissions, Klepeis et al. (2013) and Dacunto et al. (2015) reported reasonable agreement between the Dylos and mass particle concentrations of tobacco smoke measured every minute using calibrated industry-standard particle-mass-measurement instruments (DustTrak and SidePak, TSI, Inc., Shoreview, MN) [17,40]. Semple et al. (2013) showed a trend for long-term SidePak versus Dylos measurements in 34 homes [39]. In general, the Dylos factory-calibrated monitors provide acceptable unit-to-unit consistency and, while their accuracy for measuring absolute fine particle mass concentrations for different sources is low, they are still useful for providing broad feedback in behavioral applications or personal awareness activities, and for investigating relative measures of particle counts within and between homes, as in the current application.

During Visit 2, trained staff administered a computer-assisted face-to-face interview to gather data about behaviors during the preceding 7-day period. Key questions were asked about the smoking habits of each household member, other particle-generating behaviors (e.g., burning candles, using a wood fireplace), and ventilation activities (Table 2). Participants were provided between $20 and $35 (modified during the study with IRB approval) for participating in the interview.

For data analyses, responses to questions about particle generating behaviors and ventilation activities were dichotomized—effectively measuring whether the activity occurred on *any* day during the last 7 days (“0 days” versus “> 0 days”; or “0 times” versus “> 0 times” for smoking behaviors only). This procedure was used to facilitate interpretation of regression coefficients for variables originally measured using different scales (Table 2).

The dependent variable used for analysis in this study was *weekly mean particle levels*. For each home, all available particle data from the week-long period prior to the baseline interview (e.g., from Monday to Monday) were averaged. All homes included in the final analytic sample for regression modeling provided data for least 5 days; there was one home that contributed only 5 days of data, 3 homes that contributed 6 days of data, 145 that contributed 7 days of data, and 44 homes that contributed 8 days of data (due to delays in scheduling Visit 2).

### Data analysis

We used conventional techniques in our analysis approach to provide an overview of the data to identify influential correlates, and provide both graphical (model-free) and model-based indicators of association. Our approach consisted of three components: (1) descriptive statistics of interview variables for indoor versus no reported indoor smoking; (2) graphical visualization of weekly mean particle count distributions as a function of interview variables; and (3) a statistical model-building approach employing linear regression to identify influential variables. We accomplished all graphical and statistical analysis using the R programming environment [41].

Of the 298 households enrolled in the larger trial, the first 36 pilot homes enrolled were not asked questions about key particle generating behaviors related to smoking, leaving 262 (88%) homes eligible for analysis. Graphical analyses were conducted using all available data. See S1 and S2 Tables with sample sizes for graphical analysis subgroups. To ensure that each stage of statistical model building was conducted on the same sample, we used a complete-case analysis, whereby homes were included that provided data for all potential correlates that were significantly related to mean 7-day particle levels at an alpha level of 0.20 (from bivariate analysis of variables shown in Table 2). This procedure resulted in a final modeling sample of 193 homes.

#### Descriptive statistics of all potential correlates

The potential correlates are housing characteristics, and particle-generating and ventilation activities. We report descriptive statistics for the overall sample, as well as homes with and without self-reported indoor cigarettes smoked, providing an analysis of the composition of two sub-groups of primary interest with substantial sample size. Assessment of the contribution of indoor cigarette smoking to indoor particles and examination of potential confounding variables provide a foundation for subsequent analysis and the evaluation of particle sources in future interventions. Means and standard deviations were used to summarize continuous variables and percentages for categorical variables. We used two-sample t-tests assuming equal variance for continuous variables and chi-square tests for variables with > 2 categories. Binary variables were compared with two-sided tests for equal proportions. We used Fisher’s Exact test for comparisons of variables with an *n*-size < 5 in any cell.

#### Graphical analysis

The visualization of empirical data using graphs provides a means to: (1) understand broad, raw effects of different variables unclouded by parametric assumptions; (2) interpret data in light of expected results (from particle dynamics and the mass balance equation; see [42,43]); (3) inform variable selection in our subsequent parametric, multivariable (regression) analysis; and (4) corroborate the findings of parametric analysis. Graphs are an established feature of exploratory data analysis [44]. We use log-probability plots, a type of quantile-quantile plot, to provide non-parametric visualization of empirical cumulative particle concentration distributions. In general, a quantile-quantile plot, such as those implemented with the qqnorm() function in R [41], consists of the sorted values of a numeric vector plotted against its computed normal quantiles (i.e., quantiles if the distribution is normal). For the special case of a log-probability plot, we use a logarithmic vertical axis and normal-quantile horizontal axis labeled with probabilities rather than quantile values, so that data appearing along a straight line are log-normally distributed and one can easily read the approximate percentiles for each plotted distribution. This type of plot facilitates visual analysis and comparison of distributions for approximately log-normal responses. The plots reveal the sample size along the whole distribution, highlighting areas where a small sample size may mask comparative effects.

#### Statistical model building

The weekly mean particle counts were log-transformed using natural logarithms to better meet the ordinary least squares (OLS) assumption of normality, though the distribution remained slightly right-skewed. Log-transforming the dependent variable (weekly mean particle counts) had the added benefit of improving interpretability of the regression coefficients (β) such that a 1-unit change in any continuous variable or an overall change in any binary independent variable was associated with an (e^β^-1)*100 percentage change in the dependent variable (holding all other correlates constant) [45]. Selection of influential correlates was accomplished using a common stepwise model-building procedure that can be used for traditional hypothesis testing, but is more appropriate for exploratory studies such as the present study [46,47]. The 3 steps in the procedure are listed below. Stepwise selection can eliminate variables that influence effect estimates but do not improve overall model fit; to protect against this, we included a model-building step to identify confounders (Step 3).

1. The weekly mean particle counts were regressed on each potential correlate (shown in Table 2) using single-variable linear regression models (i.e., bivariate, or simple linear regression). Particle-generating and ventilation activities reported by 10 homes or fewer were not considered in our analysis due to insufficient sample size. Marginally-significant correlates (p-value of ≤ 0.20) were retained for further variable selection procedures [48,49].
2. Three multivariable linear regression models were fit using potential correlates retained from Step 1; one containing household characteristics, one containing particle-generating behaviors, and one containing ventilation activities. Multicollinearity was assessed in all models using the variance inflation factor (VIF) with a VIF ≥ 5 indicating multicollinearity; potential correlates in all three models had VIFs < 2. The OLS assumption of linearity was confirmed by two-way plots of the final model residuals and each independent variable. To identify influential correlates, we used stepwise selection with “backward elimination” then “forward selection” based on the Akaike Information Criterion (AIC) using the *step()* function in R [41], separately for each of the 3 multivariable models: household characteristics, particle generating behaviors, and ventilation activities.
3. Correlates identified using selection procedures from all three models in Step 2 formed a final multivariable linear regression model. To isolate additional influential variables (confounders), each potential correlate removed during Step 2 was reentered into the final model one at a time beginning with the variable with the smallest p-value from Step 1. Variables were retained in the final model only if their inclusion resulted in a ≥ 10% change in the beta coefficient of statistically significant variables in that model. White’s method of adjusting for heteroscedasticity [50,51] was employed using the Heteroskedasticity Consistent Covariance Matrix Estimation function (*vcovHC*) within the *sandwich* R package [41]. We report unstandardized beta coefficients.

#### Additional tests

We tested whether the association of weekly mean particle counts and ventilation activities differed for homes with and without indoor cigarette smoking by including multiplicative interactions (e.g., any reported indoor cigarette smoking * open a window) into the final model and evaluating the coefficients of the interaction terms. Coefficients with a p-value < 0.10 were reported. We also tested for potential selection bias by comparing all potential correlates between the set of 193 homes with complete data and the set of 69 homes omitted from this section of the analysis due to missing data for at least 1 potential correlate.

## Results

### Descriptive statistics of housing characteristics, particle-generating and ventilation activities

Table 3 shows the 193 homes included in the statistical analysis split by indoor cigarette-smoking status, which is a key variable of interest. Overall, they had an average of 2.6 bedrooms, 1.6 bathrooms and were mostly one story (mean (SD) number of levels = 1.1 (0.4)). In total, 44 (22.8%) homes reported at least one household member smoking at least one cigarette indoors in the 7-days prior to interview. With the exception of the number of doors leading outside, none of the home characteristics of families with and without self-reported indoor cigarette smoking differed significantly (p’s ≥ 0.05). Homes without indoor cigarette smoking reported indoor smoking of cigars (1.3%), hookah (0.8%), electronic cigarettes (14.1%), marijuana (10.1%), and other drugs (0.7%). With the exceptions of cigar, pipe, electronic cigarette, and marijuana smoking, the 7-day prevalence of all particle generating behaviors did not differ significantly by indoor cigarette smoking status (p’s ≥ 0.05). Nearly all homes reported opening a window (95.3%) or opening a door (96.9%) for ventilation purposes. Most homes (60.1%) reported using an exhaust fan in their kitchen and 8.3% of homes used an air purifier. There were no significant differences between homes with and without indoor cigarette smoking with respect to ventilation activities (p’s ≥ 0.05), except the use of central air conditioning which was higher among homes without indoor cigarette smoking (25.5% vs. 6.8%; p = 0.030).

**Table 3:** Summary statistics for housing characteristics, and particle-generating and ventilation activities compared by indoor cigarette smoking status.

	Overall		Any indoor cigarette smoking		No indoor cigarette smoking		
	mean or n	(SD) or (%)	mean or n	(SD) or (%)	mean or n	(SD) or (%)	p
TOTAL	193		44		149		
**Home characteristics**							
Room Volume (ft^3^)	2018	(1,214)	1986	(1171)	2027	(1230)	0.841^‡^
Number of levels	1.1	(0.4)	1.2	(0.4)	1.1	(0.3)	0.406^‡^
Number of rooms	6.2	-2.4	6.6	(2.6)	6.1	(2.3)	0.309^‡^
Number of doors leading outside	2.0	(1.1)	1.7	(1.2)	2.1	(1.0)	**0.048**^‡^
Number of bedrooms	2.6	(1.0)	2.8	(1.2)	2.5	(1.0)	0.138^‡^
Number of bathrooms	1.6	(0.6)	1.8	(0.8)	1.6	(0.6)	0.054^‡^
Distance from Roadway							0.423^¶^
Roadway <50 feet	112	(58.0%)	22	(50.0%)	90	(60.4%)	
Roadway 50–100 feet	41	(21.2%)	12	(27.3%)	29	(19.5%)	
Roadway >100 feet	40	(20.7%)	10	(22.7%)	30	(20.1%)	
Home Type							0.334^¶^
Condo/Apt.	87	(45.1%)	24	(54.5%)	63	(42.3%)	
Detached house	83	(43.0%)	15	(34.1%)	68	(45.6%)	
Other	23	(11.9%)	5	(11.4%)	18	(12.1%)	
**Indoor particle generating activities**							
Cigarette smoking	44	(22.8%)	44	(100%)			
Cigar smoking	10	(5.2%)	8	(18.2%)	2	(1.3%)	N/A
Pipe tobacco smoking	3	(1.6%)	3	(6.8%)	0	(0%)	N/A
Hookah/water pipe smoking	4	(2.1%)	2	(4.5%)	2	(1.4%)	N/A
Electronic cigarette smoking	34	(17.6%)	13	(29.5%)	21	(14.1%)	**0.032**
Marijuana Smoking	29	(15%)	14	(31.8%)	15	(10.1%)	**0.001**
Smoke other drugs	1	(0.5%)	0	(0%)	1	(0.7%)	N/A
Wood stove or fireplace	7	(3.6%)	1	(2.3%)	6	(4%)	N/A
Incense or candles	95	(49.2%)	21	(47.7%)	74	(49.7%)	0.957
Burn food	80	(41.5%)	20	(45.5%)	60	(40.3%)	0.660
Gas heater	18	(9.3%)	3	(6.8%)	15	(10.1%)	0.769^#^
Fry or sauté food with oil	167	(86.5%)	41	(93.2%)	126	(84.6%)	0.223
Gas/propane appliance to cook	126	(65.3%)	30	(68.2%)	96	(64.4%)	0.780
Electric appliance to cook	179	(93.2%)	40	(90.9%)	139	(93.9%)	0.722
Spray products	138	(71.5%)	31	(70.5%)	107	(71.8%)	1.000
Vacuum/dust/sweep	188	(97.4%)	42	(95.5%)	146	(98%)	0.697
**Ventilation activities**							
Central air	41	(21.2%)	3	(6.8%)	38	(25.5%)	**0.030**^#^
Air purifier	16	(8.3%)	4	(9.1%)	12	(8.1%)	0.765^#^
Exhaust fan in the kitchen	116	(60.1%)	28	(63.6%)	88	(59.1%)	0.712
Window fan or window air conditioner	54	(28.3%)	16	(37.2%)	38	(25.7%)	0.198
Open a window	184	(95.3%)	42	(95.5%)	142	(95.3%)	1.000
Open an exterior door	187	(96.9%)	43	(97.7%)	144	(96.6%)	1.000

p values come from test of equal proportions using the prop.test() function in R unless otherwise indicated. Variables with fewer than 10 responses were not tested.

^#^ indicates p values that come from Fisher's Exact test, used when cell sizes were <5

^¶^ indicates p values that come from chi-squared tests

^‡^ p-values results from two-sample t-tests with equal variance

Bolded p-values indicate statistical significance at an alpha < 0.05

The R functions used to test for statistical significance were: prop.test(), fisher.test(), t.test(), and chisq.test()

### Empirical particle distributions

#### Effects of room and home size

The size of a residence is an important consideration when designing pollutant or air-quality feedback for household residents. Low-SES populations may have higher pollutant levels due to having smaller homes with lower mixing volumes. From the physics of particle dynamics [42,43], we anticipate, for consistent source and ventilation activity, magnitude, and mixing, that particle concentrations will be larger in homes with smaller volumes and smaller in homes with larger volumes. Henschen et al. (1997) found that a smaller size of dwelling for 602 school children was associated with higher passive smoking exposures [52]. For individual homes, we expect to observe a clear result as predicted by the mass balance equation, although compartment and proximity effects (non-instantaneous mixing of pollutants) introduce more complexity [16,53,54]. Concentrations nearer an active or recently-active source, or the room in which a source is active, are higher than in further away or separate rooms. Closed doors also inhibit the flow of air and pollutants, increasing the compartmental character of a home [55]. However, for population data, whether we see a trend in particle levels versus space volume depends on how consistent overall source-, mixing-, and ventilation-activity are from one home to the next.

**Fig 1:**
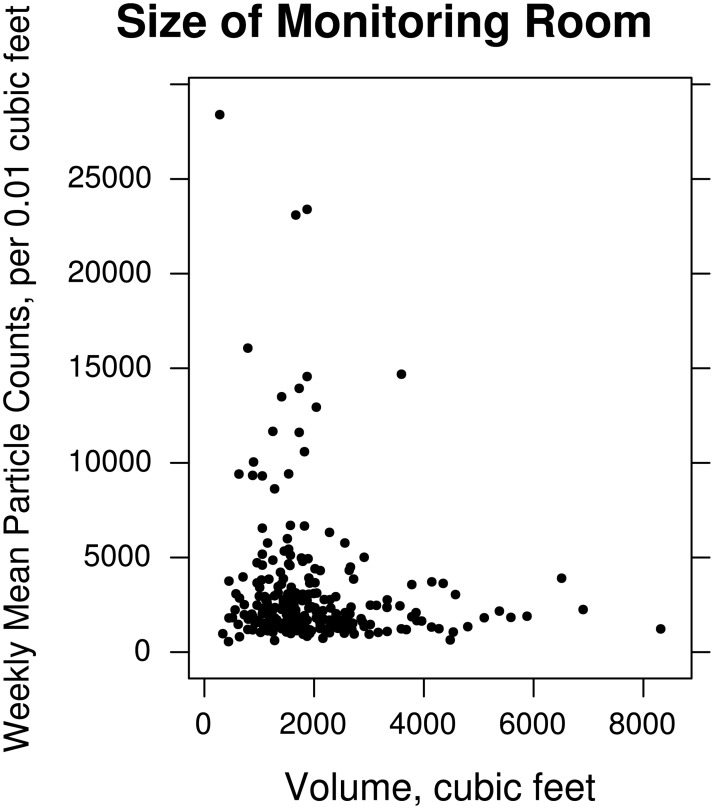
Effect of room size on particle levels. Scatterplot of mean particle counts as a function of monitoring-room volume in cubic feet (n = 257). The horizontal axis shows the volume of the monitored room in cubic feet. The vertical axis shows the mean number of particles taken over a week-long period in units of counts of particles per 0.01 cubic feet with diameters between 0.5 and 2.5 micrometers (Dylos™ Air Quality Monitor).

Fig 1 shows the weekly mean particle concentration as a function of the volume of monitored room. Small volumes have a higher mean particle level with an overall non-linear trend. However, the intermediate region with many data points has no evident trend. This finding is consistent with the variation in sources and ventilation between homes and the smudging of differences for homes with approximately the same layouts and overall volumes. However, we see the expected trend of higher/lower levels with lower/higher monitoring-room volumes emerging at the extremes. In the extreme cases, volumes reach limits and/or home layouts may be substantially different from other homes, so that as volume increases, we see particle levels decrease to near-background levels. Similarly, at lower room volumes we see some homes have much higher particle levels than others. While it is possible that source activity or ventilation could play a role in these observations, it is also likely that, since activities like smoking, cooking, and window-or-fan-based ventilation are not likely to be very different between homes, that volume of homes plays a role.

**Fig 2:**
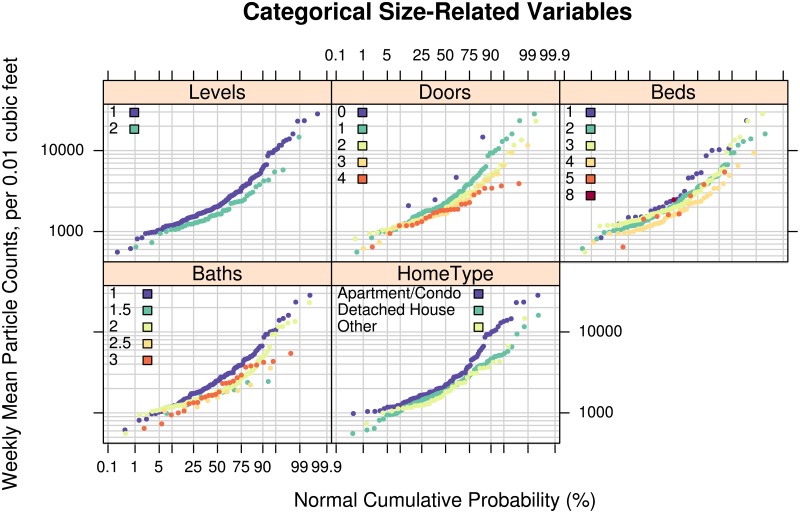
Effect of home size on particle levels. Log-probability plots comparing the empirical cumulative distributions of mean particle counts measured in the living areas of 254 to 262 participants’ homes for 5 selected categorical home characteristics: the number of levels in the home, the number of (exterior) doors, the number of bedrooms and bathrooms, and the type of home. The vertical axis shows the mean number of particles over a week-long period in units of counts of particles per 0.01 cubic feet with diameters between 0.5 and 2.5 micrometers (Dylos™ Air Quality Monitor). The horizontal axis shows the cumulative empirical probability of having a given particle count value.

Fig 2 compares weekly mean particle count distributions for 6 home-size-related factors: Number of Levels; Number of Exterior Doors; Number of Bedrooms, Number of Bathrooms, and the Type of Home (apartment/detached). The range of observed weekly mean particle counts for the total sample was 556 to 28,400 counts per 0.01 cubic feet. The number of levels in a home indicates overall home size and a larger mixing volume for diluting particle emissions. The population distributions for 1 and 2 levels appear parallel on the Fig 2 log-probability plot with particle counts for 1 level greater than for 2 levels. This finding is consistent with the theoretical expectation. Homes with 1 versus 2 levels near the median particle concentration appear different on the order of 100 counts, whereas at the upper extremes of the distribution, the mean levels can be different by 1,000 to 10,000 counts. The difference in particle distributions for homes with different numbers of exterior doors bedrooms or bathrooms displays a similar pattern. In general, with more doors, bedrooms, or bathrooms, all indicators of larger home size, the weekly mean particle concentrations are lower by as much as 1,000 to 10,000 counts at the extremes of the distribution. The type of home also appears to influence particle concentration in the upper quartiles of the distributions (difference of 1,000 counts) with apartments and condos having higher concentrations than detached houses.

#### Smoking source activity

**Fig 3:**
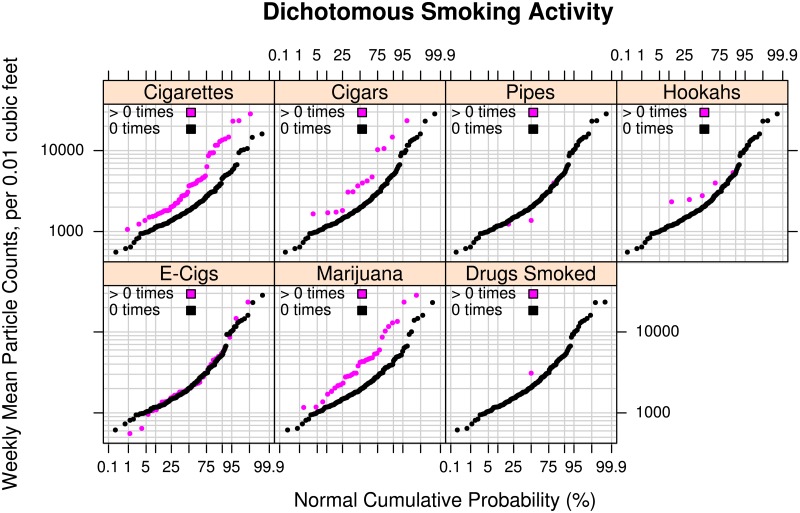
Effect of smoking activity on particle levels. Log-probability plots comparing the empirical cumulative distributions of mean particle counts measured in the living areas of 220 to 262 participants’ homes for number of occurrences of 7 smoking-related dichotomous weekly activities (i.e., “0 times” versus “> 0 times”): Cigarettes, Cigars, Pipes, Hookahs, Electronic Cigarettes, Marijuana, and Other Smoked Drugs. The lowest sample was for Marijuana (n = 220) and Other Drugs Smoked (n = 222) questions with other samples of at least n = 256. The vertical axis shows the mean number of particles over a week-long period in units of counts of particles per 0.01 cubic feet with diameters between 0.5 and 2.5 micrometers (Dylos™ Air Quality Monitor). The horizontal axis shows the cumulative empirical probability of having a given particle count value.

The central aim of the present work is to explore occupant-controlled household activities that influence levels of indoor particles in a large sample of homes (*n* = 262). Smoking source activity is of primary interest since it generates large emissions and can be relocated or reduced based on household practices. To explore the effect of smoking on indoor fine particle levels, we plotted the raw distributions of weekly mean particle concentrations for the dichotomous occurrence of 7 types of smoking during the week (Fig 3) to identify, by visual inspection, those that had a substantial impact on the distribution. Distributions are plotted for homes reporting “0 times” or “> 0 times” of use during the week. Clearly, indoor use of cigarettes resulted in higher particles levels in the population. The “> 0 times” of indoor smoking group (*n* = 55) had median levels close to 2000 counts greater than the “0 times” group (*n* = 201) with the distributions diverging so that at the upper tail indoor smoking could contribute up to 20,000 more weekly mean counts. This finding is consistent with previous research that shows smoking cigarettes elevates particle levels in individual rooms of homes on the order of 100’s of μg/m^3^ (e.g., Ott et al. 2003 [16]), which are roughly equivalent to counts on the order of 20,000 counts per 0.01 cubic feet as measured by the Dylos Air Quality Monitor for secondhand smoke [40]. In California, medical marijuana use is legal. A sizable number of homes (*n* = 33) reported smoking marijuana from 1 to 7 times per week (“> 0 times”) with higher levels at all percentiles than homes not reporting marijuana smoking. While smoking of marijuana is known to produce sizable particle emissions and constituents comparable to tobacco smoke [56], not as much is known about population-scale indoor marijuana levels in homes. It appears from our distribution results that it may contribute to indoor particle pollution as much as cigarettes do. Like marijuana, cigars and incense are also known to be sources of indoor particle concentrations comparable to cigarettes [57]. Our sample had relatively few homes that reported cigar use (*n* = 14), but those that did had higher levels than those that did not at an amount similar to the difference for cigarettes (median was 2,000 counts higher). Those at the high end had weekly means that were 1,000’s of counts greater. Data on pipes, hookahs, and smoking other drugs were too sparse (*n* = 1 to 5 reporting usage) to draw meaningful conclusions. If data on these sources are desired in future studies, it is advisable to focus on such homes using a concentrated sample. We observed no apparent difference in the weekly mean particle distribution between 43 homes reporting any electronic cigarette usage and those reporting none.

#### Other particle-source activity

**Fig 4:**
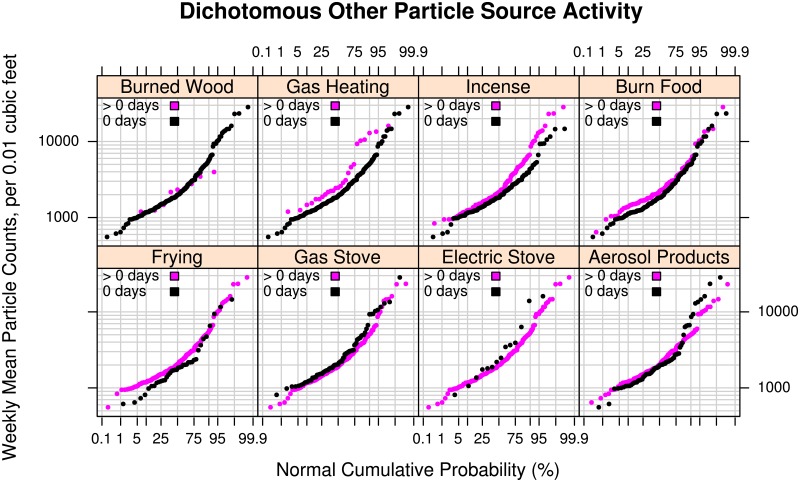
Effect of other sources on particle levels. Log-probability plots comparing the empirical cumulative distributions of mean particle counts measured in the living areas of 260 to 262 participants’ homes for 8 dichotomous weekly cooking-, heating-, or recreation-related combustion activities (i.e., use on “0 days” versus “> 0 days” (1 to 7 days inclusive) that generate particles: Burned Wood, Gas Heating, Incense, Burn Food, Frying, Gas Stove, Electric Stove, and Aerosol Consumer Products. The vertical axis shows the mean number of particles over a week-long period in units of counts of particles per 0.01 cubic feet with diameters between 0.5 and 2.5 micrometers (Dylos™ Air Quality Monitor). The horizontal axis shows the cumulative empirical probability of having a given particle count value.

Fig 4 presents particle distributions for homes with 8 particles sources related to heating (wood or gas), cooking (frying or burning food, cooking with gas or electric), or other sources (incense, spray “aerosol” products). The burning of wood and gas emit particulate matter and electric stoves emit fine and ultrafines [58]. Cooking, especially frying, is a well-known source of particles (e.g., [59–61]) to which the Dylos Air Quality Monitor monitor is very sensitive (see [40]). For our sample, we did not observe an impact of wood burning, although the sample of those reporting wood burning was very small (*n* = 9). Participants reporting any use of gas heating over a week had a somewhat larger sample size (*n* = 25) and a larger median particle count of about 500 versus those who did not use gas heating. At the upper end of the distribution, gas heating could contribute 5,000+ counts (weekly mean) above homes with no gas heating. Similarly, the distribution from homes that reported incense use (*n* = 120) diverged from homes that didn’t (*n* = 140) at the upper half of the distribution, with differences of 1000's of counts (weekly mean). This divergence point at ~2000 counts (the approximate median of both distributions) may distinguish heavy versus light incense usage in that the 50% of homes with light incense usage may be more similar to homes with no usage than the homes with heavy incense usage. For participants who reported burning food at least once during the week (*n* = 105), the weekly particle mean at the lower end of the distribution was on the order of 100 counts greater than those who did not report burning food. This result is consistent with a small increase in the weekly mean due to, for example, burning toast on 1 or 2 occasions. Frying activity (*n* = 224) also showed small elevated levels up to 100's of particle counts at the lower end of the distribution relative to homes that reported no frying activity (*n* = 38). The use of aerosol products did not seem to affect the distribution, with the small sample size at the upper tail possibly masking effects of heavy use.

#### Ventilation activity

**Fig 5:**
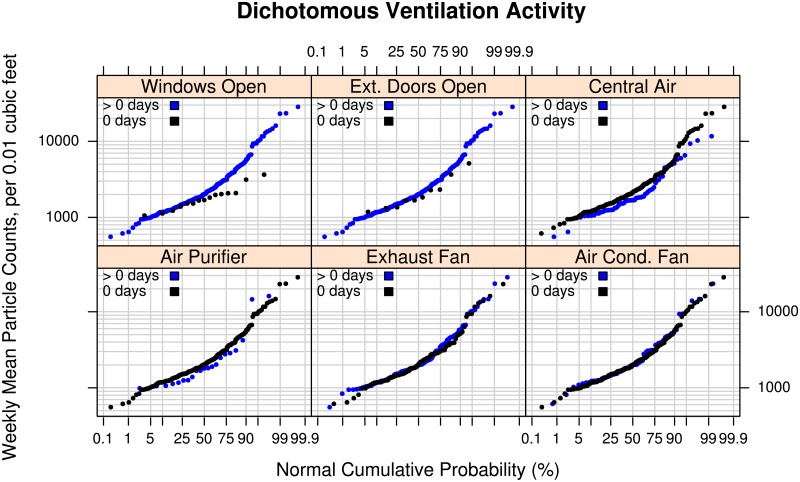
Effect of ventilation activity on particle levels. Log-probability plots comparing the empirical cumulative distributions of mean particle counts measured in the living areas of 260 to 262 participants’ homes for 6 dichotomous weekly ventilation-related activities (i.e., use on “0 days” versus “> 0 days” (1 to 7 days inclusive): Windows Open, Exterior Doors Open, Central Air, Air Purifier, Exhaust Fan, Air Conditioning Fan. The vertical axis shows the mean number of particles over a week-long period in units of counts of particles per 0.01 cubic feet with diameters between 0.5 and 2.5 micrometers (Dylos™ Air Quality Monitor). The horizontal axis shows the cumulative empirical probability of having a given particle count value.

Fig 5 shows empirical particle distributions for whether certain ventilation-related activities occurred on at least one day during the week. From a theoretical perspective, based on the mass balance equation cited above, increased ventilation is expected to decrease particle levels due to natural air exchange with the outdoors or the use of localized exhaust fans or central air (forced air flow). The use of central air can also serve to spread tobacco smoke and other air contaminants more quickly throughout a house [53]. However, we also expect that consistency of coincidence between enhanced ventilation with source activity and duration of ventilation activity will determine whether protective effects of ventilation can be observed for weekly mean particle levels in a sample of homes. In our sample, we did not observe clear-cut effects of any reported ventilation activity. Most of the sample (94% to 95%) reported having windows open (*n* = 246) and exterior doors open (*n* = 242) for one or more days during the week of monitoring. The upper parts of the distribution of weekly particle mean counts for the very small number of homes that did not have windows open at least once may have a protective effect, although the sample size is too small to draw generalizable conclusions. Scenarios where this may occur might be when outdoor pollution is high enough that keeping windows closed reduces the transfer rate of outdoor pollution entering the home. The reported use of an air purifier, exhaust fan, or air conditioner fan had no observable effect. The distribution of weekly particle means for users of central air (for at least one day) is lower by up to 100 counts in the middle of the distribution. This effect may be due to the enhanced air flow, mixing, and dilution of indoor-generated particles. Overall, reported ventilation activities showed very small effects on weekly population particle counts.

### Statistical results

Whereas graphical visualization provides a data overview for observing raw relationships, sample sizes, and distributional features, model-based analysis offers a way to quantitatively confirm and summarize important findings, and to control for the influence of other variables. Simple regression results are shown in Table 4 in which we identify the variables that we used in the step-wise model-building procedure. Multivariable linear regression results for the final model, including all variables identified from model-building, are shown in Table 5.

**Table 4:** Simple linear regression of weekly log-transformed mean particle counts on each potential correlate, *n* = 193 homes.

	β^a^	se	p-value	r^2^
**Home characteristics**				
Room Volume (1000 ft^3^)	-0.09	(0.04)	**0.029**†	0.025
Number of levels	-0.15	(0.14)	0.290	0.006
Number of rooms	-0.03	(0.02)	0.188†	0.009
Number of exterior doors	-0.16	(0.04)	**0.000**†	0.064
Number of bedrooms	-0.06	(0.05)	0.218	0.008
Number of bathrooms	-0.17	(0.07)	**0.025**†	0.026
Distance from Roadway			0.260	0.014
Roadway <50 feet				
Roadway 50–100 feet	0.20	0.12		
Roadway >100 feet	0.09	0.12		
Home Type			0.066†	0.034
Condo/Apt.				
Detached house	-0.23	0.10		
Other	-0.14	0.15		
**Indoor particle generating activities**				
Cigarette smoking	0.56	(0.11)	**0.000**†	0.126
Electronic cigarette smoking	0.05	(0.13)	0.698	0.001
Marijuana Smoking	0.58	(0.13)	**0.000**†	0.100
Gas heater	0.15	(0.16)	0.346	0.005
Incense or candles	0.26	(0.09)	**0.006**†	0.040
Burn food	0.14	(0.10)	0.153†	0.011
Fry or sauté food with oil	0.43	(0.14)	**0.002**†	0.049
Gas/propane appliance to cook	-0.03	(0.10)	0.733	0.001
Electric appliance to cook	-0.20	(0.19)	0.296	0.006
Spray products	-0.06	(0.11)	0.540	0.002
Vacuum/dust/sweep	0.56	(0.30)	0.061†	0.018
**Ventilation activities**				
Central air	-0.12	(0.12)	0.302	0.006
Air purifier	-0.09	(0.17)	0.617	0.001
Exhaust fan in the kitchen	0.04	(0.10)	0.672	0.001
Window fan or window air conditioner	-0.04	(0.11)	0.709	0.001
Open a window	0.23	(0.23)	0.303	0.006
Open an exterior door	0.24	(0.27)	0.390	0.004

Bolded p-values indicate statistical significance at an alpha < 0.05

^a^ unstandardized beta coefficients

se = the standard error for the regression

r2 is the coefficient of determination for the regression

^†^ indicates variables that were included in the model building procedure (p-values < 0.20)

Indoor particle generating activities and ventilation activities are dichotomized variables

**Table 5:** Multi-variable linear regression results of weekly log-transformed mean particle counts on variables selected during model building.

	β^a^	se^b^	p
**Home characteristics**			
Room Volume (1000 ft^3^)	-0.01	0.03	0.669
Number of Rooms	0.03	0.02	0.129
Number of Bathrooms	-0.25	0.08	**0.003**
Number of Exterior Doors	-0.08	0.04	**0.034**
**Indoor particle generating activities**			
Cigarette smoking	0.45	0.12	**<0.001**
Marijuana Smoking	0.52	0.12	**<0.001**
Incense or candles	0.26	0.08	**0.002**
Burn food	0.14	0.09	0.101
Fry or sauté food with oil	0.27	0.11	**0.020**
Vacuum/dust/sweep	0.52	0.14	**<0.001**
	*n* = 193	r^2^ = 0.35	Adj. r^2^ = 0.314

Bolded p-values indicate statistical significance at an alpha < 0.05

^a^ Beta coefficients can be used to compute the percentage change in geometric mean particle counts associated with a 1 unit increase in number of rooms, number of bathrooms, or the number of doors leading outside; a 1000 ft3 increase in room volume; or with the presence of (vs. the absence of) selected indoor particle generating or ventilation activities

^b^ Heteroskedascity-consistent standard errors

The statistical results for particle counts with home-size-related variables (Table 5) confirm our graphical analysis. Each additional exterior door was associated with a 7.7% ([e^-0.08^–1]*100) reduction in geometric mean 1-week particle counts (β = -0.08, p = 0.034), after adjusting for all other correlates. Each additional bathroom was associated with a 22.1% reduction in geometric mean 1-week particle counts (β = -0.25, p = 0.003). There were no statistically significant associations between geometric mean particle levels and room volume or number of rooms after adjustment for all other variables.

Similarly, the modeling results for particle sources agreed broadly with our graphical analysis. Statistically significant increases in indoor particle counts were observed among homes that reported indoor cigarette or marijuana smoking, burning incense and/or candles, frying food in oil, or cleaning the house by vacuuming, dusting or sweeping. Smoking cigarettes indoors (compared to not smoking cigarettes indoors) was associated with a 56.8% ([e^0.45^–1]*100) increase in geometric mean particle counts (β = 0.45, p < 0.001) while smoking marijuana indoors was associated with a 68.2% increase (β = 0.52, p < 0.001). There were no statistically significant associations between geometric mean particle levels and burning food after adjustment for all other variables. Homes that reported cleaning in the last 7 days had 68.2% higher 1-week geometric mean particle counts than homes that did not report cleaning (β = 0.52, p < 0.001). Frying with oil was also associated with increased geometric mean particle levels with any indoor frying being associated with a 31.0% increase in geometric mean particle levels compared to homes that did not report frying food with oil (β = 0.27, p = 0.020).

From our tests that explored central air (p = 0.94), air purifier (p = 0.45), exhaust fan in kitchen (p = 0.13), window fan or window air conditioner (p = 0.10), open a window (p = 0.11), and open an exterior door (p = 0.96) as effect modifiers of the association between reported indoor smoking and mean particle levels, none were statistically significant. The relatively small number of homes in subgroup analyses is one possible explanation for the null results, particularly for tests of using an exhaust fan in kitchen, using a window fan or window air conditioner, and opening windows.

### Additional tests

From our tests that explored ventilation activities as effect modifiers of the association between reported indoor smoking and mean particle levels, we found that none of the 6 activities were statistically significant (p’s < 0.05). In another suite of tests comparing homes that were included in statistical analyses to homes that were omitted due to complete-case-only analysis, we found no differences for indoor smoking or any other indoor-particle-generating activities (see S3 Table). We also did not observe statistically significant differences in ventilation activities. Small, yet statistically significant, differences were identified in several variables related to home size, particularly with room volume, the number of levels, rooms, and bedrooms, and with the proportion of home types. For all differences, the 69 homes omitted from the complete cases analysis sample were, on average, larger and had disproportionately higher “other” home types.

## Discussion

This article explores how indoor air quality is affected by occupant activities and home characteristics for a large number of smoking households with children under 14. From the viewpoint of epidemiologists, large samples of participants are desired to draw population-level conclusions and develop scalable interventions. However, for air-quality-targeted studies using air monitoring instrumentation, large studies are hindered due to the expense of hardware and quality-assured deployment. This study has made successful use of an inexpensive, unobtrusive monitor that was deployed for long periods in nearly 300 homes (see Klepeis et al. 2013 [17] for details on the monitoring equipment). Thus, it is possible to report week-long airborne particle measurements for a large sample that is expected to be representative of at-risk, low-income families with young children in San Diego and similar metropolitan regions.

This work provides a resource to those who may be undertaking air quality interventions for tobacco smoke or other pollutant sources. At the confluence of public health, exposure dynamics, and building science, it contributes new data to inform future air quality interventions about fundamental driving forces for air quality (and particles in particular). This study’s findings may be used to develop new hypotheses and inform future epidemiological and health studies in developing effective interventions for indoor air pollution, especially for tobacco-related risks for children. The results may provide insight and training to those involved in air-quality interventions, by summarizing influential variables in a large sample of homes. Smoking, whether cigarettes or marijuana, is a major source for this study population, whereas electronic cigarettes are not. Larger samples of more specialized sources, such as cigars, pipes, and hookahs, are required to draw conclusions. In keeping with expectations and prior studies, outdoor smoking is clearly protective in terms of mitigating indoor particle levels and larger home and room size can be associated with lower levels. In addition, our results indicate that cooking with oil, independent of whether food is burned, is associated with elevated particle counts and that cleaning by vacuuming/dusting/sweeping is also associated with elevated particle counts.

Relationships between air quality indicators (like airborne particles) and physical characteristics of homes or occupant behaviors are vital, but knowledge on the intensity of emissions and exposures is also important in providing feedback to intervention participants and broadly gauging the seriousness of health risk. The Dylos monitor and other real-time particle monitors are not best-suited for estimating absolute mass particle concentration due to differential sensitivity of the monitor to source types and removal rates [40,61]. While mass levels are typically assessed using a “gold standard” filter-and-pump approach, the Dylos may still be used as an approximate indication of the magnitude of indoor levels and occupants exposures. For example, weekly mean particle counts for our sample ranged from 556 to 28,400 counts per 0.01 cubic feet (particles 0.5 to 2.5 microns in diameter). For fresh cigarette smoking emissions, incense, and some types of cooking, Dacunto et al. (2015) reported mean count-to-mass conversion factors of roughly 0.02 to 0.03, giving weekly mean concentrations for our sample that range from a low of 11 to 16 micrograms per meter cubed (μg/m^3^) to a high of 568 to 852 μg/m^3^ [40]. Note that the assumption of fresh emissions may overestimate the peak mass levels for homes with source emissions that may take hours to decay to background levels (e.g., see reported experiment by Ott et al. 2003 [16]). However, it is clear that indoor sources such as cigarettes, marijuana, and incense are associated with substantially-elevated particle levels that may pose a risk to health, and Dylos measurements can be used as reliable feedback to intervention participants to indicate particles rising to concerning levels. Low ambient-level particles are in the range of 10 μg/m^3^, which corresponds roughly to Dylos particle counts of 300 to 500. The 24-hour U.S.EPA standard for fine particles is only 35 μg/m^3^. In addition, the mean cigarette-long levels observed in moving cars, with the AC on, can exceed 400 μg/m^3^ [62] and mean levels in smoky areas of casinos have been reported at 63 μg/m^3^ [63]. These reference levels, and similar ones for other sources, may give intervention participants a sense for how high the particle levels may be in their home.

Future studies should examine marijuana use and its impact on indoor air quality in low-income homes with smokers, and consider this source explicitly in air-quality or environmental interventions. One of our central and new findings based on modeling and graphical plots is that marijuana use is associated with significantly higher particle counts for both indoor cigarette smokers and outdoor cigarette smokers even after accounting for home characteristics, other particle generating behaviors, and ventilation activities. Marijuana was the most commonly-used illicit drug among people aged 12 and older in 2013 and use is on the rise from 5.8% in 2002 to 7.5% in 2013 (SAMHSA, 2014). In the U.S., reported unintentional marijuana exposure to children aged 9 years and under to the poison control center increased by 30% from 2005 to 2011 partially due to legalization of marijuana use for medical and recreational purposes. California, where medical marijuana use is legal, has the fifth highest reported exposure (4.9 per 100,000) in the U.S. [64]. Symptoms of ingestion or passive inhalation exposure among children under 5 years of age include lethargy, bradycardia, somnolence, and sleepiness [65]. Recently, Wang et al. (2016) found that brief exposures to secondhand marijuana smoke impaired vascular endothelial function in rats, suggesting that secondhand marijuana smoke may have cardiotoxic effects similar to those from secondhand tobacco smoke [66].

## Limitations

Our study has several limitations. The size of home was determined by proxy measurements (number of rooms, doors, etc.) rather than direct measurement. Our measurements of ventilation and source activity were determined from broad interview questions, rather than more detailed time-activity or other objective measures, which may have limited our ability to see effects. The efficiency of specific ventilation measures in a home and the coupling of ventilation activity with specific sources are expected to be important factors. Data on indoor versus outdoor smoking behavior was based on report at an initial interview rather than direct observation by trained observers or sensor objective measures. Specific data on the number of daily cigarettes and the duration of smoking were not gathered. The reporting of indoor smoking and other sensitive information such as marijuana use and other drug use may be underreported due to social-desirability bias and other biases. For a subset of homes in this study, we did not have authorization to ask questions about marijuana and other illicit drug use, and therefore those homes were omitted from statistical model building, but included in graphical analyses. We studied a base sample of 262 homes that excluded the first 36 pilot homes and was further constrained for certain analysis due to missing data on some variables. Despite these potential biases, we observed important effects of cigarette and marijuana use on particle counts. Under-reporting marijuana smoking and indoor cigarette smoking would likely attenuate effect sizes, indicating that our results are probably underestimates. In addition, the proximity of sources to the Dylos monitor was not recorded and could have impacted the weekly mean particle counts. More proximate sources could have resulted in higher counts relative to a “well-mixed” emissions condition.

## Conclusions

Our results confirm the importance of smoking, cooking, and cleaning in the elevation of indoor particle levels, as reported by other investigators. We report new findings that marijuana may lead to as comparatively large an increase in weekly particle levels as cigarettes and deserves focused future study. Smokers who do not smoke indoors demonstrated better indoor air quality relative to homes where smoking occurs indoors, a finding that is consistent with engineering principles but also manifests in population-level data. Broadly-assessed ventilation activities did not appear to have a mitigating effect on indoor air particles. Our study illustrates how inexpensive air quality instrumentation can be successfully deployed in large samples for epidemiological and behavioral studies. Future studies of air quality in at-risk populations may use our results to better focus interventions, to consider which key influential variables to include on interviews and questionnaires, and to design sampling procedures to include sub-groups of interest.

## Supporting information

S1 Table Sample sizes for dichotomous variables used in graphical analysis. (DOCX)

S2 Table Sample sizes for categorical variables used in graphical analysis. (DOCX)

S3 Table Housing characteristics, particle generating- and ventilation-activities compared for the final analytic sample versus homes excluded for complete case analysis. (DOCX)

S4 Table Variable names and codes in the raw data file. (DOCX)

S1 File Measure interview. (RTF)

S2 File Measure codebook. (RTF)

S3 File Raw data file in CSV format. (CSV)
