# Supplementary material for: Fine particles in homes of predominantly low-income families with children and smokers: Key physical and behavioral determinants to inform indoor-air-quality interventions
Source: PLoS One. 2017 May 17;12(5):e0177718. doi: 10.1371/journal.pone.0177718 (PMC5435241; doi:10.1371/journal.pone.0177718)
Supplement: S1 Table — (DOCX) [file pone.0177718.s001.docx]

**S1 Table. Sample sizes for dichotomous variables used in graphical analysis**

| **Variable** | **> 0 times** | **0 times** |
| --- | --- | --- |
| Cigarettes | 55 | 201 |
| Cigars | 14 | 244 |
| Pipes | 3 | 255 |
| Hookahs | 5 | 251 |
| E-Cigs | 43 | 213 |
| Marijuana | 33 | 187 |
| Drugs Smoked | 1 | 221 |
|  | **> 0 days** | **0 days** |
| Burned Wood | 9 | 253 |
| Gas Heating | 25 | 237 |
| Incense | 120 | 140 |
| Burn Food | 105 | 156 |
| Frying | 224 | 38 |
| Gas Stove | 177 | 85 |
| Electric Stove | 246 | 14 |
| Aerosol Products | 185 | 77 |
| Dusting | 252 | 9 |
| Windows Open | 246 | 15 |
| Ext. Doors Open | 252 | 10 |
| Central Air | 64 | 197 |
| Air Purifier | 21 | 241 |
| Exhaust Fan | 155 | 106 |
| Air Cond. Fan | 72 | 188 |

* These variables were omitted from the raw data base for human subject protection.
