# Supplementary material for: Fine particles in homes of predominantly low-income families with children and smokers: Key physical and behavioral determinants to inform indoor-air-quality interventions
Source: PLoS One. 2017 May 17;12(5):e0177718. doi: 10.1371/journal.pone.0177718 (PMC5435241; doi:10.1371/journal.pone.0177718)
Supplement: S2 Table — (DOCX) [file pone.0177718.s002.docx]

**S2 Table. Sample sizes for categorical variables used in graphical analysis**

| **Variable** | **1** | **2** |  |  |  |  |
| --- | --- | --- | --- | --- | --- | --- |
| Levels | 212 | 47 |  |  |  |  |
|  |  |  |  |  |  |  |
|  | **0** | **1** | **2** | **3** | **4** |  |
| Doors | 4 | 82 | 93 | 49 | 26 |  |
|  |  |  |  |  |  |  |
|  | **1** | **2** | **3** | **4** | **5** | **8** |
| Beds | 25 | 101 | 82 | 43 | 8 | 1 |
|  |  |  |  |  |  |  |
|  | **1** | **1.5** | **2** | **2.5** | **3** |  |
| Baths | 111 | 8 | 106 | 9 | 26 |  |
|  |  |  |  |  |  |  |
|  | **Apartment/ Condo** | **Detached House** | **Other** |  |  |  |
| HomeType | 109 | 114 | 39 |  |  |  |
|  |  |  |  |  |  |  |
|  | **Non Missing** |  |  |  |  |  |
| RoomVolume | 257 |  |  |  |  |  |
