# Supplementary material for: Fine particles in homes of predominantly low-income families with children and smokers: Key physical and behavioral determinants to inform indoor-air-quality interventions
Source: PLoS One. 2017 May 17;12(5):e0177718. doi: 10.1371/journal.pone.0177718 (PMC5435241; doi:10.1371/journal.pone.0177718)
Supplement: S3 Table — (DOCX) [file pone.0177718.s003.docx]

**S3 Table. Housing Characteristics, Particle Generating- and Ventilation-Activities Compared for the Final Analytic Sample Versus Homes Excluded for Complete Case Analysis**

|  |  | Excluded for complete cases analyses, *n* = 69 | | | Analytic Sample *n* = 193 | |  |
| --- | --- | --- | --- | --- | --- | --- | --- |
|  |  | n Non-Missing | mean or n | (SD) or (%) | mean or n | (SD) or (%) | p |
| Home characteristics | |  |  |  |  |  |  |
|  | Room Volume (ft3) | 64 | 1734.9 | 819.42 | 2018 | 1213.68 | 0.037 |
|  | Number of levels | 66 | 1.3 | 0.46 | 1.1 | 0.35 | 0.010 |
|  | Number of rooms | 63 | 7.1 | 2.6 | 6.2 | 2.37 | 0.031 |
|  | Number of doors | 61 | 2.1 | 0.79 | 2 | 1.05 | 0.567 |
|  | Number of bedrooms | 67 | 2.9 | 0.99 | 2.6 | 1.03 | 0.030 |
|  | Number of bathrooms | 67 | 1.8 | 0.72 | 1.6 | 0.64 | 0.098 |
|  | Distance from Roadway | 67 |  |  |  |  | 0.086 |
|  | Roadway <50 feet |  | 43 | 64.2 | 112 | 58.0 |  |
|  | Roadway 50-100 feet |  | 18 | 26.9 | 41 | 20.7 |  |
|  | Roadway >100 feet |  | 6 | 9.0 | 40 | 21.2 |  |
|  | Home Type | 69 |  |  |  |  | 0.046 |
|  | Condo/Apt. |  | 22 | 31.9 | 87 | 45.1 |  |
|  | Detached house |  | 31 | 44.9 | 83 | 43.0 |  |
|  | Other |  | 16 | 23.2 | 23 | 11.9 |  |
| Indoor particle generating activities | |  |  |  |  |  |  |
|  | Cigarette smoking | 63 | 11 | 17% | 44 | 23% | 0.472 |
|  | Cigar smoking | 65 | 4 | 6% | 10 | 5% | 0.756 |
|  | Pipe tobacco smoking | 65 | 0 | 0% | 3 | 2% | 0.574 |
|  | Hookah/water pipe smoking | 64 | 1 | 2% | 4 | 2% | 1.000 |
|  | Electronic cigarette smoking | 63 | 9 | 14% | 34 | 18% | 0.675 |
|  | Marijuana Smoking | 27 | 4 | 15% | 29 | 15% | 1.000 |
|  | Smoke other drugs | 30 | 0 | 0% | 1 | 1% | 1.000 |
|  | Wood stove or fireplace | 69 | 2 | 3% | 7 | 4% | 1.000 |
|  | Incense or candles | 67 | 25 | 37% | 95 | 49% | 0.123 |
|  | Burn food | 68 | 25 | 37% | 80 | 41% | 0.593 |
|  | Gas heater | 69 | 7 | 10% | 18 | 9% | 1.000 |
|  | Fry or sauté food with oil | 69 | 57 | 83% | 167 | 87% | 0.552 |
|  | Gas/propane appliance to cook | 69 | 51 | 74% | 126 | 65% | 0.244 |
|  | Electric appliance to cook | 68 | 67 | 99% | 179 | 93% | 0.123 |
|  | Spray products | 69 | 47 | 68% | 138 | 72% | 0.707 |
|  | Vacuum/dust/sweep | 68 | 64 | 94% | 188 | 97% | 0.245 |
| Ventilation activities | |  |  |  |  |  |  |
|  | Central air | 68 | 23 | 34% | 41 | 21% | 0.056 |
|  | Air purifier | 69 | 5 | 7% | 16 | 8% | 1.000 |
|  | Exhaust fan in the kitchen | 68 | 39 | 57% | 116 | 60% | 0.800 |
|  | Window fan or window air conditioner | 69 | 18 | 26% | 54 | 28% | 0.849 |
|  | Open a window | 68 | 62 | 91% | 184 | 95% | 0.335 |
|  | Open an exterior door | 69 | 65 | 94% | 187 | 97% | 0.297 |

p values come from test of equal proportions using the prop.test() function in R.

* indicates p values that come from Fisher's Exact test, used when cell sizes were <=5

‡ p-values results from two-sample t-tests with equal variance

Bolded p-values indicate statistical significance at an alpha < 0.05
