# Supplementary material for: Fine particles in homes of predominantly low-income families with children and smokers: Key physical and behavioral determinants to inform indoor-air-quality interventions
Source: PLoS One. 2017 May 17;12(5):e0177718. doi: 10.1371/journal.pone.0177718 (PMC5435241; doi:10.1371/journal.pone.0177718)
Supplement: S4 Table — (DOCX) [file pone.0177718.s004.docx]

**S4** **Table. Variable Names and Codes in the Raw Data File**

| **Variable Description** | **Variable Name** | **Variable Codes** |
| --- | --- | --- |
| Home characteristics |  |  |
| Room Volume (ft^3^) | RoomVolume | [numeric] |
| Number of levels | Levels | [numeric] |
| Number of rooms | Rooms | [numeric] |
| Number of doors leading outside | Doors | [numeric] |
| Number of bedrooms | Beds | [numeric] |
| Number of bathrooms | Baths | [numeric] |
| Distance from Roadway | Roadway | [categorical] |
| Roadway <50 feet |  | “<50” |
| Roadway 50-100 feet |  | “50-100” |
| Roadway >100 feet |  | “>100” |
| Home Type | HomeType | [categorical] |
| Apartment/Condo |  | “Apartment/Condo” |
| Detached house |  | “Detached House” |
| Other |  | “Other” |
| Indoor particle generating activities over a week's monitoring |  | [dichotomous] |
| Cigarette smoking | cig7 | 0 = “0 times”; 1 = “> 0 times” |
| Cigar smoking | ciga7 | 0 = “0 times”; 1 = “> 0 times” |
| Pipe tobacco smoking | pipe | 0 = “0 times”; 1 = “> 0 times” |
| Hookah/water pipe smoking | hook | 0 = “0 times”; 1 = “> 0 times” |
| Electronic cigarette smoking | ecig | 0 = “0 times”; 1 = “> 0 times” |
| *Marijuana Smoking | mj7_ | 0 = “0 times”; 1 = “> 0 times” |
| *Smoke other drugs | drug | 0 = “0 times”; 1 = “> 0 times” |
| Wood stove or fireplace | wood | 0 = “0 days”; 1 = “>0 days” |
| Incense or candles | ince | 0 = “0 days”; 1 = “>0 days” |
| Burn food | food | 0 = “0 days”; 1 = “>0 days” |
| Gas heater | gash | 0 = “0 days”; 1 = “>0 days” |
| Fry or sauté food with oil | fry7 | 0 = “0 days”; 1 = “>0 days” |
| Gas/propane appliance to cook | gas7 | 0 = “0 days”; 1 = “>0 days” |
| Electric appliance to cook | elec | 0 = “0 days”; 1 = “>0 days” |
| Spray products | aero | 0 = “0 days”; 1 = “>0 days” |
| Vacuum/dust/sweep | dust | 0 = “0 days”; 1 = “>0 days” |
| Ventilation activities over a week's monitoring |  | [dichotomous] |
| Central air | cent | 0 = “0 days”; 1 = “>0 days” |
| Air purifier | pur7 | 0 = “0 days”; 1 = “>0 days” |
| Exhaust fan in the kitchen | exh7 | 0 = “0 days”; 1 = “>0 days” |
| Window fan or window air conditioner | acfa | 0 = “0 days”; 1 = “>0 days” |
| Open a window | win7 | 0 = “0 days”; 1 = “>0 days” |
| Open an exterior door | door | 0 = “0 days”; 1 = “>0 days” |
| Mean particle level (counts per 0.01 cubic feet) over a week's monitoring | OverallMean | [numeric] |

* These variables were omitted from the raw data base for human subject protection.
