# Supplementary material for: Fine particles in homes of predominantly low-income families with children and smokers: Key physical and behavioral determinants to inform indoor-air-quality interventions
Source: PLoS One. 2017 May 17;12(5):e0177718. doi: 10.1371/journal.pone.0177718 (PMC5435241; doi:10.1371/journal.pone.0177718)
Supplement: S1 File — (RTF) [file pone.0177718.s005.rtf]

Measure1 Interview 7-6-13 (English)
for the PLOS ONE manuscript entitled:
Fine Particles in Homes of Predominantly Low-Income Families with Children and Smokers: Key Physical and Behavioral Determinants to Inform Indoor-Air-Quality Interventions
by Neil Klepeis et al.  (pubs1@klepeis.net )
This is the Interview described in the above manuscript as implemented in QDS software:  http://www.novaresearch.com/QDS/.   It contains the skip instructions that are absent from the Codebook.
The interview was administered to participants on-site using laptop computers.

This research was supported with a grant from the National Heart, Lung, and Blood Institute of the National Institutes of Health from 2011 – 2015 under award number R01HL103684 (http://www.nhlbi.nih.gov/) to San Diego State University Research Foundation (Dr. M. Hovell, principal investigator, mhovell@cbeachsdsu.org).    Please address correspondence to Dr. Klepeis (pubs1@klepeis.net) or Dr. Hovell. 
SECTION PRE: Preliminary info entered prior to start of interview

PRE1.	SCRNID: 6 digit screening identification number, beginning with 700001.
Interviewer: pre-fill this number prior to starting the interview	__ __ __ __ __ __
PRE2.	HOMEID: 3 digit identification number for the home
Interviewer: pre-fill this number prior to starting the interview	__ __ __
PRE3.	INTRVWER. Interviewer Initials
INTERVIEWER: Please type in your two lowercase initials.
Note that the on-screen buttons are uppercase.	__ __
PRE4.	JUMP. Interviewer: Do you need to jump to another section? if so which one?  (Choose one)
	__ __	No, continue to Introduction
	__ __	RES: Residents in the Home
	__ __	CH: Child Health
	__ __	HCV: Heating, Cooling, or Ventilation
	__ __	VNT: Ventilation Behaviors
	__ __	PGA: Particle Generating Activities
	__ __	RTU: Residents' Tobacco Use
	__ __	SSE: Secondhand Smoke Exposure
	__ __	SR: Smoking Rules in the Home
	__ __	AV: Aversion to Secondhand Smoke Exposure
	__ __	SI: Social Influences
	__ __	DMG: Demographics
	__ __	ADM: Administrative Data
If PRE4 is equal to 1, then skip to instruction before INT1.
If PRE4 is equal to 2, then skip to instruction before RES1.
If PRE4 is equal to 3, then skip to instruction before CH1.
If PRE4 is equal to 4, then skip to instruction before HCV1.
If PRE4 is equal to 5, then skip to instruction before VNT1.
If PRE4 is equal to 6, then skip to instruction before PGA1.
If PRE4 is equal to 7, then skip to instruction before RTU1.
If PRE4 is equal to 8, then skip to instruction before SSE1.
If PRE4 is equal to 9, then skip to instruction before SR1.
If PRE4 is equal to 10, then skip to instruction before AV1.
If PRE4 is equal to 11, then skip to instruction before SI1.
If PRE4 is equal to 12, then skip to instruction before DMG1.
If PRE4 is equal to 13, then skip to ADM1.
SECTION INT: Introduction
My name is _____________and I want to remind you that the interview is confidential. You can refuse to answer any question. If you do not want to answer a question, just tell me and we will move to the next question. If you have any questions during the interview, please just ask.
INT1.	RECORD. My supervisor would like to make sure I have entered your answers correctly. Is it okay if I record this interview for my supervisor?  (Choose one)
	0	Participant opted for NO AUDIO RECORDING
	1	Participant agreed to have interview audio RECORDED
	2	Audio RECORDER NOT AVAILABLE for Interview
	7	Don't Know
	8	Refuse to Answer
	9	Not Applicable
SECTION RES: Residents in the Home
First I would like to ask about the residents in your household.
Interviewer: Section 1 of 12: RES: Residents in the Home
RES1.	TC. What is [the Target Child]'s First Name? [Ask TP to confirm spelling]
INTERVIEWER: Enter TC's name without asking, or ask TP to confirm spelling.
	__ __ __ __ __ __ __ __ __ __ __ __ __ __ __ __ __ __ __ __
RES2.	DBTC. What is [Response to RES1]'s Date of Birth? [Ask TP to confirm date]
INTERVIEWER: Birthdate should be four-digit year (YYYY), one or two-digit month (MM), and one or two-digit day (DD). Example: 3/14/2012. 
	__ __ / __ __ / __ __ __ __	mm / dd / yyyy
	2097	Don't Know (Year)
	2098	Refuse to Answer (Year)
	2099	Not Applicable (Year)
RES3.	SEXTC. What is [Response to RES1]'s Gender?
INTERVIEWER: Only ask gender when needed. 	1	Male
	2	Female
	7	Don't Know
	8	Refuse to Answer
	9	Not Applicable
RES4.	FNCHLD2. Child 2 First Name
INTERVIEWER: If there is no Child 2, enter "Not Applicable".
	__ __ __ __ __ __ __ __ __ __ __ __ __ __ __ __ __ __ __ __
If RES4 is equal to "Not Applicable" or RES4 is equal to "skipped", then skip to RES24.
RES5.	DBCHLD2. Child 2 Date of Birth
INTERVIEWER: Birthdate should be  four-digit year (YYYY), a one or two-digit month (MM), and a one or two-digit day (DD). Example: 3/14/2012. 
	__ __ / __ __ / __ __ __ __	mm / dd / yyyy
	2097	Don't Know (Year)
	2098	Refuse to Answer (Year)
	2099	Not Applicable (Year)
RES6.	SEXCHLD2. Child 2 Gender
INTERVIEWER: Only ask gender when needed. 	1	Male
	2	Female
	7	Don't Know
	8	Refuse to Answer
	9	Not Applicable
RES7.	RELCHLD2. Child 2 Relationship to TC
INTERVIEWER: Do not read response options to participant  (Choose one)
	01	Brother
	02	Sister
	03	Cousin
	04	Biological mother
	05	Stepmother
	06	Adoptive mother
	07	Foster Mother
	08	Biological father
	09	Stepfather
	10	Adoptive father
	11	Foster father
	12	Legal guardian
	13	Grandmother
	14	Grandfather
	15	Aunt
	16	Uncle
	17	Other (Specify)
	97	Don't Know
	98	Refuse to Answer
	99	Not Applicable
If RES7 is not equal to 17, then skip to RES9.
RES8.	OTHCHLD2. Specify Other Relationship to [Response to RES1]
	__ __ __ __ __ __ __ __ __ __ __ __ __ __ __ __ __ __ __ __
RES9.	FNCHLD3. Child 3 First Name
INTERVIEWER: If there is no Child 3, enter "Not Applicable".
	__ __ __ __ __ __ __ __ __ __ __ __ __ __ __ __ __ __ __ __
If RES9 is equal to "Not Applicable", then skip to RES24.
RES10.	DBCHLD3. Child 3 Date of Birth
INTERVIEWER: Birthdate should be  four-digit year (YYYY), a one or two-digit month (MM), and a one or two-digit day (DD). Example: 3/14/2012. 
	__ __ / __ __ / __ __ __ __	mm / dd / yyyy
	2097	Don't Know (Year)
	2098	Refuse to Answer (Year)
	2099	Not Applicable (Year)
RES11.	SEXCHLD3. Child 3 Gender
INTERVIEWER: Only ask gender when needed. 	1	Male
	2	Female
	7	Don't Know
	8	Refuse to Answer
	9	Not Applicable
RES12.	RELCHLD3. Child 3 Relationship to TC
INTERVIEWER: Do not read response options to participant  (Choose one)
	01	Brother
	02	Sister
	03	Cousin
	04	Biological mother
	05	Stepmother
	06	Adoptive mother
	07	Foster Mother
	08	Biological father
	09	Stepfather
	10	Adoptive father
	11	Foster father
	12	Legal guardian
	13	Grandmother
	14	Grandfather
	15	Aunt
	16	Uncle
	17	Other (Specify)
	97	Don't Know
	98	Refuse to Answer
	99	Not Applicable
If RES12 is not equal to 17, then skip to RES14.
RES13.	OTHCHLD3. Specify Other Relationship to [Response to RES1]
	__ __ __ __ __ __ __ __ __ __ __ __ __ __ __ __ __ __ __ __
RES14.	FNCHLD4. Child 4 First Name
INTERVIEWER: If there is no Child 4, enter "Not Applicable".
	__ __ __ __ __ __ __ __ __ __ __ __ __ __ __ __ __ __ __ __
If RES14 is equal to "Not Applicable", then skip to RES24.
RES15.	DBCHLD4. Child 4 Date of Birth
INTERVIEWER: Birthdate should be  four-digit year (YYYY), a one or two-digit month (MM), and a one or two-digit day (DD). Example: 3/14/2012. 
	__ __ / __ __ / __ __ __ __	mm / dd / yyyy
	2097	Don't Know (Year)
	2098	Refuse to Answer (Year)
	2099	Not Applicable (Year)
RES16.	SEXCHLD4. Child 4 Gender
INTERVIEWER: Only ask gender when needed. 	1	Male
	2	Female
	7	Don't Know
	8	Refuse to Answer
	9	Not Applicable
RES17.	RELCHLD4. Child 4 Relationship to TC
INTERVIEWER: Do not read response options to participant  (Choose one)
	01	Brother
	02	Sister
	03	Cousin
	04	Biological mother
	05	Stepmother
	06	Adoptive mother
	07	Foster Mother
	08	Biological father
	09	Stepfather
	10	Adoptive father
	11	Foster father
	12	Legal guardian
	13	Grandmother
	14	Grandfather
	15	Aunt
	16	Uncle
	17	Other (Specify)
	97	Don't Know
	98	Refuse to Answer
	99	Not Applicable
If RES17 is not equal to 17, then skip to RES19.
RES18.	OTHCHLD4. Specify Other Relationship to [Response to RES1]
	__ __ __ __ __ __ __ __ __ __ __ __ __ __ __ __ __ __ __ __
RES19.	FNCHLD5. Child 5 First Name
INTERVIEWER: If there is no Child 5, enter "Not Applicable".
	__ __ __ __ __ __ __ __ __ __ __ __ __ __ __ __ __ __ __ __
If RES19 is equal to "Not Applicable", then skip to RES24.
RES20.	DBCHLD5. Child 5 Date of Birth
INTERVIEWER: Birthdate should be  four-digit year (YYYY), a one or two-digit month (MM), and a one or two-digit day (DD). Example: 3/14/2012. 
	__ __ / __ __ / __ __ __ __	mm / dd / yyyy
	2097	Don't Know (Year)
	2098	Refuse to Answer (Year)
	2099	Not Applicable (Year)
RES21.	SEXCHLD5. Child 5 Gender
INTERVIEWER: Only ask gender when needed. 	1	Male
	2	Female
	7	Don't Know
	8	Refuse to Answer
	9	Not Applicable
RES22.	RELCHLD5. Child 5 Relationship to TC
INTERVIEWER: Do not read response options to participant  (Choose one)
	01	Brother
	02	Sister
	03	Cousin
	04	Biological mother
	05	Stepmother
	06	Adoptive mother
	07	Foster Mother
	08	Biological father
	09	Stepfather
	10	Adoptive father
	11	Foster father
	12	Legal guardian
	13	Grandmother
	14	Grandfather
	15	Aunt
	16	Uncle
	17	Other (Specify)
	97	Don't Know
	98	Refuse to Answer
	99	Not Applicable
If RES22 is not equal to 17, then skip to RES24.
RES23.	OTHCHLD5. Specify Other Relationship to [Response to RES1]
	__ __ __ __ __ __ __ __ __ __ __ __ __ __ __ __ __ __ __ __
RES24.	TP. Adult 1 (Target Parent) First Name
	__ __ __ __ __ __ __ __ __ __ __ __ __ __ __ __ __ __ __ __
RES25.	DBTP. Adult 1 (TP) Date of Birth
INTERVIEWER: Birthdate should be four-digit year (YYYY), one or two-digit month (MM), and one or two-digit day (DD). Example: 3/14/2012. 
	__ __ / __ __ / __ __ __ __	mm / dd / yyyy
	2097	Don't Know (Year)
	2098	Refuse to Answer (Year)
	2099	Not Applicable (Year)
RES26.	SEXTP. Adult 1 (TP) Gender
INTERVIEWER: Only ask gender when needed. 	1	Male
	2	Female
	7	Don't Know
	8	Refuse to Answer
	9	Not Applicable
RES27.	RELTP. Adult 1 (TP) Relationship to TC
INTERVIEWER: Do not read response options to participant  (Choose one)
	01	Brother
	02	Sister
	03	Cousin
	04	Biological mother
	05	Stepmother
	06	Adoptive mother
	07	Foster Mother
	08	Biological father
	09	Stepfather
	10	Adoptive father
	11	Foster father
	12	Legal guardian
	13	Grandmother
	14	Grandfather
	15	Aunt
	16	Uncle
	17	Other (Specify)
	97	Don't Know
	98	Refuse to Answer
	99	Not Applicable
If RES27 is not equal to 17, then skip to instruction before RES29.
RES28.	OTHTP. Specify Other Relationship to [Response to RES1]
	__ __ __ __ __ __ __ __ __ __ __ __ __ __ __ __ __ __ __ __
For the other adults in the household, we would only like to have their first name and relationship to [Response to RES1]
RES29.	FNADLT2. Adult 2 First Name
INTERVIEWER: If there is no Adult 2, enter "Not Applicable".
	__ __ __ __ __ __ __ __ __ __ __ __ __ __ __ __ __ __ __ __
If RES29 is equal to "Not Applicable", then skip to RES41.
RES30.	RELADLT2. Adult 2 Relationship to TC
INTERVIEWER: Do not read response options to participant  (Choose one)
	01	Brother
	02	Sister
	03	Cousin
	04	Biological mother
	05	Stepmother
	06	Adoptive mother
	07	Foster Mother
	08	Biological father
	09	Stepfather
	10	Adoptive father
	11	Foster father
	12	Legal guardian
	13	Grandmother
	14	Grandfather
	15	Aunt
	16	Uncle
	17	Other (Specify)
	97	Don't Know
	98	Refuse to Answer
	99	Not Applicable
If RES30 is not equal to 17, then skip to RES32.
RES31.	OTHADLT2. Specify Other Relationship to [Response to RES1]
	__ __ __ __ __ __ __ __ __ __ __ __ __ __ __ __ __ __ __ __
RES32.	FNADLT3. Adult 3 First Name
INTERVIEWER: If there is no Adult 3, enter "Not Applicable".
	__ __ __ __ __ __ __ __ __ __ __ __ __ __ __ __ __ __ __ __
If RES32 is equal to "Not Applicable", then skip to RES41.
RES33.	RELADLT3. Adult 3 Relationship to TC
INTERVIEWER: Do not read response options to participant  (Choose one)
	01	Brother
	02	Sister
	03	Cousin
	04	Biological mother
	05	Stepmother
	06	Adoptive mother
	07	Foster Mother
	08	Biological father
	09	Stepfather
	10	Adoptive father
	11	Foster father
	12	Legal guardian
	13	Grandmother
	14	Grandfather
	15	Aunt
	16	Uncle
	17	Other (Specify)
	97	Don't Know
	98	Refuse to Answer
	99	Not Applicable
If RES33 is not equal to 17, then skip to RES35.
RES34.	OTHADLT3. Specify Other Relationship to [Response to RES1]
	__ __ __ __ __ __ __ __ __ __ __ __ __ __ __ __ __ __ __ __
RES35.	FNADLT4. Adult 4 First Name
INTERVIEWER: If there is no Adult 4, enter "Not Applicable".
	__ __ __ __ __ __ __ __ __ __ __ __ __ __ __ __ __ __ __ __
If RES35 is equal to "Not Applicable", then skip to RES41.
RES36.	RELADLT4. Adult 4 Relationship to TC
INTERVIEWER: Do not read response options to participant  (Choose one)
	01	Brother
	02	Sister
	03	Cousin
	04	Biological mother
	05	Stepmother
	06	Adoptive mother
	07	Foster Mother
	08	Biological father
	09	Stepfather
	10	Adoptive father
	11	Foster father
	12	Legal guardian
	13	Grandmother
	14	Grandfather
	15	Aunt
	16	Uncle
	17	Other (Specify)
	97	Don't Know
	98	Refuse to Answer
	99	Not Applicable
If RES36 is not equal to 17, then skip to RES38.
RES37.	OTHADLT4. Specify Other Relationship to [Response to RES1]
	__ __ __ __ __ __ __ __ __ __ __ __ __ __ __ __ __ __ __ __
RES38.	FNADLT5. Adult 5 First Name
INTERVIEWER: If there is no Adult 5, enter "Not Applicable".
	__ __ __ __ __ __ __ __ __ __ __ __ __ __ __ __ __ __ __ __
If RES38 is equal to "Not Applicable", then skip to RES41.
RES39.	RELADLT5. Adult 5 Relationship to TC
INTERVIEWER: Do not read response options to participant  (Choose one)
	01	Brother
	02	Sister
	03	Cousin
	04	Biological mother
	05	Stepmother
	06	Adoptive mother
	07	Foster Mother
	08	Biological father
	09	Stepfather
	10	Adoptive father
	11	Foster father
	12	Legal guardian
	13	Grandmother
	14	Grandfather
	15	Aunt
	16	Uncle
	17	Other (Specify)
	97	Don't Know
	98	Refuse to Answer
	99	Not Applicable
If RES39 is not equal to 17, then skip to RES41.
RES40.	OTHADLT5. Specify Other Relationship to [Response to RES1]
	__ __ __ __ __ __ __ __ __ __ __ __ __ __ __ __ __ __ __ __
RES41.	JUMPBK2
Interviewer: Do you need to jump to a previous section?
If so, click YES to return to the start of the interview, where you can then choose to jump ahead to any section.
Click NO to continue.
	1	Yes
	0	No
	7	Don't Know
	8	Refuse to Answer
	9	Not Applicable
If RES41 is equal to 1 then  skip to PRE4.

SECTION CH: Child Health
These next few questions are about your child's health in the past year.
Interviewer: Section2 of 12: CH: Child Health
CH1.	SEENDOC. In the past year, has [Response to RES1] been to a doctor or other healthcare provider?
	1	Yes
	0	No
	7	Don't Know
	8	Refuse to Answer
	9	Not Applicable
If CH1 is equal to 0, then skip to CH3.
CH2.	TCHAD. In the past year, has a doctor or other healthcare provider said [Response to RES1] had:
(check all that apply)
INTERVIEWER: If none apply, click "Next Question".  (Check all that apply)  (Check all that apply)
	__	a cold
	__	an ear infection
	__	bronchitis or bronchiolitis
	__	pneumonia
	__	asthma or reactive airway disease
	__	skin condition eczema or atopic dermatitis
	__	cystic fibrosis or other pulmonary problems
	__	Don't Know
	__	Refuse to Answer
	__	Not Applicable
CH3.	TCEROOM. How many times in the past year has [Response to RES1] been treated or seen at an emergency room for coughing or difficulty breathing?
	__ __
	97	Don't Know
	98	Refuse to Answer
	99	Not Applicable
CH4.	TCOVER. How many times in the past year has [Response to RES1] been admitted as an overnight patient in a hospital for coughing or difficulty breathing?
	__ __
	97	Don't Know
	98	Refuse to Answer
	99	Not Applicable
CH5.	TCHEALTH. In general, would you say [Response to RES1]'s health is excellent, very good, good, fair or poor?  (Choose one)
	1	poor
	2	fair
	3	good
	4	very good
	5	excellent
	7	Don't Know
	8	Refuse to Answer
	9	Not Applicable
CH6.	JUMPBK3
Interviewer: Do you need to jump to a previous section?
If so, click YES to return to the start of the interview, where you can then choose to jump ahead to any section.
Click NO to continue.
	1	Yes
	0	No
	7	Don't Know
	8	Refuse to Answer
	9	Not Applicable
If CH6 is equal to 1 then  skip to PRE4.

SECTION HCV: Heating, Cooling, or Ventilation
I would like to ask you how often activities related to heating, air conditioning, or air flows in your home occurred during the past 7 days.
Interviewer: Section 3 of 12: HCV: Heating, Cooling, or Ventilation
HCV1.	WOOD7. In the past 7 days, on how many days did you or someone in your home use a wood burning stove or fireplace?
INTERVIEWER: Click "Not Applicable" if there is no such item/device in the home, to skip the next two questions.
	__ __ . __ __
	97	Don't Know
	98	Refuse to Answer
	99	Not Applicable
If HCV1 is equal to 0 or HCV1 is equal to "Not Applicable" or HCV1 is equal to "Don't Know" or HCV1 is equal to "Refuse to Answer" or HCV1 is equal to "skipped", then skip to HCV4.
HCV2.	WOODHR. About how many hours per day did you or someone use a wood burning stove or fireplace?
	__ __ . __ __
	97	Don't Know
	98	Refuse to Answer
	99	Not Applicable
HCV3.	WOOD123. Was the wood burning stove or fireplace typically on low, medium, or high?  (Choose one)
	1	low
	2	medium
	3	high
	7	Don't Know
	8	Refuse to Answer
	9	Not Applicable
HCV4.	GASH7. In the past 7 days, on how many days did you or someone in your home use a gas floor heater, space heater, or wall-mounted heater?
INTERVIEWER: Click "Not Applicable" if there is no such item/device in the home, to skip the next two questions.
	__ __ . __ __
	97	Don't Know
	98	Refuse to Answer
	99	Not Applicable
If HCV4 is equal to 0 or HCV4 is equal to "Not Applicable" or HCV4 is equal to "Don't Know" or HCV4 is equal to "Refuse to Answer" or HCV4 is equal to "skipped", then skip to HCV7.
HCV5.	GASHHR. About how many hours per day did you or someone use gas floor heater, space heater, or wall-mounted heater?
	__ __ . __ __
	97	Don't Know
	98	Refuse to Answer
	99	Not Applicable
HCV6.	GASH123. Was the gas floor heater, space heater, or wall-mounted heater typically on low, medium, or high?  (Choose one)
	1	low
	2	medium
	3	high
	7	Don't Know
	8	Refuse to Answer
	9	Not Applicable
HCV7.	CENT7. In the past 7 days, on how many days did you or someone in your home use a central air handling system (heating, cooling, or ventilation)?
INTERVIEWER: Click "Not Applicable" if there is no such item/device in the home, to skip the next two questions.
	__ __ . __ __
	97	Don't Know
	98	Refuse to Answer
	99	Not Applicable
If HCV7 is equal to 0 or HCV7 is equal to "Not Applicable" or HCV7 is equal to "Don't Know" or HCV7 is equal to "Refuse to Answer" or HCV7 is equal to "skipped", then skip to HCV10.
HCV8.	CENTHR. About how many hours per day did you or someone use a central air handling system (heating, cooling, or ventilation)?
	__ __ . __ __
	97	Don't Know
	98	Refuse to Answer
	99	Not Applicable
HCV9.	CENT123. Was the central air handling system (heating, cooling, or ventilation) typically on low, medium, or high?  (Choose one)
	1	low
	2	medium
	3	high
	7	Don't Know
	8	Refuse to Answer
	9	Not Applicable
HCV10.	PUR7. In the past 7 days, on how many days did you or someone in your home use an air purifier with a fan?
INTERVIEWER: Click "Not Applicable" if there is no such item/device in the home, to skip the next two questions.
	__ __ . __ __
	97	Don't Know
	98	Refuse to Answer
	99	Not Applicable
If HCV10 is equal to 0 or HCV10 is equal to "Not Applicable" or HCV10 is equal to "Don't Know" or HCV10 is equal to "Refuse to Answer" or HCV10 is equal to "skipped", then skip to HCV13.
HCV11.	PURHR. About how many hours per day did you or someone use an air purifier with a fan?
	__ __ . __ __
	97	Don't Know
	98	Refuse to Answer
	99	Not Applicable
HCV12.	PUR123. Was the air purifier with a fan typically on low, medium, or high?  (Choose one)
	1	low
	2	medium
	3	high
	7	Don't Know
	8	Refuse to Answer
	9	Not Applicable
HCV13.	EXH7. In the past 7 days, on how many days did you or someone in your home use an exhaust fan in the kitchen?
INTERVIEWER: Click "Not Applicable" if there is no such item/device in the home, to skip the next two questions.
	__ __ . __ __
	97	Don't Know
	98	Refuse to Answer
	99	Not Applicable
If HCV13 is equal to 0 or HCV13 is equal to "Not Applicable" or HCV13 is equal to "Don't Know" or HCV13 is equal to "Refuse to Answer" or HCV13 is equal to "skipped", then skip to HCV16.
HCV14.	EXHHR. About how many hours per day did you or someone use an exhaust fan in the kitchen?
	__ __ . __ __
	97	Don't Know
	98	Refuse to Answer
	99	Not Applicable
HCV15.	EXH123. Was the exhaust fan in the kitchen typically on low, medium, or high?  (Choose one)
	1	low
	2	medium
	3	high
	7	Don't Know
	8	Refuse to Answer
	9	Not Applicable
HCV16.	ACFAN7. In the past 7 days, on how many days did you or someone in your home use a window fan or window air conditioner?
INTERVIEWER: Click "Not Applicable" if there is no such item/device in the home, to skip the next two questions.
	__ __ . __ __
	97	Don't Know
	98	Refuse to Answer
	99	Not Applicable
If HCV16 is equal to 0 or HCV16 is equal to "Not Applicable" or HCV16 is equal to "Don't Know" or HCV16 is equal to "Refuse to Answer" or HCV16 is equal to "skipped", then skip to HCV19.
HCV17.	ACFANHR. About how many hours per day did you or someone use a window fan or window air conditioner?
	__ __ . __ __
	97	Don't Know
	98	Refuse to Answer
	99	Not Applicable
HCV18.	ACFAN123. Was the window fan or window air conditioner typically on low, medium, or high?  (Choose one)
	1	low
	2	medium
	3	high
	7	Don't Know
	8	Refuse to Answer
	9	Not Applicable
HCV19.	WIN7. In the past 7 days, on how many days did you or someone in your home open a window?
	__ __ . __ __
	97	Don't Know
	98	Refuse to Answer
	99	Not Applicable
If HCV19 is equal to 0 or HCV19 is equal to "Not Applicable" or HCV19 is equal to "Don't Know" or HCV19 is equal to "Refuse to Answer" or HCV19 is equal to "skipped", then skip to HCV21.
HCV20.	WINHR. About how many hours per day did you or someone open a window?
	__ __ . __ __
	97	Don't Know
	98	Refuse to Answer
	99	Not Applicable
HCV21.	DOOR7. In the past 7 days, on how many days did you or someone in your home open an exterior door?
INTERVIEWER: An exterior door is one leading outside the home.
	__ __ . __ __
	97	Don't Know
	98	Refuse to Answer
	99	Not Applicable
If HCV21 is equal to 0 or HCV21 is equal to "Not Applicable" or HCV21 is equal to "Don't Know" or HCV21 is equal to "Refuse to Answer" or HCV21 is equal to "skipped", then skip to HCV23.
HCV22.	DOORHR. About how many hours per day did you or someone open an exterior door?
	__ __ . __ __
	97	Don't Know
	98	Refuse to Answer
	99	Not Applicable
HCV23.	JUMPBK4
Interviewer: Do you need to jump to a previous section?
If so, click YES to return to the start of the interview, where you can then choose to jump ahead to any section.
Click NO to continue.
	1	Yes
	0	No
	7	Don't Know
	8	Refuse to Answer
	9	Not Applicable
If HCV23 is equal to 1 then  skip to PRE4.

SECTION VNT: Ventilation Behaviors
The following questions are about air flow inside your home during cooking, cleaning, or smoking activities. When you answer, please think about the ways your home was ventilated during the past 7 days.
Interviewer: Section 4 of 12: VNT: Ventilation Behaviors
If HCV19 is equal to 0 or HCV19 is equal to "Not Applicable" or HCV19 is equal to "Don't Know" or HCV19 is equal to "Refuse to Answer" or HCV19 is equal to "skipped", then skip to VNT4.
VNT1.	WINDOWE1. In the past 7 days, did you or anyone in your home open window(s) in the same room where someone was cooking?
	1	Yes
	0	No
	7	Don't Know
	8	Refuse to Answer
	9	Not Applicable
VNT2.	WINDOWE2. In the past 7 days, did you or anyone in your home open window(s) in the same room where someone was cleaning (sweeping, dusting, or vacuuming)?
	1	Yes
	0	No
	7	Don't Know
	8	Refuse to Answer
	9	Not Applicable
VNT3.	WINDOWE3. In the past 7 days, did you or any one in your home open window(s) in the same room where someone was smoking?
	1	Yes
	0	No
	7	Don't Know
	8	Refuse to Answer
	9	Not Applicable
VNT4.	STANDE3. In the past 7 days, did you or anyone who was smoking in your home stand/sit by an open door/window while they were smoking?
	1	Yes
	0	No
	7	Don't Know
	8	Refuse to Answer
	9	Not Applicable
VNT5.	INTERE1. In the past 7 days, did you or anyone in your home close an interior door connected to the room where someone was cooking?
INTERVIEWER: An interior door is one not leading outside the home.
	1	Yes
	0	No
	7	Don't Know
	8	Refuse to Answer
	9	Not Applicable
VNT6.	INTERE2. In the past 7 days, did you or anyone in your home close an interior door connected to the room where someone was cleaning (sweeping, dusting, or vacuuming)?
	1	Yes
	0	No
	7	Don't Know
	8	Refuse to Answer
	9	Not Applicable
VNT7.	INTERE3. In the past 7 days, did you or anyone in your home close an interior door connected to the room where someone was smoking?
	1	Yes
	0	No
	7	Don't Know
	8	Refuse to Answer
	9	Not Applicable
VNT8.	EXTERE1. In the past 7 days, did you or any one in your home open an exterior door while someone was cooking?
INTERVIEWER: An exterior door is one leading outside the home.
	1	Yes
	0	No
	7	Don't Know
	8	Refuse to Answer
	9	Not Applicable
VNT9.	EXTERE2. In the past 7 days, did you or any one in your home open an exterior door while someone was cleaning (sweeping, dusting, or vacuuming)?
	1	Yes
	0	No
	7	Don't Know
	8	Refuse to Answer
	9	Not Applicable
VNT10.	EXTERE3. In the past 7 days, did you or any one in your home open an exterior door while someone was smoking?
	1	Yes
	0	No
	7	Don't Know
	8	Refuse to Answer
	9	Not Applicable
If HCV10 is equal to 0 or HCV10 is equal to "Not Applicable" or HCV10 is equal to "Don't Know" or HCV10 is equal to "Refuse to Answer" or HCV10 is equal to "skipped", then skip to VNT14.
VNT11.	PURIFE1. In the past 7 days, did you or anyone in your home use an air purifier with a fan in the same room where someone was cooking?
INTERVIEWER: Click "Not Applicable" if there is no such device in the home, to skip the next two questions.
	1	Yes
	0	No
	7	Don't Know
	8	Refuse to Answer
	9	Not Applicable
If VNT11 is equal to "Not Applicable", then skip to VNT14.
VNT12.	PURIFE2. In the past 7 days, did you or anyone in your home use an air purifier with a fan in the same room where someone was cleaning (sweeping, dusting, or vacuuming)?
	1	Yes
	0	No
	7	Don't Know
	8	Refuse to Answer
	9	Not Applicable
VNT13.	PURIFE3. In the past 7 days, did you or anyone in your home use an air purifier with a fan in the same room where someone was smoking?
	1	Yes
	0	No
	7	Don't Know
	8	Refuse to Answer
	9	Not Applicable
VNT14.	EXHSTE1. In the past 7 days, did you or anyone in your home use an exhaust fan in the same room where someone was cooking?
INTERVIEWER: Click "Not Applicable" if there is no such device in the home, to skip the next two questions.
	1	Yes
	0	No
	7	Don't Know
	8	Refuse to Answer
	9	Not Applicable
If VNT14 is equal to "Not Applicable", then skip to VNT17.
VNT15.	EXHSTE2. In the past 7 days, did you or anyone in your home use an exhaust fan in the same room where someone was cleaning (sweeping, dusting, or vacuuming)?
	1	Yes
	0	No
	7	Don't Know
	8	Refuse to Answer
	9	Not Applicable
VNT16.	EXHSTE3. In the past 7 days, did you or anyone in your home use an exhaust fan in the same room where someone was smoking?
	1	Yes
	0	No
	7	Don't Know
	8	Refuse to Answer
	9	Not Applicable
VNT17.	FANE1. In the past 7 days, did you or anyone in your home use a ceiling fan or directional fan in the same room where someone was cooking?
INTERVIEWER: Click "Not Applicable" if there is no such device in the home, to skip the next two questions.
	1	Yes
	0	No
	7	Don't Know
	8	Refuse to Answer
	9	Not Applicable
If VNT17 is equal to "Not Applicable", then skip to instruction before VNT20.
VNT18.	FANE2. In the past 7 days, did you or anyone in your home use a ceiling fan or directional fan in the same room where someone was cleaning (sweeping, dusting, or vacuuming)?
	1	Yes
	0	No
	7	Don't Know
	8	Refuse to Answer
	9	Not Applicable
VNT19.	FANE3. In the past 7 days, did you or anyone in your home use a ceiling fan or directional fan in the same room where someone was smoking?
	1	Yes
	0	No
	7	Don't Know
	8	Refuse to Answer
	9	Not Applicable
If HCV16 is equal to 0 or HCV16 is equal to "Not Applicable" or HCV16 is equal to "Don't Know" or HCV16 is equal to "Refuse to Answer" or HCV16 is equal to "skipped", then skip to instruction before VNT23.
VNT20.	ACWINE1. In the past 7 days, did you or anyone in your home use a window fan or window air conditioner while someone was cooking?
INTERVIEWER: Click "Not Applicable" if there is no such device in the home, to skip the next two questions.
	1	Yes
	0	No
	7	Don't Know
	8	Refuse to Answer
	9	Not Applicable
If VNT20 is equal to "Not Applicable", then skip to instruction before VNT23.
VNT21.	ACWINE2. In the past 7 days, did you or any one in your home use a window fan or window air conditioner while someone was cleaning (sweeping, dusting, or vacuuming)?
	1	Yes
	0	No
	7	Don't Know
	8	Refuse to Answer
	9	Not Applicable
VNT22.	ACWINE3. In the past 7 days, did you or any one in your home use a window fan or window air conditioner while someone was smoking?
	1	Yes
	0	No
	7	Don't Know
	8	Refuse to Answer
	9	Not Applicable
If HCV7 is equal to 0 or HCV7 is equal to "Not Applicable" or HCV7 is equal to "Don't Know" or HCV7 is equal to "Refuse to Answer" or HCV7 is equal to "skipped", then skip to VNT26.
VNT23.	CENTE1. In the past 7 days, did you or any one in your home use a central air handling system (heating, cooling, or ventilation) while someone was cooking?
INTERVIEWER: Click "Not Applicable" if there is no such device in the home, to skip the next two questions.
	1	Yes
	0	No
	7	Don't Know
	8	Refuse to Answer
	9	Not Applicable
If VNT23 is equal to "Not Applicable", then skip to VNT26.
VNT24.	CENTE2. In the past 7 days, did you or any one in your home use a central air handling system (heating, cooling, or ventilation) while someone was cleaning (sweeping, dusting, or vacuuming)?
	1	Yes
	0	No
	7	Don't Know
	8	Refuse to Answer
	9	Not Applicable
VNT25.	CENTE3. In the past 7 days, did you or any one in your home use a central air handling system (heating, cooling, or ventilation) while someone was smoking?
	1	Yes
	0	No
	7	Don't Know
	8	Refuse to Answer
	9	Not Applicable
VNT26.	LEAVEE3. In the past 7 days, did you or anyone who was smoking in your home leave the house while they were smoking?
	1	Yes
	0	No
	7	Don't Know
	8	Refuse to Answer
	9	Not Applicable
VNT27.	JUMPBK5
Interviewer: Do you need to jump to a previous section?
If so, click YES to return to the start of the interview, where you can then choose to jump ahead to any section.
Click NO to continue.
	1	Yes
	0	No
	7	Don't Know
	8	Refuse to Answer
	9	Not Applicable
If VNT27 is equal to 1 then  skip to PRE4.

SECTION PGA: Particle Generating Activities
The next group of questions are about events in your home that may produce tiny air particles.
Interviewer: Section 5 of 12: PGA: Particle Generating Activities
PGA1.	INCEN7. In the past 7 days, how many days did you or someone in your home burn incense or candles?
INTERVIEWER: Click "Not Applicable" if there is no such item/activity in the home, to skip the next 5 questions.
	__ __ . __ __
	97	Don't Know
	98	Refuse to Answer
	99	Not Applicable
If PGA1 is equal to 0 or PGA1 is equal to "Not Applicable" or PGA1 is equal to "Refuse to Answer" or PGA1 is equal to "Don't Know" or PGA1 is equal to "skipped", then skip to PGA7.
PGA2.	INCENWH. Where did someone burn incense or candles? 
(Check all that apply)
INTERVIEWER: Use Floor Plan Sketch to prompt respondent.  (Check all that apply)
	__	LIV = Living Room
	__	KIT = Kitchen
	__	DIN = Dining Room
	__	BED1 = 1st Bedroom
	__	BED2 = 2nd Bedroom
	__	BED3 = 3rd Bedroom
	__	BED4 = 4th Bedroom
	__	BATH1 = 1st Bathroom
	__	BATH2 = 2nd Bathroom
	__	BATH3 = 3rd Bathroom
	__	BAL1 = Balcony1
	__	BAL2 = Balcony2
	__	PAT = Patio
	__	LOB = Lobby
	__	HALL1 = Hallway 1
	__	HALL2 = Hallway 2
	__	HALL3 = Hallway 3
	__	LAUN = Laundry/Utility
	__	DEN = Rec/Family Rm
	__	OTH1 = Other Room1
	__	OTH2 = Other Room2
	__	OTH3 = Other Room3
	__	Don't Know
	__	Refuse to Answer
	__	Not Applicable
PGA3.	INCENM. On a typical day in the past 7 days, how many times did someone burn incense or candles in your home during the Morning (6 AM to Noon)?
	__ __ . __ __
	97	Don't Know
	98	Refuse to Answer
	99	Not Applicable
PGA4.	INCENA. On a typical day in the past 7 days, how many times did someone burn incense or candles in your home during the Afternoon (Noon to 6PM)?
	__ __ . __ __
	97	Don't Know
	98	Refuse to Answer
	99	Not Applicable
PGA5.	INCENE. On a typical day in the past 7 days, how many times did someone burn incense or candles in your home during the Evening (6PM to Midnight)?
	__ __ . __ __
	97	Don't Know
	98	Refuse to Answer
	99	Not Applicable
PGA6.	INCENN. On a typical day in the past 7 days, how many times did someone burn incense or candles in your home during the Night (Midnight to 6AM)?
	__ __ . __ __
	97	Don't Know
	98	Refuse to Answer
	99	Not Applicable
PGA7.	FOOD7. In the past 7 days, how many days did you or someone in your home burn food?
INTERVIEWER: Click "Not Applicable" if there is no such item/activity in the home, to skip the next 5 questions.
	__ __ . __ __
	97	Don't Know
	98	Refuse to Answer
	99	Not Applicable
If PGA7 is equal to 0 or PGA7 is equal to "Not Applicable" or PGA7 is equal to "Refuse to Answer" or PGA7 is equal to "Don't Know" or PGA7 is equal to "skipped", then skip to PGA13.
PGA8.	FOODWH. Where did someone burn food? 
(Check all that apply)
INTERVIEWER: Use Floor Plan Sketch to prompt respondent.  (Check all that apply)
	__	LIV = Living Room
	__	KIT = Kitchen
	__	DIN = Dining Room
	__	BED1 = 1st Bedroom
	__	BED2 = 2nd Bedroom
	__	BED3 = 3rd Bedroom
	__	BED4 = 4th Bedroom
	__	BATH1 = 1st Bathroom
	__	BATH2 = 2nd Bathroom
	__	BATH3 = 3rd Bathroom
	__	BAL1 = Balcony1
	__	BAL2 = Balcony2
	__	PAT = Patio
	__	LOB = Lobby
	__	HALL1 = Hallway 1
	__	HALL2 = Hallway 2
	__	HALL3 = Hallway 3
	__	LAUN = Laundry/Utility
	__	DEN = Rec/Family Rm
	__	OTH1 = Other Room1
	__	OTH2 = Other Room2
	__	OTH3 = Other Room3
	__	Don't Know
	__	Refuse to Answer
	__	Not Applicable
PGA9.	FOODM. On a typical day in the past 7 days, how many times did someone burn food in your home during the Morning (6AM to Noon)?
	__ __ . __ __
	97	Don't Know
	98	Refuse to Answer
	99	Not Applicable
PGA10.	FOODA. On a typical day in the past 7 days, how many times did someone burn food in your home during the Afternoon (Noon to 6PM)?
	__ __ . __ __
	97	Don't Know
	98	Refuse to Answer
	99	Not Applicable
PGA11.	FOODE. On a typical day in the past 7 days, how many times did someone burn food in your home during the Evening (6PM to Midnight)?
	__ __ . __ __
	97	Don't Know
	98	Refuse to Answer
	99	Not Applicable
PGA12.	FOODN. On a typical day in the past 7 days, how many times did someone burn food in your home during the Night (Midnight to 6AM)?
	__ __ . __ __
	97	Don't Know
	98	Refuse to Answer
	99	Not Applicable
PGA13.	FRY7. In the past 7 days, how many days did you or someone in your home fry or sauté food with oil or fat?
INTERVIEWER: This includes vegetable oil, olive oil, lard, butter, margarine, "I Can't Believe It's Not Butter!", bacon grease, PAM
Click "Not Applicable" if there is no such item/activity in the home, to skip the next 5 questions.
	__ __ . __ __
	97	Don't Know
	98	Refuse to Answer
	99	Not Applicable
If PGA13 is equal to 0 or PGA13 is equal to "Not Applicable" or PGA13 is equal to "Refuse to Answer" or PGA13 is equal to "Don't Know" or PGA13 is equal to "skipped", then skip to PGA19.
PGA14.	FRYWH. Where did someone fry or sauté food with oil or fat?
(Check all that apply)
INTERVIEWER: Use Floor Plan Sketch to prompt respondent.  (Check all that apply)
	__	LIV = Living Room
	__	KIT = Kitchen
	__	DIN = Dining Room
	__	BED1 = 1st Bedroom
	__	BED2 = 2nd Bedroom
	__	BED3 = 3rd Bedroom
	__	BED4 = 4th Bedroom
	__	BATH1 = 1st Bathroom
	__	BATH2 = 2nd Bathroom
	__	BATH3 = 3rd Bathroom
	__	BAL1 = Balcony1
	__	BAL2 = Balcony2
	__	PAT = Patio
	__	LOB = Lobby
	__	HALL1 = Hallway 1
	__	HALL2 = Hallway 2
	__	HALL3 = Hallway 3
	__	LAUN = Laundry/Utility
	__	DEN = Rec/Family Rm
	__	OTH1 = Other Room1
	__	OTH2 = Other Room2
	__	OTH3 = Other Room3
	__	Don't Know
	__	Refuse to Answer
	__	Not Applicable
PGA15.	FRYM. On a typical day in the past 7 days, how many times did someone fry or sauté food with oil or fat in your home during the Morning (6 AM to Noon)?
	__ __ . __ __
	97	Don't Know
	98	Refuse to Answer
	99	Not Applicable
PGA16.	FRYA. On a typical day in the past 7 days, how many times did someone fry or sauté food with oil or fat in your home during the Afternoon (Noon to 6PM)?
	__ __ . __ __
	97	Don't Know
	98	Refuse to Answer
	99	Not Applicable
PGA17.	FRYE. On a typical day in the past 7 days, how many times did someone fry or sauté food with oil or fat in your home during the Evening (6PM to Midnight)?
	__ __ . __ __
	97	Don't Know
	98	Refuse to Answer
	99	Not Applicable
PGA18.	FRYN. On a typical day in the past 7 days, how many times did someone fry or sauté food with oil or fat in your home during the Night (Midnight to 6AM)?
	__ __ . __ __
	97	Don't Know
	98	Refuse to Answer
	99	Not Applicable
PGA19.	GAS7. In the past 7 days, how many days did you or someone in your home use a gas/propane appliance to cook or heat food (e.g., stove, portable cooktop, grill, or toaster over)?
INTERVIEWER: Click "Not Applicable" if there is no such item/device in the home, to skip the next 5 questions.
	__ __ . __ __
	97	Don't Know
	98	Refuse to Answer
	99	Not Applicable
If PGA19 is equal to 0 or PGA19 is equal to "Not Applicable" or PGA19 is equal to "Refuse to Answer" or PGA19 is equal to "Don't Know" or PGA19 is equal to "skipped", then skip to PGA25.
PGA20.	GASWH. Where did someone use a gas/propane appliance to cook or heat food (e.g., stove, portable cooktop, grill, or toaster over)? 
(Check all that apply)
INTERVIEWER: Use Floor Plan Sketch to prompt respondent.  (Check all that apply)
	__	LIV = Living Room
	__	KIT = Kitchen
	__	DIN = Dining Room
	__	BED1 = 1st Bedroom
	__	BED2 = 2nd Bedroom
	__	BED3 = 3rd Bedroom
	__	BED4 = 4th Bedroom
	__	BATH1 = 1st Bathroom
	__	BATH2 = 2nd Bathroom
	__	BATH3 = 3rd Bathroom
	__	BAL1 = Balcony1
	__	BAL2 = Balcony2
	__	PAT = Patio
	__	LOB = Lobby
	__	HALL1 = Hallway 1
	__	HALL2 = Hallway 2
	__	HALL3 = Hallway 3
	__	LAUN = Laundry/Utility
	__	DEN = Rec/Family Rm
	__	OTH1 = Other Room1
	__	OTH2 = Other Room2
	__	OTH3 = Other Room3
	__	Don't Know
	__	Refuse to Answer
	__	Not Applicable
PGA21.	GASM. On a typical day in the past 7 days, how many times did someone use a gas/propane appliance to cook or heat food (e.g., stove, portable cooktop, grill, or toaster over) in your home during the Morning (6 AM to Noon)?
	__ __ . __ __
	97	Don't Know
	98	Refuse to Answer
	99	Not Applicable
PGA22.	GASA. On a typical day in the past 7 days, how many times did someone use a gas/propane appliance to cook or heat food (e.g., stove, portable cooktop, grill, or toaster over) in your home during the Afternoon (Noon to 6PM)?
	__ __ . __ __
	97	Don't Know
	98	Refuse to Answer
	99	Not Applicable
PGA23.	GASE. On a typical day in the past 7 days, how many times did someone use a gas/propane appliance to cook or heat food (e.g., stove, portable cooktop, grill, or toaster over) in your home during the Evening (6PM to Midnight)?
	__ __ . __ __
	97	Don't Know
	98	Refuse to Answer
	99	Not Applicable
PGA24.	GASN. On a typical day in the past 7 days, how many times did someone use a gas/propane appliance to cook or heat food (e.g., stove, portable cooktop, grill, or toaster over) in your home during the Night (Midnight to 6AM)?
	__ __ . __ __
	97	Don't Know
	98	Refuse to Answer
	99	Not Applicable
PGA25.	ELEC7. In the past 7 days, how many days did you or someone in your home use an electric appliance to cook or heat food (e.g., stove, or portable cooktop, microwave, grill or toaster oven)?
INTERVIEWER: Click "Not Applicable" if there is no such item/device in the home, to skip the next 5 questions.
	__ __ . __ __
	97	Don't Know
	98	Refuse to Answer
	99	Not Applicable
If PGA25 is equal to 0 or PGA25 is equal to "Not Applicable" or PGA25 is equal to "Refuse to Answer" or PGA25 is equal to "Don't Know" or PGA25 is equal to "skipped", then skip to PGA31.
PGA26.	ELECWH. Where did someone use an electric appliance to cook or heat food (e.g., stove, or portable cooktop, microwave, grill or toaster oven)? 
(Check all that apply)
INTERVIEWER: Use Floor Plan Sketch to prompt respondent.  (Check all that apply)
	__	LIV = Living Room
	__	KIT = Kitchen
	__	DIN = Dining Room
	__	BED1 = 1st Bedroom
	__	BED2 = 2nd Bedroom
	__	BED3 = 3rd Bedroom
	__	BED4 = 4th Bedroom
	__	BATH1 = 1st Bathroom
	__	BATH2 = 2nd Bathroom
	__	BATH3 = 3rd Bathroom
	__	BAL1 = Balcony1
	__	BAL2 = Balcony2
	__	PAT = Patio
	__	LOB = Lobby
	__	HALL1 = Hallway 1
	__	HALL2 = Hallway 2
	__	HALL3 = Hallway 3
	__	LAUN = Laundry/Utility
	__	DEN = Rec/Family Rm
	__	OTH1 = Other Room1
	__	OTH2 = Other Room2
	__	OTH3 = Other Room3
	__	Don't Know
	__	Refuse to Answer
	__	Not Applicable
PGA27.	ELECM. On a typical day in the past 7 days, how many times did someone use an electric appliance to cook or heat food (e.g., stove, or portable cooktop, microwave, grill or toaster oven) in your home during the Morning (6 AM to Noon)?
	__ __ . __ __
	97	Don't Know
	98	Refuse to Answer
	99	Not Applicable
PGA28.	ELECA. On a typical day in the past 7 days, how many times did someone use an electric appliance to cook or heat food (e.g., stove, or portable cooktop, microwave, grill or toaster oven) in your home during the Afternoon (Noon to 6PM)?
	__ __ . __ __
	97	Don't Know
	98	Refuse to Answer
	99	Not Applicable
PGA29.	ELECE. On a typical day in the past 7 days, how many times did someone use an electric appliance to cook or heat food (e.g., stove, or portable cooktop, microwave, grill or toaster oven) in your home during the Evening (6PM to Midnight)?
	__ __ . __ __
	97	Don't Know
	98	Refuse to Answer
	99	Not Applicable
PGA30.	ELECN. On a typical day in the past 7 days, how many times did someone use an electric appliance to cook or heat food (e.g., stove, or portable cooktop, microwave, grill or toaster oven) in your home during the Night (Midnight to 6AM)?
	__ __ . __ __
	97	Don't Know
	98	Refuse to Answer
	99	Not Applicable
PGA31.	AERO7. In the past 7 days, how many days did you or someone in your home use aerosol spray products (e.g., hair spray, bug spray, deodorant)?
INTERVIEWER: Click "Not Applicable" if there is no such item in the home, to skip the next 5 questions.
	__ __ . __ __
	97	Don't Know
	98	Refuse to Answer
	99	Not Applicable
If PGA31 is equal to 0 or PGA31 is equal to "Not Applicable" or PGA31 is equal to "Refuse to Answer" or PGA31 is equal to "Don't Know" or PGA31 is equal to "skipped", then skip to PGA37.
PGA32.	AEROWH. Where did someone use aerosol spray products (e.g., hair spray, bug spray, deodorant)?
(Check all that apply)
INTERVIEWER: Use Floor Plan Sketch to prompt respondent.  (Check all that apply)
	__	LIV = Living Room
	__	KIT = Kitchen
	__	DIN = Dining Room
	__	BED1 = 1st Bedroom
	__	BED2 = 2nd Bedroom
	__	BED3 = 3rd Bedroom
	__	BED4 = 4th Bedroom
	__	BATH1 = 1st Bathroom
	__	BATH2 = 2nd Bathroom
	__	BATH3 = 3rd Bathroom
	__	BAL1 = Balcony1
	__	BAL2 = Balcony2
	__	PAT = Patio
	__	LOB = Lobby
	__	HALL1 = Hallway 1
	__	HALL2 = Hallway 2
	__	HALL3 = Hallway 3
	__	LAUN = Laundry/Utility
	__	DEN = Rec/Family Rm
	__	OTH1 = Other Room1
	__	OTH2 = Other Room2
	__	OTH3 = Other Room3
	__	Don't Know
	__	Refuse to Answer
	__	Not Applicable
PGA33.	AEROM. On a typical day in the past 7 days, how many times did someone use aerosol spray products (e.g., hair spray, bug spray, deodorant) in your home during the Morning (6 AM to Noon)?
	__ __ . __ __
	97	Don't Know
	98	Refuse to Answer
	99	Not Applicable
PGA34.	AEROA. On a typical day in the past 7 days, how many times did someone use aerosol spray products (e.g., hair spray, bug spray, deodorant) in your home during the Afternoon (Noon to 6PM)?
	__ __ . __ __
	97	Don't Know
	98	Refuse to Answer
	99	Not Applicable
PGA35.	AEROE. On a typical day in the past 7 days, how many times did someone use aerosol spray products (e.g., hair spray, bug spray, deodorant) in your home during the Evening (6PM to Midnight)?
	__ __ . __ __
	97	Don't Know
	98	Refuse to Answer
	99	Not Applicable
PGA36.	AERON. On a typical day in the past 7 days, how many times did someone use aerosol spray products (e.g., hair spray, bug spray, deodorant) in your home during the Night (Midnight to 6AM)?
	__ __ . __ __
	97	Don't Know
	98	Refuse to Answer
	99	Not Applicable
PGA37.	DUST7. In the past 7 days, how many days did you or someone in your home vacuum/dust/sweep?
INTERVIEWER: Click "Not Applicable" if there is no such item/activity in the home, to skip the next 5 questions.
	__ __ . __ __
	97	Don't Know
	98	Refuse to Answer
	99	Not Applicable
If PGA37 is equal to 0 or PGA37 is equal to "Not Applicable" or PGA37 is equal to "Refuse to Answer" or PGA37 is equal to "Don't Know" or PGA37 is equal to "skipped", then skip to PGA43.
PGA38.	DUSTWH. Where did someone vacuum/dust/sweep? 
(Check all that apply)
INTERVIEWER: Use Floor Plan Sketch to prompt respondent.  (Check all that apply)
	__	LIV = Living Room
	__	KIT = Kitchen
	__	DIN = Dining Room
	__	BED1 = 1st Bedroom
	__	BED2 = 2nd Bedroom
	__	BED3 = 3rd Bedroom
	__	BED4 = 4th Bedroom
	__	BATH1 = 1st Bathroom
	__	BATH2 = 2nd Bathroom
	__	BATH3 = 3rd Bathroom
	__	BAL1 = Balcony1
	__	BAL2 = Balcony2
	__	PAT = Patio
	__	LOB = Lobby
	__	HALL1 = Hallway 1
	__	HALL2 = Hallway 2
	__	HALL3 = Hallway 3
	__	LAUN = Laundry/Utility
	__	DEN = Rec/Family Rm
	__	OTH1 = Other Room1
	__	OTH2 = Other Room2
	__	OTH3 = Other Room3
	__	Don't Know
	__	Refuse to Answer
	__	Not Applicable
PGA39.	DUSTM. On a typical day in the past 7 days, how many times did someone vacuum/dust/sweep in your home during the Morning (6 AM to Noon)?
	__ __ . __ __
	97	Don't Know
	98	Refuse to Answer
	99	Not Applicable
PGA40.	DUSTA. On a typical day in the past 7 days, how many times did someone vacuum/dust/sweep in your home during the Afternoon (Noon to 6PM)?
	__ __ . __ __
	97	Don't Know
	98	Refuse to Answer
	99	Not Applicable
PGA41.	DUSTE. On a typical day in the past 7 days, how many times did someone vacuum/dust/sweep in your home during the Evening (6PM to Midnight)?
	__ __ . __ __
	97	Don't Know
	98	Refuse to Answer
	99	Not Applicable
PGA42.	DUSTN. On a typical day in the past 7 days, how many times did someone vacuum/dust/sweep in your home during the Night (Midnight to 6AM)?
	__ __ . __ __
	97	Don't Know
	98	Refuse to Answer
	99	Not Applicable
PGA43.	APGA7. In the past 7 days, how many days did you or someone in your home do anything else that generates smoke, dust or particles? (specify)?
INTERVIEWER: Click "Not Applicable" if there is no such activity in the home, to skip the next 5 questions.
Examples to prompt the respondent:
·renovating (drywall, demolition, painting) producing dust, fumes, etc.
·started fire in fireplace or wood stove
·noticed pollen/spores coming inside
·sanding or wood work producing dust
·anything producing vapor, steam, droplets
·welding or work producing smoke, dust
	__ __ . __ __
	97	Don't Know
	98	Refuse to Answer
	99	Not Applicable
If PGA43 is equal to 0 or PGA43 is equal to "Not Applicable" or PGA43 is equal to "Refuse to Answer" or PGA43 is equal to "Don't Know" or PGA43 is equal to "skipped", then skip to PGA57.
PGA44.	APGA7SP. Specify Other particle generating item/activity
	__ __ __ __ __ __ __ __ __ __ __ __ __ __ __ __ __ __ __ __
PGA45.	APGAWH. Where did someone do anything else that generates smoke, dust or particles?
(Check all that apply)
INTERVIEWER: Use Floor Plan Sketch to prompt respondent.  (Check all that apply)
	__	LIV = Living Room
	__	KIT = Kitchen
	__	DIN = Dining Room
	__	BED1 = 1st Bedroom
	__	BED2 = 2nd Bedroom
	__	BED3 = 3rd Bedroom
	__	BED4 = 4th Bedroom
	__	BATH1 = 1st Bathroom
	__	BATH2 = 2nd Bathroom
	__	BATH3 = 3rd Bathroom
	__	BAL1 = Balcony1
	__	BAL2 = Balcony2
	__	PAT = Patio
	__	LOB = Lobby
	__	HALL1 = Hallway 1
	__	HALL2 = Hallway 2
	__	HALL3 = Hallway 3
	__	LAUN = Laundry/Utility
	__	DEN = Rec/Family Rm
	__	OTH1 = Other Room1
	__	OTH2 = Other Room2
	__	OTH3 = Other Room3
	__	Don't Know
	__	Refuse to Answer
	__	Not Applicable
PGA46.	APGAM. On a typical day in the past 7 days, how many times did someone do anything else that generates smoke, dust or particles in your home during the Morning (6 AM to Noon)?
	__ __ . __ __
	97	Don't Know
	98	Refuse to Answer
	99	Not Applicable
PGA47.	APGAA. On a typical day in the past 7 days, how many times did someone do anything else that generates smoke, dust or particles  in your home during the Afternoon (Noon to 6PM)?
	__ __ . __ __
	97	Don't Know
	98	Refuse to Answer
	99	Not Applicable
PGA48.	APGAE. On a typical day in the past 7 days, how many times did someone do anything else that generates smoke, dust or particles  in your home during the Evening (6PM to Midnight)?
	__ __ . __ __
	97	Don't Know
	98	Refuse to Answer
	99	Not Applicable
PGA49.	APGAN. On a typical day in the past 7 days, how many times did someone do anything else that generates smoke, dust or particles in your home during the Night (Midnight to 6AM)?
	__ __ . __ __
	97	Don't Know
	98	Refuse to Answer
	99	Not Applicable
PGA50.	BPGA7. In the past 7 days, how many days did you or someone in your home do anything else that generates smoke, dust or particles?
INTERVIEWER: Click "Not Applicable" if there is no such activity in the home, to skip the next 5 questions.
Examples to prompt the respondent:
·renovating (drywall, demolition, painting) producing dust, fumes, etc.
·started fire in fireplace or wood stove
·noticed pollen/spores coming inside
·sanding or wood work producing dust
·anything producing vapor, steam, droplets
·welding or work producing smoke, dust
	__ __ . __ __
	97	Don't Know
	98	Refuse to Answer
	99	Not Applicable
If PGA50 is equal to 0 or PGA50 is equal to "Not Applicable" or PGA50 is equal to "Refuse to Answer" or PGA50 is equal to "Don't Know" or PGA50 is equal to "skipped", then skip to PGA57.
PGA51.	BPGA7SP. Specify Other particle generating item/activity
	__ __ __ __ __ __ __ __ __ __ __ __ __ __ __ __ __ __ __ __
PGA52.	BPGAWH. Where did someone do anything else that generates smoke, dust or particles?
(Check all that apply)
INTERVIEWER: Use Floor Plan Sketch to prompt respondent.  (Check all that apply)
	__	LIV = Living Room
	__	KIT = Kitchen
	__	DIN = Dining Room
	__	BED1 = 1st Bedroom
	__	BED2 = 2nd Bedroom
	__	BED3 = 3rd Bedroom
	__	BED4 = 4th Bedroom
	__	BATH1 = 1st Bathroom
	__	BATH2 = 2nd Bathroom
	__	BATH3 = 3rd Bathroom
	__	BAL1 = Balcony1
	__	BAL2 = Balcony2
	__	PAT = Patio
	__	LOB = Lobby
	__	HALL1 = Hallway 1
	__	HALL2 = Hallway 2
	__	HALL3 = Hallway 3
	__	LAUN = Laundry/Utility
	__	DEN = Rec/Family Rm
	__	OTH1 = Other Room1
	__	OTH2 = Other Room2
	__	OTH3 = Other Room3
	__	Don't Know
	__	Refuse to Answer
	__	Not Applicable
PGA53.	BPGAM. On a typical day in the past 7 days, how many times did someone do anything else that generates smoke, dust or particles in your home during the Morning (6 AM to Noon)?
	__ __ . __ __
	97	Don't Know
	98	Refuse to Answer
	99	Not Applicable
PGA54.	BPGAA. On a typical day in the past 7 days, how many times did someone do anything else that generates smoke, dust or particles in your home during the Afternoon (Noon to 6PM)?
	__ __ . __ __
	97	Don't Know
	98	Refuse to Answer
	99	Not Applicable
PGA55.	BPGAE. On a typical day in the past 7 days, how many times did someone do anything else that generates smoke, dust or particles in your home during the Evening (6PM to Midnight)?
	__ __ . __ __
	97	Don't Know
	98	Refuse to Answer
	99	Not Applicable
PGA56.	BPGAN. On a typical day in the past 7 days, how many times did someone do anything else that generates smoke, dust or particles in your home during the Night (Midnight to 6AM)?
	__ __ . __ __
	97	Don't Know
	98	Refuse to Answer
	99	Not Applicable
PGA57.	JUMPBK6
Interviewer: Do you need to jump to a previous section?
If so, click YES to return to the start of the interview, where you can then choose to jump ahead to any section.
Click NO to continue.
	1	Yes
	0	No
	7	Don't Know
	8	Refuse to Answer
	9	Not Applicable
If PGA57 is equal to 1 then  skip to PRE4.

SECTION RTU: Residents' Tobacco Use
Now I'd like to ask you some questions about tobacco smoking by people who live in your home. Answer as best you can for others, or tell me if you don't know.
Interviewer: Section 6 of 12: RTU: Residents' Tobacco Use
RTU1.	TYPEC1. Which types of tobacco has [Response to RES1] ever smoked, even a puff? 
(mark all that apply)  (Check all that apply)
	__	cigarette
	__	cigar
	__	pipe
	__	waterpipe/hookah
	__	other (specify)
	__	none
	__	Don't Know
	__	Refuse to Answer
	__	Not Applicable
If RTU1F is equal to 1, then skip to instruction before RTU7.
If RTU1E is not equal to 1, then skip to RTU3.
RTU2.	TYPEC1S. Please specify other type of tobacco smoked ever
	__ __ __ __ __ __ __ __ __ __ __ __ __ __ __ __ __ __ __ __
RTU3.	TYP30C1. During the past 30 days which of the following types of tobacco has [Response to RES1] smoked? 
(mark all that apply)  (Check all that apply)
	__	cigarette
	__	cigar
	__	pipe
	__	waterpipe/hookah
	__	other (specify)
	__	none
	__	Don't Know
	__	Refuse to Answer
	__	Not Applicable
If RTU3E is not equal to 1, then skip to instruction before RTU5.
RTU4.	TYP30C1S. Please specify other type of tobacco smoked during the past 30 days
	__ __ __ __ __ __ __ __ __ __ __ __ __ __ __ __ __ __ __ __
If RTU1A is not equal to 1, then skip to instruction before RTU6.
RTU5.	LIFEC1. Has [Response to RES1] smoked more than 100 cigarettes in their lifetime?
	1	Yes
	0	No
	7	Don't Know
	8	Refuse to Answer
	9	Not Applicable
If RTU3A is not equal to 1, then skip to instruction before RTU7.
RTU6.	SMKNOWC1. Does [Response to RES1] currently smoke cigarettes not at all, some days, or every day?  (Choose one)
	0	Not at All
	1	Some Days
	2	Every Day
	7	Don't Know
	8	Refuse to Answer
	9	Not Applicable
If RES4 is equal to "Not Applicable" or RES4 is equal to "skipped" or RES4 is equal to "Refuse to Answer" or RES4 is equal to "Don't Know", then skip to RTU31.
RTU7.	TYPEC2. Which types of tobacco has [Response to RES4] ever smoked, even a puff? 
(mark all that apply)  (Check all that apply)
	__	cigarette
	__	cigar
	__	pipe
	__	waterpipe/hookah
	__	other (specify)
	__	none
	__	Don't Know
	__	Refuse to Answer
	__	Not Applicable
If RTU7F is equal to 1, then skip to instruction before RTU13.
If RTU7E is not equal to 1, then skip to RTU9.
RTU8.	TYPEC2S. Please specify other type of tobacco smoked ever
	__ __ __ __ __ __ __ __ __ __ __ __ __ __ __ __ __ __ __ __
RTU9.	TYP30C2. During the past 30 days which of the following types of tobacco has [Response to RES4] smoked? 
(mark all that apply)  (Check all that apply)
	__	cigarette
	__	cigar
	__	pipe
	__	waterpipe/hookah
	__	other (specify)
	__	none
	__	Don't Know
	__	Refuse to Answer
	__	Not Applicable
If RTU9E is not equal to 1, then skip to instruction before RTU11.
RTU10.	TYP30C2S. Please specify other type of tobacco smoked during the past 30 days
	__ __ __ __ __ __ __ __ __ __ __ __ __ __ __ __ __ __ __ __
If RTU7A is not equal to 1, then skip to instruction before RTU12.
RTU11.	LIFEC2. Has [Response to RES4] smoked more than 100 cigarettes in their lifetime?
	1	Yes
	0	No
	7	Don't Know
	8	Refuse to Answer
	9	Not Applicable
If RTU9A is not equal to 1, then skip to instruction before RTU13.
RTU12.	SMKNOWC2. Does [Response to RES4] currently smoke cigarettes not at all, some days, or every day?  (Choose one)
	0	Not at All
	1	Some Days
	2	Every Day
	7	Don't Know
	8	Refuse to Answer
	9	Not Applicable
If RES9 is equal to "Not Applicable" or RES9 is equal to "skipped" or RES9 is equal to "Refuse to Answer" or RES9 is equal to "Don't Know", then skip to RTU31.
RTU13.	TYPEC3. Which types of tobacco has [Response to RES9] ever smoked, even a puff? 
(mark all that apply)  (Check all that apply)
	__	cigarette
	__	cigar
	__	pipe
	__	waterpipe/hookah
	__	other (specify)
	__	none
	__	Don't Know
	__	Refuse to Answer
	__	Not Applicable
If RTU13F is equal to 1, then skip to instruction before RTU19.
If RTU13E is not equal to 1, then skip to RTU15.
RTU14.	TYPEC3S. Please specify other type of tobacco smoked ever
	__ __ __ __ __ __ __ __ __ __ __ __ __ __ __ __ __ __ __ __
RTU15.	TYP30C3. During the past 30 days which of the following types of tobacco has [Response to RES9] smoked? 
(mark all that apply)  (Check all that apply)
	__	cigarette
	__	cigar
	__	pipe
	__	waterpipe/hookah
	__	other (specify)
	__	none
	__	Don't Know
	__	Refuse to Answer
	__	Not Applicable
If RTU15E is not equal to 1, then skip to instruction before RTU17.
RTU16.	TYP30C3S. Please specify other type of tobacco smoked during the past 30 days
	__ __ __ __ __ __ __ __ __ __ __ __ __ __ __ __ __ __ __ __
If RTU13A is not equal to 1, then skip to instruction before RTU18.
RTU17.	LIFEC3. Has [Response to RES9] smoked more than 100 cigarettes in their lifetime?
	1	Yes
	0	No
	7	Don't Know
	8	Refuse to Answer
	9	Not Applicable
If RTU15A is not equal to 1, then skip to instruction before RTU19.
RTU18.	SMKNOWC3. Does [Response to RES9] currently smoke cigarettes not at all, some days, or every day?  (Choose one)
	0	Not at All
	1	Some Days
	2	Every Day
	7	Don't Know
	8	Refuse to Answer
	9	Not Applicable
If RES14 is equal to "Not Applicable" or RES14 is equal to "skipped" or RES14 is equal to "Refuse to Answer" or RES14 is equal to "Don't Know", then skip to RTU31.
RTU19.	TYPEC4. Which types of tobacco has [Response to RES14] ever smoked, even a puff? 
(mark all that apply)  (Check all that apply)
	__	cigarette
	__	cigar
	__	pipe
	__	waterpipe/hookah
	__	other (specify)
	__	none
	__	Don't Know
	__	Refuse to Answer
	__	Not Applicable
If RTU19F is equal to 1, then skip to instruction before RTU25.
If RTU19E is not equal to 1, then skip to RTU21.
RTU20.	TYPEC4S. Please specify other type of tobacco smoked ever
	__ __ __ __ __ __ __ __ __ __ __ __ __ __ __ __ __ __ __ __
RTU21.	TYP30C4. During the past 30 days which of the following types of tobacco has [Response to RES14] smoked? 
(mark all that apply)  (Check all that apply)
	__	cigarette
	__	cigar
	__	pipe
	__	waterpipe/hookah
	__	other (specify)
	__	none
	__	Don't Know
	__	Refuse to Answer
	__	Not Applicable
If RTU21E is not equal to 1, then skip to instruction before RTU23.
RTU22.	TYP30C4S. Please specify other type of tobacco smoked during the past 30 days
	__ __ __ __ __ __ __ __ __ __ __ __ __ __ __ __ __ __ __ __
If RTU19A is not equal to 1, then skip to instruction before RTU24.
RTU23.	LIFEC4. Has [Response to RES14] smoked more than 100 cigarettes in their lifetime?
	1	Yes
	0	No
	7	Don't Know
	8	Refuse to Answer
	9	Not Applicable
If RTU21A is not equal to 1, then skip to instruction before RTU25.
RTU24.	SMKNOWC4. Does [Response to RES14] currently smoke cigarettes not at all, some days, or every day?  (Choose one)
	0	Not at All
	1	Some Days
	2	Every Day
	7	Don't Know
	8	Refuse to Answer
	9	Not Applicable
If RES19 is equal to "Not Applicable" or RES19 is equal to "skipped" or RES19 is equal to "Refuse to Answer" or RES19 is equal to "Don't Know", then skip to RTU31.
RTU25.	TYPEC5. Which types of tobacco has [Response to RES19] ever smoked, even a puff? 
(mark all that apply)  (Check all that apply)
	__	cigarette
	__	cigar
	__	pipe
	__	waterpipe/hookah
	__	other (specify)
	__	none
	__	Don't Know
	__	Refuse to Answer
	__	Not Applicable
If RTU25F is equal to 1, then skip to RTU31.
If RTU25E is not equal to 1, then skip to RTU27.
RTU26.	TYPEC5S. Please specify other type of tobacco smoked ever
	__ __ __ __ __ __ __ __ __ __ __ __ __ __ __ __ __ __ __ __
RTU27.	TYP30C5. During the past 30 days which of the following types of tobacco has [Response to RES19] smoked? 
(mark all that apply)  (Check all that apply)
	__	cigarette
	__	cigar
	__	pipe
	__	waterpipe/hookah
	__	other (specify)
	__	none
	__	Don't Know
	__	Refuse to Answer
	__	Not Applicable
If RTU27E is not equal to 1, then skip to instruction before RTU29.
RTU28.	TYP30C5S. Please specify other type of tobacco smoked during the past 30 days
	__ __ __ __ __ __ __ __ __ __ __ __ __ __ __ __ __ __ __ __
If RTU25A is not equal to 1, then skip to instruction before RTU30.
RTU29.	LIFEC5. Has [Response to RES19] smoked more than 100 cigarettes in their lifetime?
	1	Yes
	0	No
	7	Don't Know
	8	Refuse to Answer
	9	Not Applicable
If RTU27A is not equal to 1, then skip to RTU31.
RTU30.	SMKNOWC5. Does [Response to RES19] currently smoke cigarettes not at all, some days, or every day?  (Choose one)
	0	Not at All
	1	Some Days
	2	Every Day
	7	Don't Know
	8	Refuse to Answer
	9	Not Applicable
RTU31.	TYPEA1. Which types of tobacco has [Response to RES24] ever smoked, even a puff? 
(mark all that apply)  (Check all that apply)
	__	cigarette
	__	cigar
	__	pipe
	__	waterpipe/hookah
	__	other (specify)
	__	none
	__	Don't Know
	__	Refuse to Answer
	__	Not Applicable
If RTU31F is equal to 1, then skip to instruction before RTU37.
If RTU31E is not equal to 1, then skip to RTU33.
RTU32.	TYPEA1S. Please specify other type of tobacco smoked ever
	__ __ __ __ __ __ __ __ __ __ __ __ __ __ __ __ __ __ __ __
RTU33.	TYP30A1. During the past 30 days which of the following types of tobacco has [Response to RES24] smoked? 
(mark all that apply)  (Check all that apply)
	__	cigarette
	__	cigar
	__	pipe
	__	waterpipe/hookah
	__	other (specify)
	__	none
	__	Don't Know
	__	Refuse to Answer
	__	Not Applicable
If RTU33E is not equal to 1, then skip to instruction before RTU35.
RTU34.	TYP30A1S. Please specify other type of tobacco smoked during the past 30 days
	__ __ __ __ __ __ __ __ __ __ __ __ __ __ __ __ __ __ __ __
If RTU31A is not equal to 1, then skip to instruction before RTU36.
RTU35.	LIFEA1. Has [Response to RES24] smoked more than 100 cigarettes in their lifetime?
	1	Yes
	0	No
	7	Don't Know
	8	Refuse to Answer
	9	Not Applicable
If RTU33A is not equal to 1, then skip to instruction before RTU37.
RTU36.	SMKNOWA1. Does [Response to RES24] currently smoke cigarettes not at all, some days, or every day?  (Choose one)
	0	Not at All
	1	Some Days
	2	Every Day
	7	Don't Know
	8	Refuse to Answer
	9	Not Applicable
If RES29 is equal to "Not Applicable" or RES29 is equal to "skipped" or RES29 is equal to "Refuse to Answer" or RES29 is equal to "Don't Know", then skip to RTU61.
RTU37.	TYPEA2. Which types of tobacco has [Response to RES29] ever smoked, even a puff? 
(mark all that apply)  (Check all that apply)
	__	cigarette
	__	cigar
	__	pipe
	__	waterpipe/hookah
	__	other (specify)
	__	none
	__	Don't Know
	__	Refuse to Answer
	__	Not Applicable
If RTU37F is equal to 1, then skip to instruction before RTU43.
If RTU37E is not equal to 1, then skip to RTU39.
RTU38.	TYPEA2S. Please specify other type of tobacco smoked ever
	__ __ __ __ __ __ __ __ __ __ __ __ __ __ __ __ __ __ __ __
RTU39.	TYP30A2. During the past 30 days which of the following types of tobacco has [Response to RES29] smoked? 
(mark all that apply)  (Check all that apply)
	__	cigarette
	__	cigar
	__	pipe
	__	waterpipe/hookah
	__	other (specify)
	__	none
	__	Don't Know
	__	Refuse to Answer
	__	Not Applicable
If RTU39E is not equal to 1, then skip to instruction before RTU41.
RTU40.	TYP30A2S. Please specify other type of tobacco smoked during the past 30 days
	__ __ __ __ __ __ __ __ __ __ __ __ __ __ __ __ __ __ __ __
If RTU37A is not equal to 1, then skip to instruction before RTU42.
RTU41.	LIFEA2. Has [Response to RES29] smoked more than 100 cigarettes in their lifetime?
	1	Yes
	0	No
	7	Don't Know
	8	Refuse to Answer
	9	Not Applicable
If RTU39A is not equal to 1, then skip to instruction before RTU43.
RTU42.	SMKNOWA2. Does [Response to RES29] currently smoke cigarettes not at all, some days, or every day?  (Choose one)
	0	Not at All
	1	Some Days
	2	Every Day
	7	Don't Know
	8	Refuse to Answer
	9	Not Applicable
If RES32 is equal to "Not Applicable" or RES32 is equal to "skipped" or RES32 is equal to "Refuse to Answer" or RES32 is equal to "Don't Know", then skip to RTU61.
RTU43.	TYPEA3. Which types of tobacco has [Response to RES32] ever smoked, even a puff? 
(mark all that apply)  (Check all that apply)
	__	cigarette
	__	cigar
	__	pipe
	__	waterpipe/hookah
	__	other (specify)
	__	none
	__	Don't Know
	__	Refuse to Answer
	__	Not Applicable
If RTU43F is equal to 1, then skip to instruction before RTU49.
If RTU43E is not equal to 1, then skip to RTU45.
RTU44.	TYPEA3S. Please specify other type of tobacco smoked ever
	__ __ __ __ __ __ __ __ __ __ __ __ __ __ __ __ __ __ __ __
RTU45.	TYP30A3. During the past 30 days which of the following types of tobacco has [Response to RES32] smoked? 
(mark all that apply)  (Check all that apply)
	__	cigarette
	__	cigar
	__	pipe
	__	waterpipe/hookah
	__	other (specify)
	__	none
	__	Don't Know
	__	Refuse to Answer
	__	Not Applicable
If RTU45E is not equal to 1, then skip to instruction before RTU47.
RTU46.	TYP30A3S. Please specify other type of tobacco smoked during the past 30 days
	__ __ __ __ __ __ __ __ __ __ __ __ __ __ __ __ __ __ __ __
If RTU43A is not equal to 1, then skip to instruction before RTU48.
RTU47.	LIFEA3. Has [Response to RES32] smoked more than 100 cigarettes in their lifetime?
	1	Yes
	0	No
	7	Don't Know
	8	Refuse to Answer
	9	Not Applicable
If RTU45A is not equal to 1, then skip to instruction before RTU49.
RTU48.	SMKNOWA3. Does [Response to RES32] currently smoke cigarettes not at all, some days, or every day?  (Choose one)
	0	Not at All
	1	Some Days
	2	Every Day
	7	Don't Know
	8	Refuse to Answer
	9	Not Applicable
If RES35 is equal to "Not Applicable" or RES35 is equal to "skipped" or RES35 is equal to "Refuse to Answer" or RES35 is equal to "Don't Know", then skip to RTU61.
RTU49.	TYPEA4. Which types of tobacco has [Response to RES35] ever smoked, even a puff? 
(mark all that apply)  (Check all that apply)
	__	cigarette
	__	cigar
	__	pipe
	__	waterpipe/hookah
	__	other (specify)
	__	none
	__	Don't Know
	__	Refuse to Answer
	__	Not Applicable
If RTU49F is equal to 1, then skip to instruction before RTU55.
If RTU49E is not equal to 1, then skip to RTU51.
RTU50.	TYPEA4S. Please specify other type of tobacco smoked ever
	__ __ __ __ __ __ __ __ __ __ __ __ __ __ __ __ __ __ __ __
RTU51.	TYP30A4. During the past 30 days which of the following types of tobacco has [Response to RES35] smoked? 
(mark all that apply)  (Check all that apply)
	__	cigarette
	__	cigar
	__	pipe
	__	waterpipe/hookah
	__	other (specify)
	__	none
	__	Don't Know
	__	Refuse to Answer
	__	Not Applicable
If RTU51E is not equal to 1, then skip to instruction before RTU53.
RTU52.	TYP30A4S. Please specify other type of tobacco smoked during the past 30 days
	__ __ __ __ __ __ __ __ __ __ __ __ __ __ __ __ __ __ __ __
If RTU49A is not equal to 1, then skip to instruction before RTU54.
RTU53.	LIFEA4. Has [Response to RES35] smoked more than 100 cigarettes in their lifetime?
	1	Yes
	0	No
	7	Don't Know
	8	Refuse to Answer
	9	Not Applicable
If RTU51A is not equal to 1, then skip to instruction before RTU55.
RTU54.	SMKNOWA4. Does [Response to RES35] currently smoke cigarettes not at all, some days, or every day?  (Choose one)
	0	Not at All
	1	Some Days
	2	Every Day
	7	Don't Know
	8	Refuse to Answer
	9	Not Applicable
If RES38 is equal to "Not Applicable" or RES38 is equal to "skipped" or RES38 is equal to "Refuse to Answer" or RES38 is equal to "Don't Know", then skip to RTU61.
RTU55.	TYPEA5. Which types of tobacco has [Response to RES38] ever smoked, even a puff? 
(mark all that apply)  (Check all that apply)
	__	cigarette
	__	cigar
	__	pipe
	__	waterpipe/hookah
	__	other (specify)
	__	none
	__	Don't Know
	__	Refuse to Answer
	__	Not Applicable
If RTU55F is equal to 1, then skip to RTU61.
If RTU55E is not equal to 1, then skip to RTU57.
RTU56.	TYPEA5S. Please specify other type of tobacco smoked ever
	__ __ __ __ __ __ __ __ __ __ __ __ __ __ __ __ __ __ __ __
RTU57.	TYP30A5. During the past 30 days which of the following types of tobacco has [Response to RES38] smoked? 
(mark all that apply)  (Check all that apply)
	__	cigarette
	__	cigar
	__	pipe
	__	waterpipe/hookah
	__	other (specify)
	__	none
	__	Don't Know
	__	Refuse to Answer
	__	Not Applicable
If RTU57E is not equal to 1, then skip to instruction before RTU59.
RTU58.	TYP30A5S. Please specify other type of tobacco smoked during the past 30 days
	__ __ __ __ __ __ __ __ __ __ __ __ __ __ __ __ __ __ __ __
If RTU55A is not equal to 1, then skip to instruction before RTU60.
RTU59.	LIFEA5. Has [Response to RES38] smoked more than 100 cigarettes in their lifetime?
	1	Yes
	0	No
	7	Don't Know
	8	Refuse to Answer
	9	Not Applicable
If RTU57A is not equal to 1, then skip to RTU61.
RTU60.	SMKNOWA5. Does [Response to RES38] currently smoke cigarettes not at all, some days, or every day?  (Choose one)
	0	Not at All
	1	Some Days
	2	Every Day
	7	Don't Know
	8	Refuse to Answer
	9	Not Applicable
RTU61.	SHARED. Is there a tobacco smoker living on the other side of a shared wall, or above or below your home? (check all that apply)
INTERVIEWER: If none apply, click "Next Question".  (Check all that apply)
	__	other side of wall
	__	above
	__	below
	__	Don't Know
	__	Refuse to Answer
	__	Not Applicable
RTU62.	JUMPBK7
Interviewer: Do you need to jump to a previous section?
If so, click YES to return to the start of the interview, where you can then choose to jump ahead to any section.
Click NO to continue.
	1	Yes
	0	No
	7	Don't Know
	8	Refuse to Answer
	9	Not Applicable
If RTU62 is equal to 1 then  skip to PRE4.

SECTION HTU: Home Tobacco Use
The next questions are about different types of smoking. It is important that you answer each question as accurately as possible.  Your answers will be kept strictly confidential.
HTU1.	CIG7. How often in the past 7 days did anyone smoke cigarettes in your home?  (Choose one)
	0	never
	1	1 to 3 times
	2	4 to 6 times
	3	7 to 9 times
	4	10 or more times
	7	Don't Know
	8	Refuse to Answer
	9	Not Applicable
HTU2.	CIGAR7. How often in the past 7 days did anyone smoke cigars in your home?  (Choose one)
	0	never
	1	1 to 3 times
	2	4 to 6 times
	3	7 to 9 times
	4	10 or more times
	7	Don't Know
	8	Refuse to Answer
	9	Not Applicable
HTU3.	PIPE7. How often in the past 7 days did anyone smoke pipe tobacco in your home?  (Choose one)
	0	never
	1	1 to 3 times
	2	4 to 6 times
	3	7 to 9 times
	4	10 or more times
	7	Don't Know
	8	Refuse to Answer
	9	Not Applicable
HTU4.	HOOKAH7. How often in the past 7 days did anyone smoke hookah/waterpipe in your home?  (Choose one)
	0	never
	1	1 to 3 times
	2	4 to 6 times
	3	7 to 9 times
	4	10 or more times
	7	Don't Know
	8	Refuse to Answer
	9	Not Applicable
HTU5.	ECIG7. How often in the past 7 days did anyone smoke electronic cigarettes in your home?  (Choose one)
	0	never
	1	1 to 3 times
	2	4 to 6 times
	3	7 to 9 times
	4	10 or more times
	7	Don't Know
	8	Refuse to Answer
	9	Not Applicable
HTU6.	MJ7. How often in the past 7 days did anyone smoke medicinal or recreational marijuana in your home?  (Choose one)
	0	never
	1	1 to 3 times
	2	4 to 6 times
	3	7 to 9 times
	4	10 or more times
	7	Don't Know
	8	Refuse to Answer
	9	Not Applicable
HTU7.	DRUGS7. How often in the past 7 days did anyone smoke other recreational drugs in your home?  (Choose one)
	0	never
	1	1 to 3 times
	2	4 to 6 times
	3	7 to 9 times
	4	10 or more times
	7	Don't Know
	8	Refuse to Answer
	9	Not Applicable
HTU8.	COFFEE7. How often in the past 7 days did anyone drink coffee while smoking in your home?  (Choose one)
	0	never
	1	1 to 3 times
	2	4 to 6 times
	3	7 to 9 times
	4	10 or more times
	7	Don't Know
	8	Refuse to Answer
	9	Not Applicable
HTU9.	ALCOHOL7. How often in the past 7 days did anyone drink alcohol while smoking in your home?  (Choose one)
	0	never
	1	1 to 3 times
	2	4 to 6 times
	3	7 to 9 times
	4	10 or more times
	7	Don't Know
	8	Refuse to Answer
	9	Not Applicable

SECTION SSE: Secondhand Smoke Exposure
The next questions are about [Response to RES1]s exposure to different types of tobacco smoke. Please use whole numbers when answering.  For example, if [Response to RES1] was exposed to one cigarette, even for a short time, please report one cigarette. If [Response to RES1] was exposed to three cigarettes, even for a short time, please report three cigarettes.
Interviewer: Section 7 of 12: SSE: Secondhand Smoke Exposure
SSE1.	CIGEXP. In the past 7 days, was [Response to RES1] exposed to any cigarettes in your home, a car, or any other place?
INTERVIEWER: "Other places" include garage, patio, front yard, back yard, park, etc.
If none apply, click "Next Question".
	1	Yes
	0	No
	7	Don't Know
	8	Refuse to Answer
	9	Not Applicable
If SSE1 is equal to 0, then skip to SSE4.
SSE2.	CIGLOC. [IF YES cigarettes]  
Where? (check all that apply)  (Check all that apply)	__	your home
	__	a car
	__	other place
	__	Don't Know
	__	Refuse to Answer
	__	Not Applicable
SSE3.	CIGNUM. [IF YES cigarettes]  
What was the average number of cigarettes per day that [Response to RES1] was exposed to in your home, a car, or any other place in the past 7 days?
	__ __ __
	997	Don't Know
	998	Refuse to Answer
	999	Not Applicable
SSE4.	OTHREXP. In the past 7 days, was [Response to RES1] exposed to any other tobacco products in the home, a car, or any other place? (check all that apply)
INTERVIEWER: "Other places" include garage, patio, front yard, back yard, park, etc.
If none apply, click "Next Question".  (Check all that apply)
	__	cigar
	__	pipes
	__	waterpipe/hookah
	__	Don't Know
	__	Refuse to Answer
	__	Not Applicable
If SSE4A is equal to 0 or SSE4A is equal to "Not Applicable" or SSE4A is equal to "Refuse to Answer" or SSE4A is equal to "Don't Know" or SSE4A is equal to "skipped", then skip to instruction before SSE6.
SSE5.	CIGARNUM. [If YES cigars] 
What was the average number of cigars per day that [Response to RES1] was exposed to in your home, a car, or any other place in the past 7 days?
	__ __ __
	997	Don't Know
	998	Refuse to Answer
	999	Not Applicable
If SSE4B is equal to 0 or SSE4B is equal to "Not Applicable" or SSE4B is equal to "Refuse to Answer" or SSE4B is equal to "Don't Know" or SSE4B is equal to "skipped", then skip to instruction before SSE7.
SSE6.	PIPENUM. [If YES pipes] 
What was the average number of pipes per day that [Response to RES1] was exposed to in your home, a car, or any other place in the past 7 days?
	__ __ __
	997	Don't Know
	998	Refuse to Answer
	999	Not Applicable
If SSE4C is equal to 0 or SSE4C is equal to "Not Applicable" or SSE4C is equal to "Refuse to Answer" or SSE4C is equal to "Don't Know" or SSE4C is equal to "skipped", then skip to SSE8.
SSE7.	HOOKNUM. [If YES waterpipe/hookah]
What was the average number of hookah heads per day that [Response to RES1] was exposed to in your home, a car, or any other place in the past 7 days?
	__ __ __
	997	Don't Know
	998	Refuse to Answer
	999	Not Applicable
SSE8.	JUMPBK8
Interviewer: Do you need to jump to a previous section?
If so, click YES to return to the start of the interview, where you can then choose to jump ahead to any section.
Click NO to continue.
	1	Yes
	0	No
	7	Don't Know
	8	Refuse to Answer
	9	Not Applicable
If SSE8 is equal to 1 then Interviewer: Do not read. You have selected to jump back to the beginning of the interview. and skip to PRE4.

SECTION SR: Smoking Rules in the Home
Now I have some questions about the smoking rules in your home.
Interviewer: Section 8 of 12: SR: Smoking Rules in the Home
SR1.	HOMRULE. How is tobacco smoking handled in your home? (check all that apply)  (Check all that apply)
	__	no one allowed to smoke in the house
	__	certain people or special guests allowed to smoke
	__	allow smoking in certain areas inside the home
	__	allow smoking anywhere inside the home
	__	Don't Know
	__	Refuse to Answer
	__	Not Applicable	Skip to SR16
If SR1D is equal to 1, then skip to SR16.
If SR1B is not equal to 1, then skip to SR4.
SR2.	CERTAIN - If certain people or special guests are allowed to smoke tobacco, who are these people? (check all that apply)
INTERVIEWER: If none apply, click "Next Question".  (Check all that apply)
	__	Mother, father, mother-in-law, or father-in-law
	__	Siblings
	__	Other close relatives
	__	Friends of the family
	__	Acquaintances
	__	Other persons (specify)
	__	Don't Know
	__	Refuse to Answer
	__	Not Applicable
If SR2F is not equal to 1, then skip to SR4.
SR3.	CERTSP. Specify Other: certain people or special guests who are allowed to smoke
	__ __ __ __ __ __ __ __ __ __ __ __ __ __ __ __ __ __ __ __
SR4.	LONG. About how long have you had these rules about tobacco smoking in your home?
Interviewer: Select unit of time on the next screen.
	__ __ __
	997	Don't Know
	998	Refuse to Answer
	999	Not Applicable
SR5.	LONGUT. Interviewer: Select unit of time that the participant has had these rules about tobacco smoking in the home  (Choose one)
	1	days
	2	weeks
	3	months
	4	years
	7	Don't Know
	8	Refuse to Answer
	9	Not Applicable
SR6.	BROKEN. About how often are the rules about tobacco smoking in your home broken?  (Choose one)
	00	Never
	01	less than once a year
	02	once a year
	03	once every several months
	04	once a month
	05	once a week
	06	daily
	07	more than once a day
	97	Don't Know
	98	Refuse to Answer
	99	Not Applicable
SR7.	ENFORCE. How are the rules about tobacco smoking in your home enforced? (check all that apply)
INTERVIEWER: If none apply, click "Next Question".  (Check all that apply)
	__	Telling people not to smoke in the home
	__	Keeping ashtrays out of the home
	__	Putting up No Smoking signs
	__	Asking people to go outside when they want to smoke
	__	Other ways (specify)
	__	Don't Know
	__	Refuse to Answer
	__	Not Applicable
If SR7E is not equal to 1, then skip to SR9.
SR8.	ENFORCSP. Specify Other: how are rules in home enforced
	__ __ __ __ __ __ __ __ __ __ __ __ __ __ __ __ __ __ __ __
SR9.	WHORULE. Who was mostly responsible for establishing the rules on smoking inside your current home?  (Choose one)
	01	The Participant
	02	Their Spouse/Partner
	03	Their Children
	04	Their Parents
	05	Their Siblings
	06	Their Roomate(s)
	07	Other Residents
	08	Other Nonresidents
	97	Don't Know
	98	Refuse to Answer
	99	Not Applicable
If SR9 is not equal to 7, then skip to instruction before SR11.
SR10.	SRRES_SP. Specify the resident who was mostly responsible for establishing the rules on smoking inside your current home.
	__ __ __ __ __ __ __ __ __ __ __ __ __ __ __ __ __ __ __ __
If SR9 is not equal to 8, then skip to SR12.
SR11.	SRNON_SP. Specify the non-resident who was mostly responsible for establishing the rules on smoking inside your current home.
	__ __ __ __ __ __ __ __ __ __ __ __ __ __ __ __ __ __ __ __
SR12.	SRHELP. How much do the household residents help to enforce the rules or customs regarding smoking?  (Choose one)
	1	A lot
	2	Somewhat
	3	Not at all
	7	Don't Know
	8	Refuse to Answer
	9	Not Applicable
SR13.	SRQUIT. To what degree have the rules helped a household resident quit or reduce smoking? Have they helped…  (Choose one)
	1	A lot
	2	Somewhat
	3	Not at all
	7	Don't Know
	8	Refuse to Answer
	9	Not Applicable
SR14.	SRWHY. Did you create a home ban on tobacco smoking for any or all of the following reasons?  (Check all that apply)
	__	to protect yourself from secondhand smoke
	__	to protect your youngest child from secondhand smoke
	__	to protect others from secondhand smoke
	__	to encourage smoking residents to quit or reduce smoking
	__	to avoid odors from tobacco smoking
	__	to avoid being bothered by tobacco smoke
	__	to avoid bothering others with tobacco smoke
	__	Other (specify)
	__	Don't Know
	__	Refuse to Answer
	__	Not Applicable
If SR14H is not equal to 1, then skip to SR16.
SR15.	SRWHYSP. Specify Other: reason for creating a home ban on tobacco smoking
	__ __ __ __ __ __ __ __ __ __ __ __ __ __ __ __ __ __ __ __
SR16.	INTENT. Which of the following best describes your intentions to set up a rule that bans smoking in your home? Would you say that you…  (Choose one)
	1	Never expect to set up a rule that bans smoking in your home
	2	May set up a rule in the future, but not in the next 6 months
	3	Will set up a rule in the next 6 months
	4	Will set up a rule in the next month
	7	Don't Know
	8	Refuse to Answer
	9	Not Applicable
SR17.	CONSID. Would you consider creating a home ban on tobacco smoking  for any or all of the following reasons? (check all that apply)  (Check all that apply)
	__	to protect yourself from secondhand smoke
	__	to protect your youngest child from secondhand smoke
	__	to protect others from secondhand smoke
	__	to encourage smoking residents to quit or reduce smoking
	__	to avoid odors from tobacco smoking
	__	to avoid being bothered by tobacco smoke
	__	to avoid bothering others with tobacco smoke
	__	other (specify)
	__	Don't Know
	__	Refuse to Answer
	__	Not Applicable
If SR17H is not equal to 1, then skip to SR19.
SR18.	CONSIDSP. Specify the reason for creating a home ban on tobacco smoking
	__ __ __ __ __ __ __ __ __ __ __ __ __ __ __ __ __ __ __ __
SR19.	HOMRUL2. How is smoking something other than tobacco (for example, marijuana) handled in your home? (check all that apply)  (Check all that apply)
	__	no one allowed to smoke in the house
	__	certain people or special guests allowed to smoke
	__	allow smoking in certain areas inside the home
	__	allow smoking anywhere inside the home
	__	Don't Know
	__	Refuse to Answer
	__	Not Applicable
SR20.	JUMPBK9
Interviewer: Do you need to jump to a previous section?
If so, click YES to return to the start of the interview, where you can then choose to jump ahead to any section.
Click NO to continue.
	1	Yes
	0	No
	7	Don't Know
	8	Refuse to Answer
	9	Not Applicable
If SR20 is equal to 1 then  skip to PRE4.


SECTION AV: Aversion to Secondhand Smoke Exposure
Please, tell me how much you agree with each of the following statements. Would you strongly agree, somewhat agree, or do not agree at all?
Interviewer: Section 9 of 12: AV: Aversion to Secondhand Smoke Exposure
AV1.	AVBOTHER. I feel bothered when somebody smokes around me.  (Choose one)
	1	strongly agree
	2	somewhat agree
	3	do not agree at all
	7	Don't Know
	8	Refuse to Answer
	9	Not Applicable
AV2.	AVWORK. I prefer to work in smoke-free workplaces  (Choose one)
	1	strongly agree
	2	somewhat agree
	3	do not agree at all
	7	Don't Know
	8	Refuse to Answer
	9	Not Applicable
AV3.	AVPUBLIC. I prefer to be in smoke-free public places, such as restaurants, movie theaters, and public transportation  (Choose one)
	1	strongly agree
	2	somewhat agree
	3	do not agree at all
	7	Don't Know
	8	Refuse to Answer
	9	Not Applicable
AV4.  AVBAN. How much do you support or would you support a law banning smoking inside [Workplaces, Restaurants, ...]? Would you support it a lot, somewhat, or not at all?  (Choose one)
	1	a lot
	2	somewhat
	3	not at all
	7	Don't Know
	8	Refuse to Answer
	9	Not Applicable

	AV4 	
Workplaces 	|__| 	
Restaurants 	|__| 	
Public Transportation 	|__| 	
Schools 	|__| 	
Health centers and hospitals 	|__| 	

AV5.	JUMPBK10
Interviewer: Do you need to jump to a previous section?
If so, click YES to return to the start of the interview, where you can then choose to jump ahead to any section.
Click NO to continue.
	1	Yes
	0	No
	7	Don't Know
	8	Refuse to Answer
	9	Not Applicable
If AV5 is equal to 1 then  skip to PRE4.
SECTION SI: Social Influences
The following questions are about your family and friends.
INTERVIEWER: If the participant does not have a particular category of family/friends (e.g., grandparents are all deceased) click "Not Applicable" for that question.
Interviewer: Section 10 of 12: SI: Social Influences
SI1.	FRIENDS. When you and [Response to RES1] visit the home of a friend who smokes, how often do you ask the friend not to smoke around [Response to RES1]?  (Choose one)
	0	Never
	1	Rarely
	2	Sometimes
	3	Usually
	4	Always
	5	Never, because no friends smoke around &[TC]
	7	Don't Know
	8	Refuse to Answer
	9	Not Applicable
SI2.	FAMILY. When you and [Response to RES1] visit the home of a family member who smokes, how often do you ask the family member not to smoke around [Response to RES1]?  (Choose one)
	0	Never
	1	Rarely
	2	Sometimes
	3	Usually
	4	Always
	5	Never, because no family members smoke around &[TC]
	7	Don't Know
	8	Refuse to Answer
	9	Not Applicable
SI3.  ENCOUR. Do(es) [partner, parents, ...] encourage, discourage, or neither encourage nor discourage, smoking in your home?  (Choose one)
	1	encourage
	2	discourage
	3	neither encourage nor discourage
	7	Don't Know
	8	Refuse to Answer
	9	Not Applicable

	SI3 	
partner 	|__| 	
parents 	|__| 	
siblings 	|__| 	
friends 	|__| 	
children  	|__| 	
grandparents 	|__| 	
aunts/uncles 	|__| 	
co-workers 	|__| 	
healthcare providers 	|__| 	
anyone else? 	|__| 	
If SI3J is equal to "Not Applicable" or SI3J is equal to "skipped" or SI3J is equal to "Don't Know" or SI3J is equal to "Refuse to Answer", then skip to SI5.

SI4.	ENCOURJS. Specify Other: Who else regularly encourages, discourages or neither encourages nor discourages smoking in your home?
	__ __ __ __ __ __ __ __ __ __ __ __ __ __ __ __ __ __ __ __
SI5.	CRITICIZ. How often have you seen someone criticized for smoking in public?  (Choose one)
	0	not at all
	1	not very often
	2	often
	3	very often
	7	Don't Know
	8	Refuse to Answer
	9	Not Applicable
SI6.	PRAISE. How often have you seen someone praised for putting out a cigarette while smoking in public?  (Choose one)
	0	not at all
	1	not very often
	2	often
	3	very often
	7	Don't Know
	8	Refuse to Answer
	9	Not Applicable
SI7.	DISAPPRV. How often have you seen someone show signs of disapproval when they see others smoking in public?  (Choose one)
	0	not at all
	1	not very often
	2	often
	3	very often
	7	Don't Know
	8	Refuse to Answer
	9	Not Applicable
SI8.	PERCENT. What percentage of smokers in California do you think will be asked not to smoke around children in public?
	__ __ __
	997	Don't Know
	998	Refuse to Answer
	999	Not Applicable
SI9.	JUMPBK11
Interviewer: Do you need to jump to a previous section?
If so, click YES to return to the start of the interview, where you can then choose to jump ahead to any section.
Click NO to continue.
	1	Yes
	0	No
	7	Don't Know
	8	Refuse to Answer
	9	Not Applicable
If SI9 is equal to 1 then  skip to PRE4.
SECTION DMG: Demographics
Just a few more questions for you.
Interviewer: Section 11 of 12: DMG: Demographics
DMG1.	EDUC. What is the highest level of education that you completed?  (Choose one)
	01	Never went to school
	02	Some elementary school
	03	Elementary school
	04	Junior high school
	05	High School
	06	Trade, Vocational Training
	07	College, no degree
	08	College degree
	09	Graduate studies or Doctoral
	10	Other (specify):_____________
	97	Don't Know
	98	Refuse to Answer
	99	Not Applicable
If DMG1 is equal to 1, then skip to DMG4.
If DMG1 is not equal to 10, then skip to DMG3.
DMG2.	EDUCSP. Please specify the other type of education you received that is not listed.
	__ __ __ __ __ __ __ __ __ __ __ __ __ __ __ __ __ __ __ __
DMG3.	EDUCYRS. How many years of education did you complete?
	__ __ . __
	97	Don't Know
	98	Refuse to Answer
	99	Not Applicable
DMG4.	COUNTRY. In what country were you born?
	__ __ __ __ __ __ __ __ __ __ __ __ __ __ __ __ __ __ __ __
DMG5.	EMPLOY. What is your current employment status?  (Choose one)
	0	Not currently employed
	1	Part-time employed
	2	Full-time Employed
	3	Homemaker
	4	Retired
	7	Don't Know
	8	Refuse to Answer
	9	Not Applicable
If DMG5 is equal to 0 or DMG5 is equal to 3 or DMG5 is equal to 4, then skip to DMG7.
DMG6.	WORKRULE. What are the rules regarding smoking INSIDE your workplace?  (Choose one)
	1	No one is allowed to smoke inside the workplace
	2	People are allowed to smoke only in certain area inside the workplace
	3	Only special people, such as customers or visitors are allowed to smoke inside the workplace
	4	Everyone is allowed to smoke everywhere
	7	Don't Know
	8	Refuse to Answer
	9	Not Applicable
DMG7.	MARITAL. What is your current marital status?  (Choose one)
	1	Single/Never Married
	2	Married
	3	Not married but living with partner
	4	Divorced/Separated
	5	Widowed
	7	Don't Know
	8	Refuse to Answer
	9	Not Applicable
DMG8.	STUDENT. What is your current student status?  (Choose one)
	0	Not currently enrolled as a student
	1	Part-time student
	2	Full-time student
	7	Don't Know
	8	Refuse to Answer
	9	Not Applicable
DMG9.	TPHISP. Do you consider yourself Hispanic or Latino? Hispanic or Latino? That is, a person of Spanish culture or origin, such as a Mexican, Central American, Puerto Rican, or Cuban?
	1	Yes
	0	No
	7	Don't Know
	8	Refuse to Answer
	9	Not Applicable
DMG10.	TPRACE. Which of the following best describes your race? Select all that apply.  (Check all that apply)
	__	American Indian or Alaskan Native
	__	Asian
	__	Native Hawaiian or Other Pacific Islander
	__	Black or African American
	__	White (Caucasian)
	__	Other (specify)
	__	Don't Know
	__	Refuse to Answer
	__	Not Applicable
If DMG10G is not equal to 1, then skip to DMG12.
DMG11.	TPRACESP. Please specify your racial identity as best as you can.
	__ __ __ __ __ __ __ __ __ __ __ __ __ __ __ __ __ __ __ __
DMG12.	TCHISP. Do you consider [Response to RES1] Hispanic or Latino? That is, a person of Spanish culture or origin, such as a Mexican, Central American, Puerto Rican, or Cuban?
	1	Yes
	0	No
	7	Don't Know
	8	Refuse to Answer
	9	Not Applicable
DMG13.	TCRACE. Which of the following best describe [Response to RES1]'s race? Select all that apply.  (Check all that apply)
	__	American Indian or Alaskan Native
	__	Asian
	__	Native Hawaiian or Other Pacific Islander
	__	Black or African American
	__	White (Caucasian)
	__	Other (specify)
	__	Don't Know
	__	Refuse to Answer
	__	Not Applicable
If DMG13G is not equal to 1, then skip to DMG15.
DMG14.	TCRACESP. Please specify [Response to RES1]'s racial identify as best as you can..
	__ __ __ __ __ __ __ __ __ __ __ __ __ __ __ __ __ __ __ __
DMG15.	INCOME. What is your total combined household income before taxes in the past year? Please include income from all sources including wages, salaries, social security, retirement, benefits, and help from relatives or friends.

INTERVIEWER: Do not read choices aloud.
  (Choose one)
	00	less than $10,000
	01	$10,000 - $19,999
	02	$20,000 - $29,999
	03	$30,000 - $39,999
	04	$40,000 - $49,999
	05	$50,000 - $59,999
	06	$60,000 - $69,999
	07	$70,000 - $79,999
	08	$80,000 - $89,999
	09	$90,000 - $99,999
	10	$100,000 or more
	97	Don't Know
	98	Refuse to Answer
	99	Not Applicable
DMG16.	JUMPBK12
Interviewer: Do you need to jump to a previous section?
If so, click YES to return to the start of the interview, where you can then choose to jump ahead to any section.
Click NO to continue.
	1	Yes
	0	No
	7	Don't Know
	8	Refuse to Answer
	9	Not Applicable
If DMG16 is equal to 1 then  skip to PRE4.
That's all the questions we have for you. Thanks for your patience.
INTERVIEWER: Complete the following section at family's home or in your vehicle.
DMG17.	JUMPBK13
Interviewer: Do you need to jump to a previous section?
If so, click YES to return to the start of the interview, where you can then choose to jump ahead to any section.
Click NO to continue.
	1	Yes
	0	No
	7	Don't Know
	8	Refuse to Answer
	9	Not Applicable
If DMG17 is equal to 1 then  skip to PRE4.
SECTION ADM: Administrative Data
ADM1.	NDMOVE. Was the air nicotine dosimeter still in the same room in which it was installed, as shown on the Floor Plan?
	1	Yes
	0	No
ADM2.	MNTRMOVE. Was each air monitor still in the same room in which it was installed, as shown on the Floor Plan?
	1	Yes
	0	No
If ADM2 is equal to 1, then skip to ADM4.
ADM3.	MNTRSP. If NO, which monitor was moved? (check all that apply)
INTERVIEWER: If none apply, click "Next Question".  (Check all that apply)	__	Monitor 1
	__	Monitor 2
ADM4.	LANGUAGE. What language was the interview conducted in?  (Choose one)
	1	English
	2	Spanish
	3	Other (Specify)
	7	Don't Know
	8	Refuse to Answer
	9	Not Applicable
If ADM4 is not equal to 3, then skip to ADM5.
ADM4sp.	LANGSP. Please specify the language conducted for this interview.
	__ __ __ __ __ __ __ __ __ __ __ __ __ __ __ __ __ __ __ __
ADM4spa.	LANGPROX. If proxy was used, record relationship to respondent and age
	__ __ __ __ __ __ __ __ __ __ __ __ __ __ __ __ __ __ __ __
ADM5.	CESSINFO. Did TP ask you for any information about smoking cessation methods or programs?
	1	Yes
	0	No
If ADM5 is equal to 0, then skip to ADM7.
ADM6.	CESSCOMM. If YES, provide comments if possible:
	__ __ __ __ __ __ __ __ __ __ __ __ __ __ __ __ __ __ __ __
ADM7.	REFER. Did you refer a smoking adult to their health-care provider for information or assistance with smoking cessation?
	1	Yes
	0	No
	7	Don't Know
	8	Refuse to Answer
	9	Not Applicable
ADM8.	QUALITY. Please describe anything that you think might have affected the quality of this interview (if any; e.g., other family member present during interview):
	__ __ __ __ __ __ __ __ __ __ __ __ __ __ __ __ __ __ __ __
ADM9.	COMMENTS. Other comments that you think are important to know about this interview:
	__ __ __ __ __ __ __ __ __ __ __ __ __ __ __ __ __ __ __ __
ADM10.	ADVERSE. Did you note any potential adverse events during this interview? 
IF YES, COMPLETE ADVERSE EVENTS REPORT FORM ! ! ! )  
	1	Yes
	0	No
ADM11.	JUMPBK14
Interviewer: Do you need to jump to a previous section?
If so, click YES to return to the start of the interview, where you can then choose to jump ahead to any section.
Click NO to continue.
	1	Yes
	0	No
	7	Don't Know
	8	Refuse to Answer
	9	Not Applicable
If ADM11 is equal to 1 then  skip to PRE4.
