# Supplementary material for: Fine particles in homes of predominantly low-income families with children and smokers: Key physical and behavioral determinants to inform indoor-air-quality interventions
Source: PLoS One. 2017 May 17;12(5):e0177718. doi: 10.1371/journal.pone.0177718 (PMC5435241; doi:10.1371/journal.pone.0177718)
Supplement: S2 File — (RTF) [file pone.0177718.s006.rtf]

This is the variable codebook for the PLOS ONE manuscript entitled:
Fine Particles in Homes of Predominantly Low-Income Families with Children and Smokers: Key Physical and Behavioral Determinants to Inform Indoor-Air-Quality Interventions
by Neil Klepeis et al.    (pubs1@klepeis.net)
This is the Complete Codebook for the interview described in the above manuscript as implemented in QDS software:   http://www.novaresearch.com/QDS/ 
The interview was administered to participants on-site using laptop computers.

This research was supported with a grant from the National Heart, Lung, and Blood Institute of the National Institutes of Health from 2011 – 2015 under award number R01HL103684 (http://www.nhlbi.nih.gov/) to San Diego State University Research Foundation (Dr. M. Hovell, principal investigator, mhovell@cbeachsdsu.org).    Please address correspondence to Dr. Klepeis (pubs1@klepeis.net) or Dr. Hovell. 


PRE1.	SCRNID: 6 digit screening identification number, beginning with 700001.
Interviewer: pre-fill this number prior to starting the interview
SCRNID	SCRNID: 6 digit screening identification number	6
	700001 - 999999	=	range

PRE2.	HOMEID: 3 digit identification number for the home
Interviewer: pre-fill this number prior to starting the interview
HOMEID	HOMEID: 3 digit identification number for the home	3
	101 - 999	=	range

PRE3.	INTRVWER. Interviewer Initials
INTERVIEWER: Please type in your two lowercase initials.
Note that the on-screen buttons are uppercase.
INTRVWER	INTRVWER. Interviewer Initials	2

PRE4.	JUMP. Interviewer: Do you need to jump to another section? if so which one?
JUMP	JUMP. Skip ahead?	2
	1	=	No, continue to Introduction
	2	=	RES: Residents in the Home
	3	=	CH: Child Health
	4	=	HCV: Heating, Cooling, or Ventilation
	5	=	VNT: Ventilation Behaviors
	6	=	PGA: Particle Generating Activities
	7	=	RTU: Residents' Tobacco Use
	8	=	SSE: Secondhand Smoke Exposure
	9	=	SR: Smoking Rules in the Home
	10	=	AV: Aversion to Secondhand Smoke Exposure
	11	=	SI: Social Influences
	12	=	DMG: Demographics
	13	=	ADM: Administrative Data

Calculated Variable
DATE	Date of Interview
 	DATE = Today's date

Calculated Variable
STTIME	Start time of Interview
 	STTIME = Current time

INT1.	RECORD. My supervisor would like to make sure I have entered your answers correctly. Is it okay if I record this interview for my supervisor?
RECORD	RECORD. My supervisor would like to make sure I have entered your answers correctly. Is it okay if I record this interview for my supervisor?	1
	0	=	Participant opted for NO AUDIO RECORDING
	1	=	Participant agreed to have interview audio RECORDED
	2	=	Audio RECORDER NOT AVAILABLE for Interview
	7	=	Don't Know
	8	=	Refuse to Answer
	9	=	Not Applicable

RES1.	TC. What is [the Target Child]'s First Name? [Ask TP to confirm spelling]
INTERVIEWER: Enter TC's name without asking, or ask TP to confirm spelling.
TC	TC. What is [TC]'s First Name? [Ask TP to confirm spelling]	20

RES2.	DBTC. What is [Response to RES1]'s Date of Birth? [Ask TP to confirm date]
INTERVIEWER: Birthdate should be four-digit year (YYYY), one or two-digit month (MM), and one or two-digit day (DD). Example: 3/14/2012. 
DBTC	DBTC. What is [TC]'s Date of Birth? [Ask TP to confirm date]	8
	Unlimited - Unlimited	=	mm/dd/yyyy
	2097	=	Don't Know (Year)
	2098	=	Refuse to Answer (Year)
	2099	=	Not Applicable (Year)

RES3.	SEXTC. What is [Response to RES1]'s Gender?
INTERVIEWER: Only ask gender when needed. 
SEXTC	SEXTC. SEXTC. What is [TC]'s Gender?	1
	1	=	Male
	2	=	Female
	7	=	Don't Know
	8	=	Refuse to Answer
	9	=	Not Applicable

RES4.	FNCHLD2. Child 2 First Name
INTERVIEWER: If there is no Child 2, enter "Not Applicable".
FNCHLD2	FNCHLD2. Child 2 First Name	20

RES5.	DBCHLD2. Child 2 Date of Birth
INTERVIEWER: Birthdate should be  four-digit year (YYYY), a one or two-digit month (MM), and a one or two-digit day (DD). Example: 3/14/2012. 
DBCHLD2	DBCHLD2. Child 2 Date of Birth	8
	Unlimited - Unlimited	=	mm/dd/yyyy
	2097	=	Don't Know (Year)
	2098	=	Refuse to Answer (Year)
	2099	=	Not Applicable (Year)

RES6.	SEXCHLD2. Child 2 Gender
INTERVIEWER: Only ask gender when needed. 
SEXCHLD2	SEXCHLD2. Child 2 Gender	1
	1	=	Male
	2	=	Female
	7	=	Don't Know
	8	=	Refuse to Answer
	9	=	Not Applicable

RES7.	RELCHLD2. Child 2 Relationship to TC
INTERVIEWER: Do not read response options to participant
RELCHLD2	RELCHLD2. Child 2 Relationship to TC	2
	1	=	Brother
	2	=	Sister
	3	=	Cousin
	4	=	Biological mother
	5	=	Stepmother
	6	=	Adoptive mother
	7	=	Foster Mother
	8	=	Biological father
	9	=	Stepfather
	10	=	Adoptive father
	11	=	Foster father
	12	=	Legal guardian
	13	=	Grandmother
	14	=	Grandfather
	15	=	Aunt
	16	=	Uncle
	17	=	Other (Specify)
	97	=	Don't Know
	98	=	Refuse to Answer
	99	=	Not Applicable

RES8.	OTHCHLD2. Specify Other Relationship to [Response to RES1]
OTHCHLD2	OTHCHLD2. Specify Other Relationship to [TC]	20

RES9.	FNCHLD3. Child 3 First Name
INTERVIEWER: If there is no Child 3, enter "Not Applicable".
FNCHLD3	FNCHLD3. Child 3 First Name	20

RES10.	DBCHLD3. Child 3 Date of Birth
INTERVIEWER: Birthdate should be  four-digit year (YYYY), a one or two-digit month (MM), and a one or two-digit day (DD). Example: 3/14/2012. 
DBCHLD3	DBCHLD3. Child 3 Date of Birth	8
	Unlimited - Unlimited	=	mm/dd/yyyy
	2097	=	Don't Know (Year)
	2098	=	Refuse to Answer (Year)
	2099	=	Not Applicable (Year)

RES11.	SEXCHLD3. Child 3 Gender
INTERVIEWER: Only ask gender when needed. 
SEXCHLD3	SEXCHLD3. Child 3 Gender	1
	1	=	Male
	2	=	Female
	7	=	Don't Know
	8	=	Refuse to Answer
	9	=	Not Applicable

RES12.	RELCHLD3. Child 3 Relationship to TC
INTERVIEWER: Do not read response options to participant
RELCHLD3	RELCHLD3. Child 3 Relationship to TC	2
	1	=	Brother
	2	=	Sister
	3	=	Cousin
	4	=	Biological mother
	5	=	Stepmother
	6	=	Adoptive mother
	7	=	Foster Mother
	8	=	Biological father
	9	=	Stepfather
	10	=	Adoptive father
	11	=	Foster father
	12	=	Legal guardian
	13	=	Grandmother
	14	=	Grandfather
	15	=	Aunt
	16	=	Uncle
	17	=	Other (Specify)
	97	=	Don't Know
	98	=	Refuse to Answer
	99	=	Not Applicable

RES13.	OTHCHLD3. Specify Other Relationship to [Response to RES1]
OTHCHLD3	OTHCHLD3. Specify Other Relationship to [TC]	20

RES14.	FNCHLD4. Child 4 First Name
INTERVIEWER: If there is no Child 4, enter "Not Applicable".
FNCHLD4	FNCHLD4. Child 4 First Name	20

RES15.	DBCHLD4. Child 4 Date of Birth
INTERVIEWER: Birthdate should be  four-digit year (YYYY), a one or two-digit month (MM), and a one or two-digit day (DD). Example: 3/14/2012. 
DBCHLD4	DBCHLD4. Child 4 Date of Birth	8
	Unlimited - Unlimited	=	mm/dd/yyyy
	2097	=	Don't Know (Year)
	2098	=	Refuse to Answer (Year)
	2099	=	Not Applicable (Year)

RES16.	SEXCHLD4. Child 4 Gender
INTERVIEWER: Only ask gender when needed. 
SEXCHLD4	SEXCHLD4. Child 4 Gender	1
	1	=	Male
	2	=	Female
	7	=	Don't Know
	8	=	Refuse to Answer
	9	=	Not Applicable

RES17.	RELCHLD4. Child 4 Relationship to TC
INTERVIEWER: Do not read response options to participant
RELCHLD4	RELCHLD4. Child 4 Relationship to TC	2
	1	=	Brother
	2	=	Sister
	3	=	Cousin
	4	=	Biological mother
	5	=	Stepmother
	6	=	Adoptive mother
	7	=	Foster Mother
	8	=	Biological father
	9	=	Stepfather
	10	=	Adoptive father
	11	=	Foster father
	12	=	Legal guardian
	13	=	Grandmother
	14	=	Grandfather
	15	=	Aunt
	16	=	Uncle
	17	=	Other (Specify)
	97	=	Don't Know
	98	=	Refuse to Answer
	99	=	Not Applicable

RES18.	OTHCHLD4. Specify Other Relationship to [Response to RES1]
OTHCHLD4	OTHCHLD4. Specify Other Relationship to [TC]	20

RES19.	FNCHLD5. Child 5 First Name
INTERVIEWER: If there is no Child 5, enter "Not Applicable".
FNCHLD5	FNCHLD5. Child 5 First Name	20

RES20.	DBCHLD5. Child 5 Date of Birth
INTERVIEWER: Birthdate should be  four-digit year (YYYY), a one or two-digit month (MM), and a one or two-digit day (DD). Example: 3/14/2012. 
DBCHLD5	DBCHLD5. Child 5 Date of Birth	8
	Unlimited - Unlimited	=	mm/dd/yyyy
	2097	=	Don't Know (Year)
	2098	=	Refuse to Answer (Year)
	2099	=	Not Applicable (Year)

RES21.	SEXCHLD5. Child 5 Gender
INTERVIEWER: Only ask gender when needed. 
SEXCHLD5	SEXCHLD5. Child 5 Gender	1
	1	=	Male
	2	=	Female
	7	=	Don't Know
	8	=	Refuse to Answer
	9	=	Not Applicable

RES22.	RELCHLD5. Child 5 Relationship to TC
INTERVIEWER: Do not read response options to participant
RELCHLD5	RELCHLD5. Child 5 Relationship to TC	2
	1	=	Brother
	2	=	Sister
	3	=	Cousin
	4	=	Biological mother
	5	=	Stepmother
	6	=	Adoptive mother
	7	=	Foster Mother
	8	=	Biological father
	9	=	Stepfather
	10	=	Adoptive father
	11	=	Foster father
	12	=	Legal guardian
	13	=	Grandmother
	14	=	Grandfather
	15	=	Aunt
	16	=	Uncle
	17	=	Other (Specify)
	97	=	Don't Know
	98	=	Refuse to Answer
	99	=	Not Applicable

RES23.	OTHCHLD5. Specify Other Relationship to [Response to RES1]
OTHCHLD5	OTHCHLD5. Specify Other Relationship to [TC]	20

RES24.	TP. Adult 1 (Target Parent) First Name
TP	TP. Adult 1 (TP) First Name	20

RES25.	DBTP. Adult 1 (TP) Date of Birth
INTERVIEWER: Birthdate should be four-digit year (YYYY), one or two-digit month (MM), and one or two-digit day (DD). Example: 3/14/2012. 
DBTP	DBTP. Adult 1 (TP) Date of Birth	8
	Unlimited - Unlimited	=	mm/dd/yyyy
	2097	=	Don't Know (Year)
	2098	=	Refuse to Answer (Year)
	2099	=	Not Applicable (Year)

RES26.	SEXTP. Adult 1 (TP) Gender
INTERVIEWER: Only ask gender when needed. 
SEXTP	SEXTP. Adult 1 (TP) Gender	1
	1	=	Male
	2	=	Female
	7	=	Don't Know
	8	=	Refuse to Answer
	9	=	Not Applicable

RES27.	RELTP. Adult 1 (TP) Relationship to TC
INTERVIEWER: Do not read response options to participant
RELTP	RELTP. Adult 1 (TP) Relationship to TC	2
	1	=	Brother
	2	=	Sister
	3	=	Cousin
	4	=	Biological mother
	5	=	Stepmother
	6	=	Adoptive mother
	7	=	Foster Mother
	8	=	Biological father
	9	=	Stepfather
	10	=	Adoptive father
	11	=	Foster father
	12	=	Legal guardian
	13	=	Grandmother
	14	=	Grandfather
	15	=	Aunt
	16	=	Uncle
	17	=	Other (Specify)
	97	=	Don't Know
	98	=	Refuse to Answer
	99	=	Not Applicable

RES28.	OTHTP. Specify Other Relationship to [Response to RES1]
OTHTP	OTHTP. Specify Other Relationship to [TC]	20

RES29.	FNADLT2. Adult 2 First Name
INTERVIEWER: If there is no Adult 2, enter "Not Applicable".
FNADLT2	FNADLT2. Adult 2 First Name	20

RES30.	RELADLT2. Adult 2 Relationship to TC
INTERVIEWER: Do not read response options to participant
RELADLT2	RELADLT2. Adult 2 Relationship to TC	2
	1	=	Brother
	2	=	Sister
	3	=	Cousin
	4	=	Biological mother
	5	=	Stepmother
	6	=	Adoptive mother
	7	=	Foster Mother
	8	=	Biological father
	9	=	Stepfather
	10	=	Adoptive father
	11	=	Foster father
	12	=	Legal guardian
	13	=	Grandmother
	14	=	Grandfather
	15	=	Aunt
	16	=	Uncle
	17	=	Other (Specify)
	97	=	Don't Know
	98	=	Refuse to Answer
	99	=	Not Applicable

RES31.	OTHADLT2. Specify Other Relationship to [Response to RES1]
OTHADLT2	OTHADLT2. Specify Other Relationship to [TC]	20

RES32.	FNADLT3. Adult 3 First Name
INTERVIEWER: If there is no Adult 3, enter "Not Applicable".
FNADLT3	FNADLT3. Adult 3 First Name	20

RES33.	RELADLT3. Adult 3 Relationship to TC
INTERVIEWER: Do not read response options to participant
RELADLT3	RELADLT3. Adult 3 Relationship to TC	2
	1	=	Brother
	2	=	Sister
	3	=	Cousin
	4	=	Biological mother
	5	=	Stepmother
	6	=	Adoptive mother
	7	=	Foster Mother
	8	=	Biological father
	9	=	Stepfather
	10	=	Adoptive father
	11	=	Foster father
	12	=	Legal guardian
	13	=	Grandmother
	14	=	Grandfather
	15	=	Aunt
	16	=	Uncle
	17	=	Other (Specify)
	97	=	Don't Know
	98	=	Refuse to Answer
	99	=	Not Applicable

RES34.	OTHADLT3. Specify Other Relationship to [Response to RES1]
OTHADLT3	OTHADLT3. Specify Other Relationship to [TC]	20

RES35.	FNADLT4. Adult 4 First Name
INTERVIEWER: If there is no Adult 4, enter "Not Applicable".
FNADLT4	FNADLT4. Adult 4 First Name	20

RES36.	RELADLT4. Adult 4 Relationship to TC
INTERVIEWER: Do not read response options to participant
RELADLT4	RELADLT4. Adult 4 Relationship to TC	2
	1	=	Brother
	2	=	Sister
	3	=	Cousin
	4	=	Biological mother
	5	=	Stepmother
	6	=	Adoptive mother
	7	=	Foster Mother
	8	=	Biological father
	9	=	Stepfather
	10	=	Adoptive father
	11	=	Foster father
	12	=	Legal guardian
	13	=	Grandmother
	14	=	Grandfather
	15	=	Aunt
	16	=	Uncle
	17	=	Other (Specify)
	97	=	Don't Know
	98	=	Refuse to Answer
	99	=	Not Applicable

RES37.	OTHADLT4. Specify Other Relationship to [Response to RES1]
OTHADLT4	OTHADLT4. Specify Other Relationship to [TC]	20

RES38.	FNADLT5. Adult 5 First Name
INTERVIEWER: If there is no Adult 5, enter "Not Applicable".
FNADLT5	FNADLT5. Adult 5 First Name	20

RES39.	RELADLT5. Adult 5 Relationship to TC
INTERVIEWER: Do not read response options to participant
RELADLT5	RELADLT5. Adult 5 Relationship to TC	2
	1	=	Brother
	2	=	Sister
	3	=	Cousin
	4	=	Biological mother
	5	=	Stepmother
	6	=	Adoptive mother
	7	=	Foster Mother
	8	=	Biological father
	9	=	Stepfather
	10	=	Adoptive father
	11	=	Foster father
	12	=	Legal guardian
	13	=	Grandmother
	14	=	Grandfather
	15	=	Aunt
	16	=	Uncle
	17	=	Other (Specify)
	97	=	Don't Know
	98	=	Refuse to Answer
	99	=	Not Applicable

RES40.	OTHADLT5. Specify Other Relationship to [Response to RES1]
OTHADLT5	OTHADLT5. Specify Other Relationship to [TC]	20

RES41.	JUMPBK2
Interviewer: Do you need to jump to a previous section?
If so, click YES to return to the start of the interview, where you can then choose to jump ahead to any section.
Click NO to continue.
JUMPBK2	JUMPBK2. Jump back To a previous section?	1
	0	=	No
	1	=	Yes
	7	=	Don't Know
	8	=	Refuse to Answer
	9	=	Not Applicable

CH1.	SEENDOC. In the past year, has [Response to RES1] been to a doctor or other healthcare provider?
SEENDOC	SEENDOC. In the past year, has [TC] been to a doctor or other healthcare provider?	1
	0	=	No
	1	=	Yes
	7	=	Don't Know
	8	=	Refuse to Answer
	9	=	Not Applicable

CH2.	TCHAD. In the past year, has a doctor or other healthcare provider said [Response to RES1] had:
(check all that apply)
INTERVIEWER: If none apply, click "Next Question".  (Check all that apply)
TCHADA	TCHAD. In the past year, has a doctor or other healthcare provider said [TC] had:: a cold	1
	0	=	No
	1	=	Yes
	97	=	Don't Know
	98	=	Refuse to Answer
	99	=	Not Applicable

TCHADB	TCHAD. In the past year, has a doctor or other healthcare provider said [TC] had:: an ear infection	1
	0	=	No
	1	=	Yes
	97	=	Don't Know
	98	=	Refuse to Answer
	99	=	Not Applicable

TCHADC	TCHAD. In the past year, has a doctor or other healthcare provider said [TC] had:: bronchitis or bronchiolitis	1
	0	=	No
	1	=	Yes
	97	=	Don't Know
	98	=	Refuse to Answer
	99	=	Not Applicable

TCHADD	TCHAD. In the past year, has a doctor or other healthcare provider said [TC] had:: pneumonia	1
	0	=	No
	1	=	Yes
	97	=	Don't Know
	98	=	Refuse to Answer
	99	=	Not Applicable

TCHADE	TCHAD. In the past year, has a doctor or other healthcare provider said [TC] had:: asthma or reactive airway disease	1
	0	=	No
	1	=	Yes
	97	=	Don't Know
	98	=	Refuse to Answer
	99	=	Not Applicable

TCHADF	TCHAD. In the past year, has a doctor or other healthcare provider said [TC] had:: skin condition eczema or atopic dermatitis	1
	0	=	No
	1	=	Yes
	97	=	Don't Know
	98	=	Refuse to Answer
	99	=	Not Applicable

TCHADG	TCHAD. In the past year, has a doctor or other healthcare provider said [TC] had:: cystic fibrosis or other pulmonary problems	1
	0	=	No
	1	=	Yes
	97	=	Don't Know
	98	=	Refuse to Answer
	99	=	Not Applicable


CH3.	TCEROOM. How many times in the past year has [Response to RES1] been treated or seen at an emergency room for coughing or difficulty breathing?
TCEROOM	TCEROOM. How many times in the past year has [TC] been treated or seen at an emergency room for coughing or difficulty breathing?	2
	0 - 96	=	range
	97	=	Don't Know
	98	=	Refuse to Answer
	99	=	Not Applicable

CH4.	TCOVER. How many times in the past year has [Response to RES1] been admitted as an overnight patient in a hospital for coughing or difficulty breathing?
TCOVER	TCOVER. How many times in the past year has [TC] been admitted as an overnight patient in a hospital for coughing or difficulty breathing?	2
	0 - 96	=	range
	97	=	Don't Know
	98	=	Refuse to Answer
	99	=	Not Applicable

CH5.	TCHEALTH. In general, would you say [Response to RES1]'s health is excellent, very good, good, fair or poor?
TCHEALTH	TCHEALTH. In general, would you say [TC]'s health is excellent, very good, good, fair or poor?	1
	1	=	poor
	2	=	fair
	3	=	good
	4	=	very good
	5	=	excellent
	7	=	Don't Know
	8	=	Refuse to Answer
	9	=	Not Applicable

CH6.	JUMPBK3
Interviewer: Do you need to jump to a previous section?
If so, click YES to return to the start of the interview, where you can then choose to jump ahead to any section.
Click NO to continue.
JUMPBK3	JUMPBK3. Jump back To a previous section?	1
	0	=	No
	1	=	Yes
	7	=	Don't Know
	8	=	Refuse to Answer
	9	=	Not Applicable

HCV1.	WOOD7. In the past 7 days, on how many days did you or someone in your home use a wood burning stove or fireplace?
INTERVIEWER: Click "Not Applicable" if there is no such item/device in the home, to skip the next two questions.
WOOD7	WOOD7. In the past 7 days, on how many days did you or someone in your home use a wood burning stove or fireplace?	5
	0 - 7	=	range
	97	=	Don't Know
	98	=	Refuse to Answer
	99	=	Not Applicable

HCV2.	WOODHR. About how many hours per day did you or someone use a wood burning stove or fireplace?
WOODHR	WOODHR. About how many hours per day did you or someone use a wood burning stove or fireplace?	5
	0 - 24	=	range
	97	=	Don't Know
	98	=	Refuse to Answer
	99	=	Not Applicable

HCV3.	WOOD123. Was the wood burning stove or fireplace typically on low, medium, or high?
WOOD123	WOOD123. Was the wood burning stove or fireplace typically on low, medium, or high?	1
	1	=	low
	2	=	medium
	3	=	high
	7	=	Don't Know
	8	=	Refuse to Answer
	9	=	Not Applicable

HCV4.	GASH7. In the past 7 days, on how many days did you or someone in your home use a gas floor heater, space heater, or wall-mounted heater?
INTERVIEWER: Click "Not Applicable" if there is no such item/device in the home, to skip the next two questions.
GASH7	GASH7. In the past 7 days, on how many days did you or someone in your home use a gas floor heater, space heater, or wall-mounted heater?	5
	0 - 7	=	range
	97	=	Don't Know
	98	=	Refuse to Answer
	99	=	Not Applicable

HCV5.	GASHHR. About how many hours per day did you or someone use gas floor heater, space heater, or wall-mounted heater?
GASHHR	GASHHR. About how many hours per day did you or someone use gas floor heater, space heater, or wall-mounted heater?	5
	0 - 24	=	range
	97	=	Don't Know
	98	=	Refuse to Answer
	99	=	Not Applicable

HCV6.	GASH123. Was the gas floor heater, space heater, or wall-mounted heater typically on low, medium, or high?
GASH123	GASH123. Was the gas floor heater, space heater, or wall-mounted heater typically on low, medium, or high?	1
	1	=	low
	2	=	medium
	3	=	high
	7	=	Don't Know
	8	=	Refuse to Answer
	9	=	Not Applicable

HCV7.	CENT7. In the past 7 days, on how many days did you or someone in your home use a central air handling system (heating, cooling, or ventilation)?
INTERVIEWER: Click "Not Applicable" if there is no such item/device in the home, to skip the next two questions.
CENT7	CENT7. In the past 7 days, on how many days did you or someone in your home use a central air handling system (heating, cooling, or ventilation)?	5
	0 - 7	=	range
	97	=	Don't Know
	98	=	Refuse to Answer
	99	=	Not Applicable

HCV8.	CENTHR. About how many hours per day did you or someone use a central air handling system (heating, cooling, or ventilation)?
CENTHR	CENTHR. About how many hours per day did you or someone use a central air handling system (heating, cooling, or ventilation)?	5
	0 - 24	=	range
	97	=	Don't Know
	98	=	Refuse to Answer
	99	=	Not Applicable

HCV9.	CENT123. Was the central air handling system (heating, cooling, or ventilation) typically on low, medium, or high?
CENT123	CENT123. Was the central air handling system (heating, cooling, or ventilation) typically on low, medium, or high?	1
	1	=	low
	2	=	medium
	3	=	high
	7	=	Don't Know
	8	=	Refuse to Answer
	9	=	Not Applicable

HCV10.	PUR7. In the past 7 days, on how many days did you or someone in your home use an air purifier with a fan?
INTERVIEWER: Click "Not Applicable" if there is no such item/device in the home, to skip the next two questions.
PUR7	PUR7. In the past 7 days, on how many days did you or someone in your home use an air purifier with a fan?	5
	0 - 7	=	range
	97	=	Don't Know
	98	=	Refuse to Answer
	99	=	Not Applicable

HCV11.	PURHR. About how many hours per day did you or someone use an air purifier with a fan?
PURHR	PURHR. About how many hours per day did you or someone use an air purifier with a fan?	5
	0 - 24	=	range
	97	=	Don't Know
	98	=	Refuse to Answer
	99	=	Not Applicable

HCV12.	PUR123. Was the air purifier with a fan typically on low, medium, or high?
PUR123	PUR123. Was the air purifier with a fan typically on low, medium, or high?	1
	1	=	low
	2	=	medium
	3	=	high
	7	=	Don't Know
	8	=	Refuse to Answer
	9	=	Not Applicable

HCV13.	EXH7. In the past 7 days, on how many days did you or someone in your home use an exhaust fan in the kitchen?
INTERVIEWER: Click "Not Applicable" if there is no such item/device in the home, to skip the next two questions.
EXH7	EXH7. In the past 7 days, on how many days did you or someone in your home use an exhaust fan in the kitchen?	5
	0 - 7	=	range
	97	=	Don't Know
	98	=	Refuse to Answer
	99	=	Not Applicable

HCV14.	EXHHR. About how many hours per day did you or someone use an exhaust fan in the kitchen?
EXHHR	EXHHR. About how many hours per day did you or someone use an exhaust fan in the kitchen?	5
	0 - 24	=	range
	97	=	Don't Know
	98	=	Refuse to Answer
	99	=	Not Applicable

HCV15.	EXH123. Was the exhaust fan in the kitchen typically on low, medium, or high?
EXH123	EXH123. Was the exhaust fan in the kitchen typically on low, medium, or high?	1
	1	=	low
	2	=	medium
	3	=	high
	7	=	Don't Know
	8	=	Refuse to Answer
	9	=	Not Applicable

HCV16.	ACFAN7. In the past 7 days, on how many days did you or someone in your home use a window fan or window air conditioner?
INTERVIEWER: Click "Not Applicable" if there is no such item/device in the home, to skip the next two questions.
ACFAN7	ACFAN7. In the past 7 days, on how many days did you or someone in your home use a window fan or window air conditioner?	5
	0 - 7	=	range
	97	=	Don't Know
	98	=	Refuse to Answer
	99	=	Not Applicable

HCV17.	ACFANHR. About how many hours per day did you or someone use a window fan or window air conditioner?
ACFANHR	ACFANHR. About how many hours per day did you or someone use a window fan or window air conditioner?	5
	0 - 24	=	range
	97	=	Don't Know
	98	=	Refuse to Answer
	99	=	Not Applicable

HCV18.	ACFAN123. Was the window fan or window air conditioner typically on low, medium, or high?
ACFAN123	ACFAN123. Was the window fan or window air conditioner typically on low, medium, or high?	1
	1	=	low
	2	=	medium
	3	=	high
	7	=	Don't Know
	8	=	Refuse to Answer
	9	=	Not Applicable

HCV19.	WIN7. In the past 7 days, on how many days did you or someone in your home open a window?
WIN7	WIN7. In the past 7 days, on how many days did you or someone in your home open a window?	5
	0 - 7	=	range
	97	=	Don't Know
	98	=	Refuse to Answer
	99	=	Not Applicable

HCV20.	WINHR. About how many hours per day did you or someone open a window?
WINHR	WINHR. About how many hours per day did you or someone open a window?	5
	0 - 24	=	range
	97	=	Don't Know
	98	=	Refuse to Answer
	99	=	Not Applicable

HCV21.	DOOR7. In the past 7 days, on how many days did you or someone in your home open an exterior door?
INTERVIEWER: An exterior door is one leading outside the home.
DOOR7	DOOR7. In the past 7 days, on how many days did you or someone in your home open an exterior door?	5
	0 - 7	=	range
	97	=	Don't Know
	98	=	Refuse to Answer
	99	=	Not Applicable

HCV22.	DOORHR. About how many hours per day did you or someone open an exterior door?
DOORHR	DOORHR. About how many hours per day did you or someone open an exterior door?	5
	0 - 24	=	range
	97	=	Don't Know
	98	=	Refuse to Answer
	99	=	Not Applicable

HCV23.	JUMPBK4
Interviewer: Do you need to jump to a previous section?
If so, click YES to return to the start of the interview, where you can then choose to jump ahead to any section.
Click NO to continue.
JUMPBK4	JUMPBK4. Jump back To a previous section?	1
	0	=	No
	1	=	Yes
	7	=	Don't Know
	8	=	Refuse to Answer
	9	=	Not Applicable

VNT1.	WINDOWE1. In the past 7 days, did you or anyone in your home open window(s) in the same room where someone was cooking?
WINDOWE1	WINDOWE1. In the past 7 days, did you or anyone in your home open window(s) in the same room where someone was cooking?	1
	0	=	No
	1	=	Yes
	7	=	Don't Know
	8	=	Refuse to Answer
	9	=	Not Applicable

VNT2.	WINDOWE2. In the past 7 days, did you or anyone in your home open window(s) in the same room where someone was cleaning (sweeping, dusting, or vacuuming)?
WINDOWE2	WINDOWE2. In the past 7 days, did you or anyone in your home open window(s) in the same room where someone was cleaning (sweeping, dusting, or vacuuming)?	1
	0	=	No
	1	=	Yes
	7	=	Don't Know
	8	=	Refuse to Answer
	9	=	Not Applicable

VNT3.	WINDOWE3. In the past 7 days, did you or any one in your home open window(s) in the same room where someone was smoking?
WINDOWE3	WINDOWE3. In the past 7 days, did you or any one in your home open window(s) in the same room where someone was smoking?	1
	0	=	No
	1	=	Yes
	7	=	Don't Know
	8	=	Refuse to Answer
	9	=	Not Applicable

VNT4.	STANDE3. In the past 7 days, did you or anyone who was smoking in your home stand/sit by an open door/window while they were smoking?
STANDE3	STANDE3. In the past 7 days, did you or anyone who was smoking in your home stand/sit by an open door/window while they were smoking?	1
	0	=	No
	1	=	Yes
	7	=	Don't Know
	8	=	Refuse to Answer
	9	=	Not Applicable

VNT5.	INTERE1. In the past 7 days, did you or anyone in your home close an interior door connected to the room where someone was cooking?
INTERVIEWER: An interior door is one not leading outside the home.
INTERE1	INTERE1. In the past 7 days, did you or anyone in your home close an interior door connected to the room where someone was cooking?	1
	0	=	No
	1	=	Yes
	7	=	Don't Know
	8	=	Refuse to Answer
	9	=	Not Applicable

VNT6.	INTERE2. In the past 7 days, did you or anyone in your home close an interior door connected to the room where someone was cleaning (sweeping, dusting, or vacuuming)?
INTERE2	INTERE2. In the past 7 days, did you or anyone in your home close an interior door connected to the room where someone was cleaning (sweeping, dusting, or vacuuming)?	1
	0	=	No
	1	=	Yes
	7	=	Don't Know
	8	=	Refuse to Answer
	9	=	Not Applicable

VNT7.	INTERE3. In the past 7 days, did you or anyone in your home close an interior door connected to the room where someone was smoking?
INTERE3	INTERE3. In the past 7 days, did you or anyone in your home close an interior door connected to the room where someone was smoking?	1
	0	=	No
	1	=	Yes
	7	=	Don't Know
	8	=	Refuse to Answer
	9	=	Not Applicable

VNT8.	EXTERE1. In the past 7 days, did you or any one in your home open an exterior door while someone was cooking?
INTERVIEWER: An exterior door is one leading outside the home.
EXTERE1	EXTERE1. In the past 7 days, did you or any one in your home open an exterior door while someone was cooking?	1
	0	=	No
	1	=	Yes
	7	=	Don't Know
	8	=	Refuse to Answer
	9	=	Not Applicable

VNT9.	EXTERE2. In the past 7 days, did you or any one in your home open an exterior door while someone was cleaning (sweeping, dusting, or vacuuming)?
EXTERE2	EXTERE2. In the past 7 days, did you or any one in your home open an exterior door while someone was cleaning (sweeping, dusting, or vacuuming)?	1
	0	=	No
	1	=	Yes
	7	=	Don't Know
	8	=	Refuse to Answer
	9	=	Not Applicable

VNT10.	EXTERE3. In the past 7 days, did you or any one in your home open an exterior door while someone was smoking?
EXTERE3	EXTERE3. In the past 7 days, did you or any one in your home open an exterior door while someone was smoking?	1
	0	=	No
	1	=	Yes
	7	=	Don't Know
	8	=	Refuse to Answer
	9	=	Not Applicable

VNT11.	PURIFE1. In the past 7 days, did you or anyone in your home use an air purifier with a fan in the same room where someone was cooking?
INTERVIEWER: Click "Not Applicable" if there is no such device in the home, to skip the next two questions.
PURIFE1	PURIFE1. In the past 7 days, did you or anyone in your home use an air purifier with a fan in the same room where someone was cooking?	1
	0	=	No
	1	=	Yes
	7	=	Don't Know
	8	=	Refuse to Answer
	9	=	Not Applicable

VNT12.	PURIFE2. In the past 7 days, did you or anyone in your home use an air purifier with a fan in the same room where someone was cleaning (sweeping, dusting, or vacuuming)?
PURIFE2	PURIFE2. In the past 7 days, did you or anyone in your home use an air purifier with a fan in the same room where someone was cleaning (sweeping, dusting, or vacuuming)?	1
	0	=	No
	1	=	Yes
	7	=	Don't Know
	8	=	Refuse to Answer
	9	=	Not Applicable

VNT13.	PURIFE3. In the past 7 days, did you or anyone in your home use an air purifier with a fan in the same room where someone was smoking?
PURIFE3	PURIFE3. In the past 7 days, did you or anyone in your home use an air purifier with a fan in the same room where someone was smoking?	1
	0	=	No
	1	=	Yes
	7	=	Don't Know
	8	=	Refuse to Answer
	9	=	Not Applicable

VNT14.	EXHSTE1. In the past 7 days, did you or anyone in your home use an exhaust fan in the same room where someone was cooking?
INTERVIEWER: Click "Not Applicable" if there is no such device in the home, to skip the next two questions.
EXHSTE1	EXHSTE1. In the past 7 days, did you or anyone in your home use an exhaust fan in the same room where someone was cooking?	1
	0	=	No
	1	=	Yes
	7	=	Don't Know
	8	=	Refuse to Answer
	9	=	Not Applicable

VNT15.	EXHSTE2. In the past 7 days, did you or anyone in your home use an exhaust fan in the same room where someone was cleaning (sweeping, dusting, or vacuuming)?
EXHSTE2	EXHSTE2. In the past 7 days, did you or anyone in your home use an exhaust fan in the same room where someone was cleaning (sweeping, dusting, or vacuuming)?	1
	0	=	No
	1	=	Yes
	7	=	Don't Know
	8	=	Refuse to Answer
	9	=	Not Applicable

VNT16.	EXHSTE3. In the past 7 days, did you or anyone in your home use an exhaust fan in the same room where someone was smoking?
EXHSTE3	EXHSTE3. In the past 7 days, did you or anyone in your home use an exhaust fan in the same room where someone was smoking?	1
	0	=	No
	1	=	Yes
	7	=	Don't Know
	8	=	Refuse to Answer
	9	=	Not Applicable

VNT17.	FANE1. In the past 7 days, did you or anyone in your home use a ceiling fan or directional fan in the same room where someone was cooking?
INTERVIEWER: Click "Not Applicable" if there is no such device in the home, to skip the next two questions.
FANE1	FANE1. In the past 7 days, did you or anyone in your home use a ceiling fan or directional fan in the same room where someone was cooking?	1
	0	=	No
	1	=	Yes
	7	=	Don't Know
	8	=	Refuse to Answer
	9	=	Not Applicable

VNT18.	FANE2. In the past 7 days, did you or anyone in your home use a ceiling fan or directional fan in the same room where someone was cleaning (sweeping, dusting, or vacuuming)?
FANE2	FANE2. In the past 7 days, did you or anyone in your home use a ceiling fan or directional fan in the same room where someone was cleaning (sweeping, dusting, or vacuuming)?	1
	0	=	No
	1	=	Yes
	7	=	Don't Know
	8	=	Refuse to Answer
	9	=	Not Applicable

VNT19.	FANE3. In the past 7 days, did you or anyone in your home use a ceiling fan or directional fan in the same room where someone was smoking?
FANE3	FANE3. In the past 7 days, did you or anyone in your home use a ceiling fan or directional fan in the same room where someone was smoking?	1
	0	=	No
	1	=	Yes
	7	=	Don't Know
	8	=	Refuse to Answer
	9	=	Not Applicable

VNT20.	ACWINE1. In the past 7 days, did you or anyone in your home use a window fan or window air conditioner while someone was cooking?
INTERVIEWER: Click "Not Applicable" if there is no such device in the home, to skip the next two questions.
ACWINE1	ACWINE1. In the past 7 days, did you or anyone in your home use a window fan or window air conditioner while someone was cooking?	1
	0	=	No
	1	=	Yes
	7	=	Don't Know
	8	=	Refuse to Answer
	9	=	Not Applicable

VNT21.	ACWINE2. In the past 7 days, did you or any one in your home use a window fan or window air conditioner while someone was cleaning (sweeping, dusting, or vacuuming)?
ACWINE2	ACWINE2. In the past 7 days, did you or any one in your home use a window fan or window air conditioner while someone was cleaning (sweeping, dusting, or vacuuming)?	1
	0	=	No
	1	=	Yes
	7	=	Don't Know
	8	=	Refuse to Answer
	9	=	Not Applicable

VNT22.	ACWINE3. In the past 7 days, did you or any one in your home use a window fan or window air conditioner while someone was smoking?
ACWINE3	ACWINE3. In the past 7 days, did you or any one in your home use a window fan or window air conditioner while someone was smoking?	1
	0	=	No
	1	=	Yes
	7	=	Don't Know
	8	=	Refuse to Answer
	9	=	Not Applicable

VNT23.	CENTE1. In the past 7 days, did you or any one in your home use a central air handling system (heating, cooling, or ventilation) while someone was cooking?
INTERVIEWER: Click "Not Applicable" if there is no such device in the home, to skip the next two questions.
CENTE1	CENTE1. In the past 7 days, did you or any one in your home use a central air handling system (heating, cooling, or ventilation) while someone was cooking?	1
	0	=	No
	1	=	Yes
	7	=	Don't Know
	8	=	Refuse to Answer
	9	=	Not Applicable

VNT24.	CENTE2. In the past 7 days, did you or any one in your home use a central air handling system (heating, cooling, or ventilation) while someone was cleaning (sweeping, dusting, or vacuuming)?
CENTE2	CENTE2. In the past 7 days, did you or any one in your home use a central air handling system (heating, cooling, or ventilation) while someone was cleaning (sweeping, dusting, or vacuuming)?	1
	0	=	No
	1	=	Yes
	7	=	Don't Know
	8	=	Refuse to Answer
	9	=	Not Applicable

VNT25.	CENTE3. In the past 7 days, did you or any one in your home use a central air handling system (heating, cooling, or ventilation) while someone was smoking?
CENTE3	CENTE3. In the past 7 days, did you or any one in your home use a central air handling system (heating, cooling, or ventilation) while someone was smoking?	1
	0	=	No
	1	=	Yes
	7	=	Don't Know
	8	=	Refuse to Answer
	9	=	Not Applicable

VNT26.	LEAVEE3. In the past 7 days, did you or anyone who was smoking in your home leave the house while they were smoking?
LEAVEE3	LEAVEE3. In the past 7 days, did you or anyone who was smoking in your home leave the house while they were smoking?	1
	0	=	No
	1	=	Yes
	7	=	Don't Know
	8	=	Refuse to Answer
	9	=	Not Applicable

VNT27.	JUMPBK5
Interviewer: Do you need to jump to a previous section?
If so, click YES to return to the start of the interview, where you can then choose to jump ahead to any section.
Click NO to continue.
JUMPBK5	JUMPBK5. Jump back to a previous section?	1
	0	=	No
	1	=	Yes
	7	=	Don't Know
	8	=	Refuse to Answer
	9	=	Not Applicable

PGA1.	INCEN7. In the past 7 days, how many days did you or someone in your home burn incense or candles?
INTERVIEWER: Click "Not Applicable" if there is no such item/activity in the home, to skip the next 5 questions.
INCEN7	INCEN7. In the past 7 days, how many days did you or someone in your home burn incense or candles?	5
	0 - 7	=	range
	97	=	Don't Know
	98	=	Refuse to Answer
	99	=	Not Applicable

PGA2.	INCENWH. Where did someone burn incense or candles? 
(Check all that apply)
INTERVIEWER: Use Floor Plan Sketch to prompt respondent.
INCENWHA	INCENWH. Where did someone burn incense or candles? (Check all that apply): LIV = Living Room	1
	0	=	No
	1	=	Yes
	97	=	Don't Know
	98	=	Refuse to Answer
	99	=	Not Applicable

INCENWHB	INCENWH. Where did someone burn incense or candles? (Check all that apply): KIT = Kitchen	1
	0	=	No
	1	=	Yes
	97	=	Don't Know
	98	=	Refuse to Answer
	99	=	Not Applicable

INCENWHC	INCENWH. Where did someone burn incense or candles? (Check all that apply): DIN = Dining Room	1
	0	=	No
	1	=	Yes
	97	=	Don't Know
	98	=	Refuse to Answer
	99	=	Not Applicable

INCENWHD	INCENWH. Where did someone burn incense or candles? (Check all that apply): BED1 = 1st Bedroom	1
	0	=	No
	1	=	Yes
	97	=	Don't Know
	98	=	Refuse to Answer
	99	=	Not Applicable

INCENWHE	INCENWH. Where did someone burn incense or candles? (Check all that apply): BED2 = 2nd Bedroom	1
	0	=	No
	1	=	Yes
	97	=	Don't Know
	98	=	Refuse to Answer
	99	=	Not Applicable

INCENWHF	INCENWH. Where did someone burn incense or candles? (Check all that apply): BED3 = 3rd Bedroom	1
	0	=	No
	1	=	Yes
	97	=	Don't Know
	98	=	Refuse to Answer
	99	=	Not Applicable

INCENWHG	INCENWH. Where did someone burn incense or candles? (Check all that apply): BED4 = 4th Bedroom	1
	0	=	No
	1	=	Yes
	97	=	Don't Know
	98	=	Refuse to Answer
	99	=	Not Applicable

INCENWHH	INCENWH. Where did someone burn incense or candles? (Check all that apply): BATH1 = 1st Bathroom	1
	0	=	No
	1	=	Yes
	97	=	Don't Know
	98	=	Refuse to Answer
	99	=	Not Applicable

INCENWHI	INCENWH. Where did someone burn incense or candles? (Check all that apply): BATH2 = 2nd Bathroom	1
	0	=	No
	1	=	Yes
	97	=	Don't Know
	98	=	Refuse to Answer
	99	=	Not Applicable

INCENWHJ	INCENWH. Where did someone burn incense or candles? (Check all that apply): BATH3 = 3rd Bathroom	1
	0	=	No
	1	=	Yes
	97	=	Don't Know
	98	=	Refuse to Answer
	99	=	Not Applicable

INCENWHK	INCENWH. Where did someone burn incense or candles? (Check all that apply): BAL1 = Balcony1	1
	0	=	No
	1	=	Yes
	97	=	Don't Know
	98	=	Refuse to Answer
	99	=	Not Applicable

INCENWHL	INCENWH. Where did someone burn incense or candles? (Check all that apply): BAL2 = Balcony2	1
	0	=	No
	1	=	Yes
	97	=	Don't Know
	98	=	Refuse to Answer
	99	=	Not Applicable

INCENWHM	INCENWH. Where did someone burn incense or candles? (Check all that apply): PAT = Patio	1
	0	=	No
	1	=	Yes
	97	=	Don't Know
	98	=	Refuse to Answer
	99	=	Not Applicable

INCENWHN	INCENWH. Where did someone burn incense or candles? (Check all that apply): LOB = Lobby	1
	0	=	No
	1	=	Yes
	97	=	Don't Know
	98	=	Refuse to Answer
	99	=	Not Applicable

INCENWHO	INCENWH. Where did someone burn incense or candles? (Check all that apply): HALL1 = Hallway 1	1
	0	=	No
	1	=	Yes
	97	=	Don't Know
	98	=	Refuse to Answer
	99	=	Not Applicable

INCENWHP	INCENWH. Where did someone burn incense or candles? (Check all that apply): HALL2 = Hallway 2	1
	0	=	No
	1	=	Yes
	97	=	Don't Know
	98	=	Refuse to Answer
	99	=	Not Applicable

INCENWHQ	INCENWH. Where did someone burn incense or candles? (Check all that apply): HALL3 = Hallway 3	1
	0	=	No
	1	=	Yes
	97	=	Don't Know
	98	=	Refuse to Answer
	99	=	Not Applicable

INCENWHR	INCENWH. Where did someone burn incense or candles? (Check all that apply): LAUN = Laundry/Utility	1
	0	=	No
	1	=	Yes
	97	=	Don't Know
	98	=	Refuse to Answer
	99	=	Not Applicable

INCENWHS	INCENWH. Where did someone burn incense or candles? (Check all that apply): DEN = Rec/Family Rm	1
	0	=	No
	1	=	Yes
	97	=	Don't Know
	98	=	Refuse to Answer
	99	=	Not Applicable

INCENWHT	INCENWH. Where did someone burn incense or candles? (Check all that apply): OTH1 = Other Room1	1
	0	=	No
	1	=	Yes
	97	=	Don't Know
	98	=	Refuse to Answer
	99	=	Not Applicable

INCENWHU	INCENWH. Where did someone burn incense or candles? (Check all that apply): OTH2 = Other Room2	1
	0	=	No
	1	=	Yes
	97	=	Don't Know
	98	=	Refuse to Answer
	99	=	Not Applicable

INCENWHV	INCENWH. Where did someone burn incense or candles? (Check all that apply): OTH3 = Other Room3	1
	0	=	No
	1	=	Yes
	97	=	Don't Know
	98	=	Refuse to Answer
	99	=	Not Applicable


PGA3.	INCENM. On a typical day in the past 7 days, how many times did someone burn incense or candles in your home during the Morning (6 AM to Noon)?
INCENM	INCENM. On a typical day in the past 7 days, how many times did someone burn incense or candles in your home during the Morning (6 AM to Noon)?	5
	0 - 96	=	range
	97	=	Don't Know
	98	=	Refuse to Answer
	99	=	Not Applicable

PGA4.	INCENA. On a typical day in the past 7 days, how many times did someone burn incense or candles in your home during the Afternoon (Noon to 6PM)?
INCENA	INCENA. On a typical day in the past 7 days, how many times did someone burn incense or candles in your home during the Afternoon (Noon to 6PM)?	5
	0 - 96	=	range
	97	=	Don't Know
	98	=	Refuse to Answer
	99	=	Not Applicable

PGA5.	INCENE. On a typical day in the past 7 days, how many times did someone burn incense or candles in your home during the Evening (6PM to Midnight)?
INCENE	INCENE. On a typical day in the past 7 days, how many times did someone burn incense or candles in your home during the Evening (6PM to Midnight)?	5
	0 - 96	=	range
	97	=	Don't Know
	98	=	Refuse to Answer
	99	=	Not Applicable

PGA6.	INCENN. On a typical day in the past 7 days, how many times did someone burn incense or candles in your home during the Night (Midnight to 6AM)?
INCENN	INCENN. On a typical day in the past 7 days, how many times did someone burn incense or candles in your home during the Night (Midnight to 6AM)?	5
	0 - 96	=	range
	97	=	Don't Know
	98	=	Refuse to Answer
	99	=	Not Applicable

PGA7.	FOOD7. In the past 7 days, how many days did you or someone in your home burn food?
INTERVIEWER: Click "Not Applicable" if there is no such item/activity in the home, to skip the next 5 questions.
FOOD7	FOOD7. In the past 7 days, how many days did you or someone in your home burn food?	5
	0 - 7	=	range
	97	=	Don't Know
	98	=	Refuse to Answer
	99	=	Not Applicable

PGA8.	FOODWH. Where did someone burn food? 
(Check all that apply)
INTERVIEWER: Use Floor Plan Sketch to prompt respondent.
FOODWHA	FOODWH. Where did someone burn food? (Check all that apply): LIV = Living Room	1
	0	=	No
	1	=	Yes
	97	=	Don't Know
	98	=	Refuse to Answer
	99	=	Not Applicable

FOODWHB	FOODWH. Where did someone burn food? (Check all that apply): KIT = Kitchen	1
	0	=	No
	1	=	Yes
	97	=	Don't Know
	98	=	Refuse to Answer
	99	=	Not Applicable

FOODWHC	FOODWH. Where did someone burn food? (Check all that apply): DIN = Dining Room	1
	0	=	No
	1	=	Yes
	97	=	Don't Know
	98	=	Refuse to Answer
	99	=	Not Applicable

FOODWHD	FOODWH. Where did someone burn food? (Check all that apply): BED1 = 1st Bedroom	1
	0	=	No
	1	=	Yes
	97	=	Don't Know
	98	=	Refuse to Answer
	99	=	Not Applicable

FOODWHE	FOODWH. Where did someone burn food? (Check all that apply): BED2 = 2nd Bedroom	1
	0	=	No
	1	=	Yes
	97	=	Don't Know
	98	=	Refuse to Answer
	99	=	Not Applicable

FOODWHF	FOODWH. Where did someone burn food? (Check all that apply): BED3 = 3rd Bedroom	1
	0	=	No
	1	=	Yes
	97	=	Don't Know
	98	=	Refuse to Answer
	99	=	Not Applicable

FOODWHG	FOODWH. Where did someone burn food? (Check all that apply): BED4 = 4th Bedroom	1
	0	=	No
	1	=	Yes
	97	=	Don't Know
	98	=	Refuse to Answer
	99	=	Not Applicable

FOODWHH	FOODWH. Where did someone burn food? (Check all that apply): BATH1 = 1st Bathroom	1
	0	=	No
	1	=	Yes
	97	=	Don't Know
	98	=	Refuse to Answer
	99	=	Not Applicable

FOODWHI	FOODWH. Where did someone burn food? (Check all that apply): BATH2 = 2nd Bathroom	1
	0	=	No
	1	=	Yes
	97	=	Don't Know
	98	=	Refuse to Answer
	99	=	Not Applicable

FOODWHJ	FOODWH. Where did someone burn food? (Check all that apply): BATH3 = 3rd Bathroom	1
	0	=	No
	1	=	Yes
	97	=	Don't Know
	98	=	Refuse to Answer
	99	=	Not Applicable

FOODWHK	FOODWH. Where did someone burn food? (Check all that apply): BAL1 = Balcony1	1
	0	=	No
	1	=	Yes
	97	=	Don't Know
	98	=	Refuse to Answer
	99	=	Not Applicable

FOODWHL	FOODWH. Where did someone burn food? (Check all that apply): BAL2 = Balcony2	1
	0	=	No
	1	=	Yes
	97	=	Don't Know
	98	=	Refuse to Answer
	99	=	Not Applicable

FOODWHM	FOODWH. Where did someone burn food? (Check all that apply): PAT = Patio	1
	0	=	No
	1	=	Yes
	97	=	Don't Know
	98	=	Refuse to Answer
	99	=	Not Applicable

FOODWHN	FOODWH. Where did someone burn food? (Check all that apply): LOB = Lobby	1
	0	=	No
	1	=	Yes
	97	=	Don't Know
	98	=	Refuse to Answer
	99	=	Not Applicable

FOODWHO	FOODWH. Where did someone burn food? (Check all that apply): HALL1 = Hallway 1	1
	0	=	No
	1	=	Yes
	97	=	Don't Know
	98	=	Refuse to Answer
	99	=	Not Applicable

FOODWHP	FOODWH. Where did someone burn food? (Check all that apply): HALL2 = Hallway 2	1
	0	=	No
	1	=	Yes
	97	=	Don't Know
	98	=	Refuse to Answer
	99	=	Not Applicable

FOODWHQ	FOODWH. Where did someone burn food? (Check all that apply): HALL3 = Hallway 3	1
	0	=	No
	1	=	Yes
	97	=	Don't Know
	98	=	Refuse to Answer
	99	=	Not Applicable

FOODWHR	FOODWH. Where did someone burn food? (Check all that apply): LAUN = Laundry/Utility	1
	0	=	No
	1	=	Yes
	97	=	Don't Know
	98	=	Refuse to Answer
	99	=	Not Applicable

FOODWHS	FOODWH. Where did someone burn food? (Check all that apply): DEN = Rec/Family Rm	1
	0	=	No
	1	=	Yes
	97	=	Don't Know
	98	=	Refuse to Answer
	99	=	Not Applicable

FOODWHT	FOODWH. Where did someone burn food? (Check all that apply): OTH1 = Other Room1	1
	0	=	No
	1	=	Yes
	97	=	Don't Know
	98	=	Refuse to Answer
	99	=	Not Applicable

FOODWHU	FOODWH. Where did someone burn food? (Check all that apply): OTH2 = Other Room2	1
	0	=	No
	1	=	Yes
	97	=	Don't Know
	98	=	Refuse to Answer
	99	=	Not Applicable

FOODWHV	FOODWH. Where did someone burn food? (Check all that apply): OTH3 = Other Room3	1
	0	=	No
	1	=	Yes
	97	=	Don't Know
	98	=	Refuse to Answer
	99	=	Not Applicable


PGA9.	FOODM. On a typical day in the past 7 days, how many times did someone burn food in your home during the Morning (6AM to Noon)?
FOODM	FOODM. On a typical day in the past 7 days, how many times did someone burn food in your home during the Morning (6 AM to Noon)?	5
	0 - 96	=	range
	97	=	Don't Know
	98	=	Refuse to Answer
	99	=	Not Applicable

PGA10.	FOODA. On a typical day in the past 7 days, how many times did someone burn food in your home during the Afternoon (Noon to 6PM)?
FOODA	FOODA. On a typical day in the past 7 days, how many times did someone burn food in your home during the Afternoon (Noon to 6PM)?	5
	0 - 96	=	range
	97	=	Don't Know
	98	=	Refuse to Answer
	99	=	Not Applicable

PGA11.	FOODE. On a typical day in the past 7 days, how many times did someone burn food in your home during the Evening (6PM to Midnight)?
FOODE	FOODE. On a typical day in the past 7 days, how many times did someone burn food in your home during the Evening (6PM to Midnight)?	5
	0 - 96	=	range
	97	=	Don't Know
	98	=	Refuse to Answer
	99	=	Not Applicable

PGA12.	FOODN. On a typical day in the past 7 days, how many times did someone burn food in your home during the Night (Midnight to 6AM)?
FOODN	FOODN. On a typical day in the past 7 days, how many times did someone burn food in your home during the Night (Midnight to 6AM)?	5
	0 - 96	=	range
	97	=	Don't Know
	98	=	Refuse to Answer
	99	=	Not Applicable

PGA13.	FRY7. In the past 7 days, how many days did you or someone in your home fry or sauté food with oil or fat?
INTERVIEWER: This includes vegetable oil, olive oil, lard, butter, margarine, "I Can't Believe It's Not Butter!", bacon grease, PAM
Click "Not Applicable" if there is no such item/activity in the home, to skip the next 5 questions.
FRY7	FRY7. In the past 7 days, how many days did you or someone in your home fry or sauté food with oil?	5
	0 - 7	=	range
	97	=	Don't Know
	98	=	Refuse to Answer
	99	=	Not Applicable

PGA14.	FRYWH. Where did someone fry or sauté food with oil or fat?
(Check all that apply)
INTERVIEWER: Use Floor Plan Sketch to prompt respondent.
FRYWHA	FRYWH. Where did someone fry or sauté food with oil or fat? (Check all that apply): LIV = Living Room	1
	0	=	No
	1	=	Yes
	97	=	Don't Know
	98	=	Refuse to Answer
	99	=	Not Applicable

FRYWHB	FRYWH. Where did someone fry or sauté food with oil or fat? (Check all that apply): KIT = Kitchen	1
	0	=	No
	1	=	Yes
	97	=	Don't Know
	98	=	Refuse to Answer
	99	=	Not Applicable

FRYWHC	FRYWH. Where did someone fry or sauté food with oil or fat? (Check all that apply): DIN = Dining Room	1
	0	=	No
	1	=	Yes
	97	=	Don't Know
	98	=	Refuse to Answer
	99	=	Not Applicable

FRYWHD	FRYWH. Where did someone fry or sauté food with oil or fat? (Check all that apply): BED1 = 1st Bedroom	1
	0	=	No
	1	=	Yes
	97	=	Don't Know
	98	=	Refuse to Answer
	99	=	Not Applicable

FRYWHE	FRYWH. Where did someone fry or sauté food with oil or fat? (Check all that apply): BED2 = 2nd Bedroom	1
	0	=	No
	1	=	Yes
	97	=	Don't Know
	98	=	Refuse to Answer
	99	=	Not Applicable

FRYWHF	FRYWH. Where did someone fry or sauté food with oil or fat? (Check all that apply): BED3 = 3rd Bedroom	1
	0	=	No
	1	=	Yes
	97	=	Don't Know
	98	=	Refuse to Answer
	99	=	Not Applicable

FRYWHG	FRYWH. Where did someone fry or sauté food with oil or fat? (Check all that apply): BED4 = 4th Bedroom	1
	0	=	No
	1	=	Yes
	97	=	Don't Know
	98	=	Refuse to Answer
	99	=	Not Applicable

FRYWHH	FRYWH. Where did someone fry or sauté food with oil or fat? (Check all that apply): BATH1 = 1st Bathroom	1
	0	=	No
	1	=	Yes
	97	=	Don't Know
	98	=	Refuse to Answer
	99	=	Not Applicable

FRYWHI	FRYWH. Where did someone fry or sauté food with oil or fat? (Check all that apply): BATH2 = 2nd Bathroom	1
	0	=	No
	1	=	Yes
	97	=	Don't Know
	98	=	Refuse to Answer
	99	=	Not Applicable

FRYWHJ	FRYWH. Where did someone fry or sauté food with oil or fat? (Check all that apply): BATH3 = 3rd Bathroom	1
	0	=	No
	1	=	Yes
	97	=	Don't Know
	98	=	Refuse to Answer
	99	=	Not Applicable

FRYWHK	FRYWH. Where did someone fry or sauté food with oil or fat? (Check all that apply): BAL1 = Balcony1	1
	0	=	No
	1	=	Yes
	97	=	Don't Know
	98	=	Refuse to Answer
	99	=	Not Applicable

FRYWHL	FRYWH. Where did someone fry or sauté food with oil or fat? (Check all that apply): BAL2 = Balcony2	1
	0	=	No
	1	=	Yes
	97	=	Don't Know
	98	=	Refuse to Answer
	99	=	Not Applicable

FRYWHM	FRYWH. Where did someone fry or sauté food with oil or fat? (Check all that apply): PAT = Patio	1
	0	=	No
	1	=	Yes
	97	=	Don't Know
	98	=	Refuse to Answer
	99	=	Not Applicable

FRYWHN	FRYWH. Where did someone fry or sauté food with oil or fat? (Check all that apply): LOB = Lobby	1
	0	=	No
	1	=	Yes
	97	=	Don't Know
	98	=	Refuse to Answer
	99	=	Not Applicable

FRYWHO	FRYWH. Where did someone fry or sauté food with oil or fat? (Check all that apply): HALL1 = Hallway 1	1
	0	=	No
	1	=	Yes
	97	=	Don't Know
	98	=	Refuse to Answer
	99	=	Not Applicable

FRYWHP	FRYWH. Where did someone fry or sauté food with oil or fat? (Check all that apply): HALL2 = Hallway 2	1
	0	=	No
	1	=	Yes
	97	=	Don't Know
	98	=	Refuse to Answer
	99	=	Not Applicable

FRYWHQ	FRYWH. Where did someone fry or sauté food with oil or fat? (Check all that apply): HALL3 = Hallway 3	1
	0	=	No
	1	=	Yes
	97	=	Don't Know
	98	=	Refuse to Answer
	99	=	Not Applicable

FRYWHR	FRYWH. Where did someone fry or sauté food with oil or fat? (Check all that apply): LAUN = Laundry/Utility	1
	0	=	No
	1	=	Yes
	97	=	Don't Know
	98	=	Refuse to Answer
	99	=	Not Applicable

FRYWHS	FRYWH. Where did someone fry or sauté food with oil or fat? (Check all that apply): DEN = Rec/Family Rm	1
	0	=	No
	1	=	Yes
	97	=	Don't Know
	98	=	Refuse to Answer
	99	=	Not Applicable

FRYWHT	FRYWH. Where did someone fry or sauté food with oil or fat? (Check all that apply): OTH1 = Other Room1	1
	0	=	No
	1	=	Yes
	97	=	Don't Know
	98	=	Refuse to Answer
	99	=	Not Applicable

FRYWHU	FRYWH. Where did someone fry or sauté food with oil or fat? (Check all that apply): OTH2 = Other Room2	1
	0	=	No
	1	=	Yes
	97	=	Don't Know
	98	=	Refuse to Answer
	99	=	Not Applicable

FRYWHV	FRYWH. Where did someone fry or sauté food with oil or fat? (Check all that apply): OTH3 = Other Room3	1
	0	=	No
	1	=	Yes
	97	=	Don't Know
	98	=	Refuse to Answer
	99	=	Not Applicable


PGA15.	FRYM. On a typical day in the past 7 days, how many times did someone fry or sauté food with oil or fat in your home during the Morning (6 AM to Noon)?
FRYM	FRYM. On a typical day in the past 7 days, how many times did someone fry or sauté food with oil or fat in your home during the Morning (6 AM to Noon)?	5
	0 - 96	=	range
	97	=	Don't Know
	98	=	Refuse to Answer
	99	=	Not Applicable

PGA16.	FRYA. On a typical day in the past 7 days, how many times did someone fry or sauté food with oil or fat in your home during the Afternoon (Noon to 6PM)?
FRYA	FRYA. On a typical day in the past 7 days, how many times did someone fry or sauté food with oil or fat in your home during the Afternoon (Noon to 6PM)?	5
	0 - 96	=	range
	97	=	Don't Know
	98	=	Refuse to Answer
	99	=	Not Applicable

PGA17.	FRYE. On a typical day in the past 7 days, how many times did someone fry or sauté food with oil or fat in your home during the Evening (6PM to Midnight)?
FRYE	FRYE. On a typical day in the past 7 days, how many times did someone fry or sauté food with  oil or fat in your home during the Evening (6PM to Midnight)?	5
	0 - 96	=	range
	97	=	Don't Know
	98	=	Refuse to Answer
	99	=	Not Applicable

PGA18.	FRYN. On a typical day in the past 7 days, how many times did someone fry or sauté food with oil or fat in your home during the Night (Midnight to 6AM)?
FRYN	FRYN. On a typical day in the past 7 days, how many times did someone fry or sauté food with oil or fat in your home during the Night (Midnight to 6AM)?	5
	0 - 96	=	range
	97	=	Don't Know
	98	=	Refuse to Answer
	99	=	Not Applicable

PGA19.	GAS7. In the past 7 days, how many days did you or someone in your home use a gas/propane appliance to cook or heat food (e.g., stove, portable cooktop, grill, or toaster over)?
INTERVIEWER: Click "Not Applicable" if there is no such item/device in the home, to skip the next 5 questions.
GAS7	GAS7. In the past 7 days, how many days did you or someone in your home use a gas/propane appliance to cook or heat food (e.g., stove, portable cooktop, grill, or toaster over)?	5
	0 - 7	=	range
	97	=	Don't Know
	98	=	Refuse to Answer
	99	=	Not Applicable

PGA20.	GASWH. Where did someone use a gas/propane appliance to cook or heat food (e.g., stove, portable cooktop, grill, or toaster over)? 
(Check all that apply)
INTERVIEWER: Use Floor Plan Sketch to prompt respondent.
GASWHA	GASWH. Where did someone use a gas/propane appliance to cook or heat food (e.g., stove, portable cooktop, grill, or toaster over)? (Check all that apply): LIV = Living Room	1
	0	=	No
	1	=	Yes
	97	=	Don't Know
	98	=	Refuse to Answer
	99	=	Not Applicable

GASWHB	GASWH. Where did someone use a gas/propane appliance to cook or heat food (e.g., stove, portable cooktop, grill, or toaster over)? (Check all that apply): KIT = Kitchen	1
	0	=	No
	1	=	Yes
	97	=	Don't Know
	98	=	Refuse to Answer
	99	=	Not Applicable

GASWHC	GASWH. Where did someone use a gas/propane appliance to cook or heat food (e.g., stove, portable cooktop, grill, or toaster over)? (Check all that apply): DIN = Dining Room	1
	0	=	No
	1	=	Yes
	97	=	Don't Know
	98	=	Refuse to Answer
	99	=	Not Applicable

GASWHD	GASWH. Where did someone use a gas/propane appliance to cook or heat food (e.g., stove, portable cooktop, grill, or toaster over)? (Check all that apply): BED1 = 1st Bedroom	1
	0	=	No
	1	=	Yes
	97	=	Don't Know
	98	=	Refuse to Answer
	99	=	Not Applicable

GASWHE	GASWH. Where did someone use a gas/propane appliance to cook or heat food (e.g., stove, portable cooktop, grill, or toaster over)? (Check all that apply): BED2 = 2nd Bedroom	1
	0	=	No
	1	=	Yes
	97	=	Don't Know
	98	=	Refuse to Answer
	99	=	Not Applicable

GASWHF	GASWH. Where did someone use a gas/propane appliance to cook or heat food (e.g., stove, portable cooktop, grill, or toaster over)? (Check all that apply): BED3 = 3rd Bedroom	1
	0	=	No
	1	=	Yes
	97	=	Don't Know
	98	=	Refuse to Answer
	99	=	Not Applicable

GASWHG	GASWH. Where did someone use a gas/propane appliance to cook or heat food (e.g., stove, portable cooktop, grill, or toaster over)? (Check all that apply): BED4 = 4th Bedroom	1
	0	=	No
	1	=	Yes
	97	=	Don't Know
	98	=	Refuse to Answer
	99	=	Not Applicable

GASWHH	GASWH. Where did someone use a gas/propane appliance to cook or heat food (e.g., stove, portable cooktop, grill, or toaster over)? (Check all that apply): BATH1 = 1st Bathroom	1
	0	=	No
	1	=	Yes
	97	=	Don't Know
	98	=	Refuse to Answer
	99	=	Not Applicable

GASWHI	GASWH. Where did someone use a gas/propane appliance to cook or heat food (e.g., stove, portable cooktop, grill, or toaster over)? (Check all that apply): BATH2 = 2nd Bathroom	1
	0	=	No
	1	=	Yes
	97	=	Don't Know
	98	=	Refuse to Answer
	99	=	Not Applicable

GASWHJ	GASWH. Where did someone use a gas/propane appliance to cook or heat food (e.g., stove, portable cooktop, grill, or toaster over)? (Check all that apply): BATH3 = 3rd Bathroom	1
	0	=	No
	1	=	Yes
	97	=	Don't Know
	98	=	Refuse to Answer
	99	=	Not Applicable

GASWHK	GASWH. Where did someone use a gas/propane appliance to cook or heat food (e.g., stove, portable cooktop, grill, or toaster over)? (Check all that apply): BAL1 = Balcony1	1
	0	=	No
	1	=	Yes
	97	=	Don't Know
	98	=	Refuse to Answer
	99	=	Not Applicable

GASWHL	GASWH. Where did someone use a gas/propane appliance to cook or heat food (e.g., stove, portable cooktop, grill, or toaster over)? (Check all that apply): BAL2 = Balcony2	1
	0	=	No
	1	=	Yes
	97	=	Don't Know
	98	=	Refuse to Answer
	99	=	Not Applicable

GASWHM	GASWH. Where did someone use a gas/propane appliance to cook or heat food (e.g., stove, portable cooktop, grill, or toaster over)? (Check all that apply): PAT = Patio	1
	0	=	No
	1	=	Yes
	97	=	Don't Know
	98	=	Refuse to Answer
	99	=	Not Applicable

GASWHN	GASWH. Where did someone use a gas/propane appliance to cook or heat food (e.g., stove, portable cooktop, grill, or toaster over)? (Check all that apply): LOB = Lobby	1
	0	=	No
	1	=	Yes
	97	=	Don't Know
	98	=	Refuse to Answer
	99	=	Not Applicable

GASWHO	GASWH. Where did someone use a gas/propane appliance to cook or heat food (e.g., stove, portable cooktop, grill, or toaster over)? (Check all that apply): HALL1 = Hallway 1	1
	0	=	No
	1	=	Yes
	97	=	Don't Know
	98	=	Refuse to Answer
	99	=	Not Applicable

GASWHP	GASWH. Where did someone use a gas/propane appliance to cook or heat food (e.g., stove, portable cooktop, grill, or toaster over)? (Check all that apply): HALL2 = Hallway 2	1
	0	=	No
	1	=	Yes
	97	=	Don't Know
	98	=	Refuse to Answer
	99	=	Not Applicable

GASWHQ	GASWH. Where did someone use a gas/propane appliance to cook or heat food (e.g., stove, portable cooktop, grill, or toaster over)? (Check all that apply): HALL3 = Hallway 3	1
	0	=	No
	1	=	Yes
	97	=	Don't Know
	98	=	Refuse to Answer
	99	=	Not Applicable

GASWHR	GASWH. Where did someone use a gas/propane appliance to cook or heat food (e.g., stove, portable cooktop, grill, or toaster over)? (Check all that apply): LAUN = Laundry/Utility	1
	0	=	No
	1	=	Yes
	97	=	Don't Know
	98	=	Refuse to Answer
	99	=	Not Applicable

GASWHS	GASWH. Where did someone use a gas/propane appliance to cook or heat food (e.g., stove, portable cooktop, grill, or toaster over)? (Check all that apply): DEN = Rec/Family Rm	1
	0	=	No
	1	=	Yes
	97	=	Don't Know
	98	=	Refuse to Answer
	99	=	Not Applicable

GASWHT	GASWH. Where did someone use a gas/propane appliance to cook or heat food (e.g., stove, portable cooktop, grill, or toaster over)? (Check all that apply): OTH1 = Other Room1	1
	0	=	No
	1	=	Yes
	97	=	Don't Know
	98	=	Refuse to Answer
	99	=	Not Applicable

GASWHU	GASWH. Where did someone use a gas/propane appliance to cook or heat food (e.g., stove, portable cooktop, grill, or toaster over)? (Check all that apply): OTH2 = Other Room2	1
	0	=	No
	1	=	Yes
	97	=	Don't Know
	98	=	Refuse to Answer
	99	=	Not Applicable

GASWHV	GASWH. Where did someone use a gas/propane appliance to cook or heat food (e.g., stove, portable cooktop, grill, or toaster over)? (Check all that apply): OTH3 = Other Room3	1
	0	=	No
	1	=	Yes
	97	=	Don't Know
	98	=	Refuse to Answer
	99	=	Not Applicable


PGA21.	GASM. On a typical day in the past 7 days, how many times did someone use a gas/propane appliance to cook or heat food (e.g., stove, portable cooktop, grill, or toaster over) in your home during the Morning (6 AM to Noon)?
GASM	GASM. On a typical day in the past 7 days, how many times did someone use a gas/propane appliance to cook or heat food (e.g., stove, portable cooktop, grill, or toaster over) in your home during the Morning (6 AM to Noon)?	5
	0 - 96	=	range
	97	=	Don't Know
	98	=	Refuse to Answer
	99	=	Not Applicable

PGA22.	GASA. On a typical day in the past 7 days, how many times did someone use a gas/propane appliance to cook or heat food (e.g., stove, portable cooktop, grill, or toaster over) in your home during the Afternoon (Noon to 6PM)?
GASA	GASA. On a typical day in the past 7 days, how many times did someone use a gas/propane appliance to cook or heat food (e.g., stove, portable cooktop, grill, or toaster over) in your home during the Afternoon (Noon to 6PM)?	5
	0 - 96	=	range
	97	=	Don't Know
	98	=	Refuse to Answer
	99	=	Not Applicable

PGA23.	GASE. On a typical day in the past 7 days, how many times did someone use a gas/propane appliance to cook or heat food (e.g., stove, portable cooktop, grill, or toaster over) in your home during the Evening (6PM to Midnight)?
GASE	GASE. On a typical day in the past 7 days, how many times did someone use a gas/propane appliance to cook or heat food (e.g., stove, portable cooktop, grill, or toaster over) in your home during the Evening (6PM to Midnight)?	5
	0 - 96	=	range
	97	=	Don't Know
	98	=	Refuse to Answer
	99	=	Not Applicable

PGA24.	GASN. On a typical day in the past 7 days, how many times did someone use a gas/propane appliance to cook or heat food (e.g., stove, portable cooktop, grill, or toaster over) in your home during the Night (Midnight to 6AM)?
GASN	GASN. On a typical day in the past 7 days, how many times did someone use a gas/propane appliance to cook or heat food (e.g., stove, portable cooktop, grill, or toaster over) in your home during the Night (Midnight to 6AM)?	5
	0 - 96	=	range
	97	=	Don't Know
	98	=	Refuse to Answer
	99	=	Not Applicable

PGA25.	ELEC7. In the past 7 days, how many days did you or someone in your home use an electric appliance to cook or heat food (e.g., stove, or portable cooktop, microwave, grill or toaster oven)?
INTERVIEWER: Click "Not Applicable" if there is no such item/device in the home, to skip the next 5 questions.
ELEC7	ELEC7. In the past 7 days, how many days did you or someone in your home use an electric appliance to cook or heat food (e.g., stove, or portable cooktop, microwave, grill or toaster oven)?	5
	0 - 7	=	range
	97	=	Don't Know
	98	=	Refuse to Answer
	99	=	Not Applicable

PGA26.	ELECWH. Where did someone use an electric appliance to cook or heat food (e.g., stove, or portable cooktop, microwave, grill or toaster oven)? 
(Check all that apply)
INTERVIEWER: Use Floor Plan Sketch to prompt respondent.
ELECWHA	ELECWH. Where did someone use an electric appliance to cook or heat food (e.g., stove, or portable cooktop, microwave, grill or toaster oven)? (Check all that apply): LIV = Living Room	1
	0	=	No
	1	=	Yes
	97	=	Don't Know
	98	=	Refuse to Answer
	99	=	Not Applicable

ELECWHB	ELECWH. Where did someone use an electric appliance to cook or heat food (e.g., stove, or portable cooktop, microwave, grill or toaster oven)? (Check all that apply): KIT = Kitchen	1
	0	=	No
	1	=	Yes
	97	=	Don't Know
	98	=	Refuse to Answer
	99	=	Not Applicable

ELECWHC	ELECWH. Where did someone use an electric appliance to cook or heat food (e.g., stove, or portable cooktop, microwave, grill or toaster oven)? (Check all that apply): DIN = Dining Room	1
	0	=	No
	1	=	Yes
	97	=	Don't Know
	98	=	Refuse to Answer
	99	=	Not Applicable

ELECWHD	ELECWH. Where did someone use an electric appliance to cook or heat food (e.g., stove, or portable cooktop, microwave, grill or toaster oven)? (Check all that apply): BED1 = 1st Bedroom	1
	0	=	No
	1	=	Yes
	97	=	Don't Know
	98	=	Refuse to Answer
	99	=	Not Applicable

ELECWHE	ELECWH. Where did someone use an electric appliance to cook or heat food (e.g., stove, or portable cooktop, microwave, grill or toaster oven)? (Check all that apply): BED2 = 2nd Bedroom	1
	0	=	No
	1	=	Yes
	97	=	Don't Know
	98	=	Refuse to Answer
	99	=	Not Applicable

ELECWHF	ELECWH. Where did someone use an electric appliance to cook or heat food (e.g., stove, or portable cooktop, microwave, grill or toaster oven)? (Check all that apply): BED3 = 3rd Bedroom	1
	0	=	No
	1	=	Yes
	97	=	Don't Know
	98	=	Refuse to Answer
	99	=	Not Applicable

ELECWHG	ELECWH. Where did someone use an electric appliance to cook or heat food (e.g., stove, or portable cooktop, microwave, grill or toaster oven)? (Check all that apply): BED4 = 4th Bedroom	1
	0	=	No
	1	=	Yes
	97	=	Don't Know
	98	=	Refuse to Answer
	99	=	Not Applicable

ELECWHH	ELECWH. Where did someone use an electric appliance to cook or heat food (e.g., stove, or portable cooktop, microwave, grill or toaster oven)? (Check all that apply): BATH1 = 1st Bathroom	1
	0	=	No
	1	=	Yes
	97	=	Don't Know
	98	=	Refuse to Answer
	99	=	Not Applicable

ELECWHI	ELECWH. Where did someone use an electric appliance to cook or heat food (e.g., stove, or portable cooktop, microwave, grill or toaster oven)? (Check all that apply): BATH2 = 2nd Bathroom	1
	0	=	No
	1	=	Yes
	97	=	Don't Know
	98	=	Refuse to Answer
	99	=	Not Applicable

ELECWHJ	ELECWH. Where did someone use an electric appliance to cook or heat food (e.g., stove, or portable cooktop, microwave, grill or toaster oven)? (Check all that apply): BATH3 = 3rd Bathroom	1
	0	=	No
	1	=	Yes
	97	=	Don't Know
	98	=	Refuse to Answer
	99	=	Not Applicable

ELECWHK	ELECWH. Where did someone use an electric appliance to cook or heat food (e.g., stove, or portable cooktop, microwave, grill or toaster oven)? (Check all that apply): BAL1 = Balcony1	1
	0	=	No
	1	=	Yes
	97	=	Don't Know
	98	=	Refuse to Answer
	99	=	Not Applicable

ELECWHL	ELECWH. Where did someone use an electric appliance to cook or heat food (e.g., stove, or portable cooktop, microwave, grill or toaster oven)? (Check all that apply): BAL2 = Balcony2	1
	0	=	No
	1	=	Yes
	97	=	Don't Know
	98	=	Refuse to Answer
	99	=	Not Applicable

ELECWHM	ELECWH. Where did someone use an electric appliance to cook or heat food (e.g., stove, or portable cooktop, microwave, grill or toaster oven)? (Check all that apply): PAT = Patio	1
	0	=	No
	1	=	Yes
	97	=	Don't Know
	98	=	Refuse to Answer
	99	=	Not Applicable

ELECWHN	ELECWH. Where did someone use an electric appliance to cook or heat food (e.g., stove, or portable cooktop, microwave, grill or toaster oven)? (Check all that apply): LOB = Lobby	1
	0	=	No
	1	=	Yes
	97	=	Don't Know
	98	=	Refuse to Answer
	99	=	Not Applicable

ELECWHO	ELECWH. Where did someone use an electric appliance to cook or heat food (e.g., stove, or portable cooktop, microwave, grill or toaster oven)? (Check all that apply): HALL1 = Hallway 1	1
	0	=	No
	1	=	Yes
	97	=	Don't Know
	98	=	Refuse to Answer
	99	=	Not Applicable

ELECWHP	ELECWH. Where did someone use an electric appliance to cook or heat food (e.g., stove, or portable cooktop, microwave, grill or toaster oven)? (Check all that apply): HALL2 = Hallway 2	1
	0	=	No
	1	=	Yes
	97	=	Don't Know
	98	=	Refuse to Answer
	99	=	Not Applicable

ELECWHQ	ELECWH. Where did someone use an electric appliance to cook or heat food (e.g., stove, or portable cooktop, microwave, grill or toaster oven)? (Check all that apply): HALL3 = Hallway 3	1
	0	=	No
	1	=	Yes
	97	=	Don't Know
	98	=	Refuse to Answer
	99	=	Not Applicable

ELECWHR	ELECWH. Where did someone use an electric appliance to cook or heat food (e.g., stove, or portable cooktop, microwave, grill or toaster oven)? (Check all that apply): LAUN = Laundry/Utility	1
	0	=	No
	1	=	Yes
	97	=	Don't Know
	98	=	Refuse to Answer
	99	=	Not Applicable

ELECWHS	ELECWH. Where did someone use an electric appliance to cook or heat food (e.g., stove, or portable cooktop, microwave, grill or toaster oven)? (Check all that apply): DEN = Rec/Family Rm	1
	0	=	No
	1	=	Yes
	97	=	Don't Know
	98	=	Refuse to Answer
	99	=	Not Applicable

ELECWHT	ELECWH. Where did someone use an electric appliance to cook or heat food (e.g., stove, or portable cooktop, microwave, grill or toaster oven)? (Check all that apply): OTH1 = Other Room1	1
	0	=	No
	1	=	Yes
	97	=	Don't Know
	98	=	Refuse to Answer
	99	=	Not Applicable

ELECWHU	ELECWH. Where did someone use an electric appliance to cook or heat food (e.g., stove, or portable cooktop, microwave, grill or toaster oven)? (Check all that apply): OTH2 = Other Room2	1
	0	=	No
	1	=	Yes
	97	=	Don't Know
	98	=	Refuse to Answer
	99	=	Not Applicable

ELECWHV	ELECWH. Where did someone use an electric appliance to cook or heat food (e.g., stove, or portable cooktop, microwave, grill or toaster oven)? (Check all that apply): OTH3 = Other Room3	1
	0	=	No
	1	=	Yes
	97	=	Don't Know
	98	=	Refuse to Answer
	99	=	Not Applicable


PGA27.	ELECM. On a typical day in the past 7 days, how many times did someone use an electric appliance to cook or heat food (e.g., stove, or portable cooktop, microwave, grill or toaster oven) in your home during the Morning (6 AM to Noon)?
ELECM	ELECM. On a typical day in the past 7 days, how many times did someone use an electric appliance to cook or heat food (e.g., stove, or portable cooktop, microwave, grill or toaster oven) in your home during the Morning (6 AM to Noon)?	5
	0 - 96	=	range
	97	=	Don't Know
	98	=	Refuse to Answer
	99	=	Not Applicable

PGA28.	ELECA. On a typical day in the past 7 days, how many times did someone use an electric appliance to cook or heat food (e.g., stove, or portable cooktop, microwave, grill or toaster oven) in your home during the Afternoon (Noon to 6PM)?
ELECA	ELECA. On a typical day in the past 7 days, how many times did someone use an electric appliance to cook or heat food (e.g., stove, or portable cooktop, microwave, grill or toaster oven) in your home during the Afternoon (Noon to 6PM)?	5
	0 - 96	=	range
	97	=	Don't Know
	98	=	Refuse to Answer
	99	=	Not Applicable

PGA29.	ELECE. On a typical day in the past 7 days, how many times did someone use an electric appliance to cook or heat food (e.g., stove, or portable cooktop, microwave, grill or toaster oven) in your home during the Evening (6PM to Midnight)?
ELECE	ELECE. On a typical day in the past 7 days, how many times did someone use an electric appliance to cook or heat food (e.g., stove, or portable cooktop, microwave, grill or toaster oven) in your home during the Evening (6PM to Midnight)?	5
	0 - 96	=	range
	97	=	Don't Know
	98	=	Refuse to Answer
	99	=	Not Applicable

PGA30.	ELECN. On a typical day in the past 7 days, how many times did someone use an electric appliance to cook or heat food (e.g., stove, or portable cooktop, microwave, grill or toaster oven) in your home during the Night (Midnight to 6AM)?
ELECN	ELECN. On a typical day in the past 7 days, how many times did someone use an electric appliance to cook or heat food (e.g., stove, or portable cooktop, microwave, grill or toaster oven) in your home during the Night (Midnight to 6AM)?	5
	0 - 96	=	range
	97	=	Don't Know
	98	=	Refuse to Answer
	99	=	Not Applicable

PGA31.	AERO7. In the past 7 days, how many days did you or someone in your home use aerosol spray products (e.g., hair spray, bug spray, deodorant)?
INTERVIEWER: Click "Not Applicable" if there is no such item in the home, to skip the next 5 questions.
AERO7	AERO7. In the past 7 days, how many days did you or someone in your home use aerosol spray products (e.g., hair spray, bug spray, deodorant)?	5
	0 - 7	=	range
	97	=	Don't Know
	98	=	Refuse to Answer
	99	=	Not Applicable

PGA32.	AEROWH. Where did someone use aerosol spray products (e.g., hair spray, bug spray, deodorant)?
(Check all that apply)
INTERVIEWER: Use Floor Plan Sketch to prompt respondent.
AEROWHA	AEROWH. Where did someone use aerosol spray products (e.g., hair spray, bug spray, deodorant)? (Check all that apply): LIV = Living Room	1
	0	=	No
	1	=	Yes
	97	=	Don't Know
	98	=	Refuse to Answer
	99	=	Not Applicable

AEROWHB	AEROWH. Where did someone use aerosol spray products (e.g., hair spray, bug spray, deodorant)? (Check all that apply): KIT = Kitchen	1
	0	=	No
	1	=	Yes
	97	=	Don't Know
	98	=	Refuse to Answer
	99	=	Not Applicable

AEROWHC	AEROWH. Where did someone use aerosol spray products (e.g., hair spray, bug spray, deodorant)? (Check all that apply): DIN = Dining Room	1
	0	=	No
	1	=	Yes
	97	=	Don't Know
	98	=	Refuse to Answer
	99	=	Not Applicable

AEROWHD	AEROWH. Where did someone use aerosol spray products (e.g., hair spray, bug spray, deodorant)? (Check all that apply): BED1 = 1st Bedroom	1
	0	=	No
	1	=	Yes
	97	=	Don't Know
	98	=	Refuse to Answer
	99	=	Not Applicable

AEROWHE	AEROWH. Where did someone use aerosol spray products (e.g., hair spray, bug spray, deodorant)? (Check all that apply): BED2 = 2nd Bedroom	1
	0	=	No
	1	=	Yes
	97	=	Don't Know
	98	=	Refuse to Answer
	99	=	Not Applicable

AEROWHF	AEROWH. Where did someone use aerosol spray products (e.g., hair spray, bug spray, deodorant)? (Check all that apply): BED3 = 3rd Bedroom	1
	0	=	No
	1	=	Yes
	97	=	Don't Know
	98	=	Refuse to Answer
	99	=	Not Applicable

AEROWHG	AEROWH. Where did someone use aerosol spray products (e.g., hair spray, bug spray, deodorant)? (Check all that apply): BED4 = 4th Bedroom	1
	0	=	No
	1	=	Yes
	97	=	Don't Know
	98	=	Refuse to Answer
	99	=	Not Applicable

AEROWHH	AEROWH. Where did someone use aerosol spray products (e.g., hair spray, bug spray, deodorant)? (Check all that apply): BATH1 = 1st Bathroom	1
	0	=	No
	1	=	Yes
	97	=	Don't Know
	98	=	Refuse to Answer
	99	=	Not Applicable

AEROWHI	AEROWH. Where did someone use aerosol spray products (e.g., hair spray, bug spray, deodorant)? (Check all that apply): BATH2 = 2nd Bathroom	1
	0	=	No
	1	=	Yes
	97	=	Don't Know
	98	=	Refuse to Answer
	99	=	Not Applicable

AEROWHJ	AEROWH. Where did someone use aerosol spray products (e.g., hair spray, bug spray, deodorant)? (Check all that apply): BATH3 = 3rd Bathroom	1
	0	=	No
	1	=	Yes
	97	=	Don't Know
	98	=	Refuse to Answer
	99	=	Not Applicable

AEROWHK	AEROWH. Where did someone use aerosol spray products (e.g., hair spray, bug spray, deodorant)? (Check all that apply): BAL1 = Balcony1	1
	0	=	No
	1	=	Yes
	97	=	Don't Know
	98	=	Refuse to Answer
	99	=	Not Applicable

AEROWHL	AEROWH. Where did someone use aerosol spray products (e.g., hair spray, bug spray, deodorant)? (Check all that apply): BAL2 = Balcony2	1
	0	=	No
	1	=	Yes
	97	=	Don't Know
	98	=	Refuse to Answer
	99	=	Not Applicable

AEROWHM	AEROWH. Where did someone use aerosol spray products (e.g., hair spray, bug spray, deodorant)? (Check all that apply): PAT = Patio	1
	0	=	No
	1	=	Yes
	97	=	Don't Know
	98	=	Refuse to Answer
	99	=	Not Applicable

AEROWHN	AEROWH. Where did someone use aerosol spray products (e.g., hair spray, bug spray, deodorant)? (Check all that apply): LOB = Lobby	1
	0	=	No
	1	=	Yes
	97	=	Don't Know
	98	=	Refuse to Answer
	99	=	Not Applicable

AEROWHO	AEROWH. Where did someone use aerosol spray products (e.g., hair spray, bug spray, deodorant)? (Check all that apply): HALL1 = Hallway 1	1
	0	=	No
	1	=	Yes
	97	=	Don't Know
	98	=	Refuse to Answer
	99	=	Not Applicable

AEROWHP	AEROWH. Where did someone use aerosol spray products (e.g., hair spray, bug spray, deodorant)? (Check all that apply): HALL2 = Hallway 2	1
	0	=	No
	1	=	Yes
	97	=	Don't Know
	98	=	Refuse to Answer
	99	=	Not Applicable

AEROWHQ	AEROWH. Where did someone use aerosol spray products (e.g., hair spray, bug spray, deodorant)? (Check all that apply): HALL3 = Hallway 3	1
	0	=	No
	1	=	Yes
	97	=	Don't Know
	98	=	Refuse to Answer
	99	=	Not Applicable

AEROWHR	AEROWH. Where did someone use aerosol spray products (e.g., hair spray, bug spray, deodorant)? (Check all that apply): LAUN = Laundry/Utility	1
	0	=	No
	1	=	Yes
	97	=	Don't Know
	98	=	Refuse to Answer
	99	=	Not Applicable

AEROWHS	AEROWH. Where did someone use aerosol spray products (e.g., hair spray, bug spray, deodorant)? (Check all that apply): DEN = Rec/Family Rm	1
	0	=	No
	1	=	Yes
	97	=	Don't Know
	98	=	Refuse to Answer
	99	=	Not Applicable

AEROWHT	AEROWH. Where did someone use aerosol spray products (e.g., hair spray, bug spray, deodorant)? (Check all that apply): OTH1 = Other Room1	1
	0	=	No
	1	=	Yes
	97	=	Don't Know
	98	=	Refuse to Answer
	99	=	Not Applicable

AEROWHU	AEROWH. Where did someone use aerosol spray products (e.g., hair spray, bug spray, deodorant)? (Check all that apply): OTH2 = Other Room2	1
	0	=	No
	1	=	Yes
	97	=	Don't Know
	98	=	Refuse to Answer
	99	=	Not Applicable

AEROWHV	AEROWH. Where did someone use aerosol spray products (e.g., hair spray, bug spray, deodorant)? (Check all that apply): OTH3 = Other Room3	1
	0	=	No
	1	=	Yes
	97	=	Don't Know
	98	=	Refuse to Answer
	99	=	Not Applicable


PGA33.	AEROM. On a typical day in the past 7 days, how many times did someone use aerosol spray products (e.g., hair spray, bug spray, deodorant) in your home during the Morning (6 AM to Noon)?
AEROM	AEROM. On a typical day in the past 7 days, how many times did someone use aerosol spray products (e.g., hair spray, bug spray, deodorant) in your home during the Morning (6 AM to Noon)?	5
	0 - 96	=	range
	97	=	Don't Know
	98	=	Refuse to Answer
	99	=	Not Applicable

PGA34.	AEROA. On a typical day in the past 7 days, how many times did someone use aerosol spray products (e.g., hair spray, bug spray, deodorant) in your home during the Afternoon (Noon to 6PM)?
AEROA	AEROA. On a typical day in the past 7 days, how many times did someone use aerosol spray products (e.g., hair spray, bug spray, deodorant) in your home during the Afternoon (Noon to 6PM)?	5
	0 - 96	=	range
	97	=	Don't Know
	98	=	Refuse to Answer
	99	=	Not Applicable

PGA35.	AEROE. On a typical day in the past 7 days, how many times did someone use aerosol spray products (e.g., hair spray, bug spray, deodorant) in your home during the Evening (6PM to Midnight)?
AEROE	AEROE. On a typical day in the past 7 days, how many times did someone use aerosol spray products (e.g., hair spray, bug spray, deodorant) in your home during the Evening (6PM to Midnight)?	5
	0 - 96	=	range
	97	=	Don't Know
	98	=	Refuse to Answer
	99	=	Not Applicable

PGA36.	AERON. On a typical day in the past 7 days, how many times did someone use aerosol spray products (e.g., hair spray, bug spray, deodorant) in your home during the Night (Midnight to 6AM)?
AERON	AERON. On a typical day in the past 7 days, how many times did someone use aerosol spray products (e.g., hair spray, bug spray, deodorant) in your home during the Night (Midnight to 6AM)?	5
	0 - 96	=	range
	97	=	Don't Know
	98	=	Refuse to Answer
	99	=	Not Applicable

PGA37.	DUST7. In the past 7 days, how many days did you or someone in your home vacuum/dust/sweep?
INTERVIEWER: Click "Not Applicable" if there is no such item/activity in the home, to skip the next 5 questions.
DUST7	DUST7. In the past 7 days, how many days did you or someone in your home vacuum/dust/sweep?	5
	0 - 7	=	range
	97	=	Don't Know
	98	=	Refuse to Answer
	99	=	Not Applicable

PGA38.	DUSTWH. Where did someone vacuum/dust/sweep? 
(Check all that apply)
INTERVIEWER: Use Floor Plan Sketch to prompt respondent.
DUSTWHA	DUSTWH. Where did someone vacuum/dust/sweep?: LIV = Living Room	1
	0	=	No
	1	=	Yes
	97	=	Don't Know
	98	=	Refuse to Answer
	99	=	Not Applicable

DUSTWHB	DUSTWH. Where did someone vacuum/dust/sweep?: KIT = Kitchen	1
	0	=	No
	1	=	Yes
	97	=	Don't Know
	98	=	Refuse to Answer
	99	=	Not Applicable

DUSTWHC	DUSTWH. Where did someone vacuum/dust/sweep?: DIN = Dining Room	1
	0	=	No
	1	=	Yes
	97	=	Don't Know
	98	=	Refuse to Answer
	99	=	Not Applicable

DUSTWHD	DUSTWH. Where did someone vacuum/dust/sweep?: BED1 = 1st Bedroom	1
	0	=	No
	1	=	Yes
	97	=	Don't Know
	98	=	Refuse to Answer
	99	=	Not Applicable

DUSTWHE	DUSTWH. Where did someone vacuum/dust/sweep?: BED2 = 2nd Bedroom	1
	0	=	No
	1	=	Yes
	97	=	Don't Know
	98	=	Refuse to Answer
	99	=	Not Applicable

DUSTWHF	DUSTWH. Where did someone vacuum/dust/sweep?: BED3 = 3rd Bedroom	1
	0	=	No
	1	=	Yes
	97	=	Don't Know
	98	=	Refuse to Answer
	99	=	Not Applicable

DUSTWHG	DUSTWH. Where did someone vacuum/dust/sweep?: BED4 = 4th Bedroom	1
	0	=	No
	1	=	Yes
	97	=	Don't Know
	98	=	Refuse to Answer
	99	=	Not Applicable

DUSTWHH	DUSTWH. Where did someone vacuum/dust/sweep?: BATH1 = 1st Bathroom	1
	0	=	No
	1	=	Yes
	97	=	Don't Know
	98	=	Refuse to Answer
	99	=	Not Applicable

DUSTWHI	DUSTWH. Where did someone vacuum/dust/sweep?: BATH2 = 2nd Bathroom	1
	0	=	No
	1	=	Yes
	97	=	Don't Know
	98	=	Refuse to Answer
	99	=	Not Applicable

DUSTWHJ	DUSTWH. Where did someone vacuum/dust/sweep?: BATH3 = 3rd Bathroom	1
	0	=	No
	1	=	Yes
	97	=	Don't Know
	98	=	Refuse to Answer
	99	=	Not Applicable

DUSTWHK	DUSTWH. Where did someone vacuum/dust/sweep?: BAL1 = Balcony1	1
	0	=	No
	1	=	Yes
	97	=	Don't Know
	98	=	Refuse to Answer
	99	=	Not Applicable

DUSTWHL	DUSTWH. Where did someone vacuum/dust/sweep?: BAL2 = Balcony2	1
	0	=	No
	1	=	Yes
	97	=	Don't Know
	98	=	Refuse to Answer
	99	=	Not Applicable

DUSTWHM	DUSTWH. Where did someone vacuum/dust/sweep?: PAT = Patio	1
	0	=	No
	1	=	Yes
	97	=	Don't Know
	98	=	Refuse to Answer
	99	=	Not Applicable

DUSTWHN	DUSTWH. Where did someone vacuum/dust/sweep?: LOB = Lobby	1
	0	=	No
	1	=	Yes
	97	=	Don't Know
	98	=	Refuse to Answer
	99	=	Not Applicable

DUSTWHO	DUSTWH. Where did someone vacuum/dust/sweep?: HALL1 = Hallway 1	1
	0	=	No
	1	=	Yes
	97	=	Don't Know
	98	=	Refuse to Answer
	99	=	Not Applicable

DUSTWHP	DUSTWH. Where did someone vacuum/dust/sweep?: HALL2 = Hallway 2	1
	0	=	No
	1	=	Yes
	97	=	Don't Know
	98	=	Refuse to Answer
	99	=	Not Applicable

DUSTWHQ	DUSTWH. Where did someone vacuum/dust/sweep?: HALL3 = Hallway 3	1
	0	=	No
	1	=	Yes
	97	=	Don't Know
	98	=	Refuse to Answer
	99	=	Not Applicable

DUSTWHR	DUSTWH. Where did someone vacuum/dust/sweep?: LAUN = Laundry/Utility	1
	0	=	No
	1	=	Yes
	97	=	Don't Know
	98	=	Refuse to Answer
	99	=	Not Applicable

DUSTWHS	DUSTWH. Where did someone vacuum/dust/sweep?: DEN = Rec/Family Rm	1
	0	=	No
	1	=	Yes
	97	=	Don't Know
	98	=	Refuse to Answer
	99	=	Not Applicable

DUSTWHT	DUSTWH. Where did someone vacuum/dust/sweep?: OTH1 = Other Room1	1
	0	=	No
	1	=	Yes
	97	=	Don't Know
	98	=	Refuse to Answer
	99	=	Not Applicable

DUSTWHU	DUSTWH. Where did someone vacuum/dust/sweep?: OTH2 = Other Room2	1
	0	=	No
	1	=	Yes
	97	=	Don't Know
	98	=	Refuse to Answer
	99	=	Not Applicable

DUSTWHV	DUSTWH. Where did someone vacuum/dust/sweep?: OTH3 = Other Room3	1
	0	=	No
	1	=	Yes
	97	=	Don't Know
	98	=	Refuse to Answer
	99	=	Not Applicable


PGA39.	DUSTM. On a typical day in the past 7 days, how many times did someone vacuum/dust/sweep in your home during the Morning (6 AM to Noon)?
DUSTM	DUSTM. On a typical day in the past 7 days, how many times did someone vacuum/dust/sweep in your home during the Morning (6 AM to Noon)?	5
	0 - 96	=	range
	97	=	Don't Know
	98	=	Refuse to Answer
	99	=	Not Applicable

PGA40.	DUSTA. On a typical day in the past 7 days, how many times did someone vacuum/dust/sweep in your home during the Afternoon (Noon to 6PM)?
DUSTA	DUSTA. On a typical day in the past 7 days, how many times did someone vacuum/dust/sweep in your home during the Afternoon (Noon to 6PM)?	5
	0 - 96	=	range
	97	=	Don't Know
	98	=	Refuse to Answer
	99	=	Not Applicable

PGA41.	DUSTE. On a typical day in the past 7 days, how many times did someone vacuum/dust/sweep in your home during the Evening (6PM to Midnight)?
DUSTE	DUSTE. On a typical day in the past 7 days, how many times did someone vacuum/dust/sweep in your home during the Evening (6PM to Midnight)?	5
	0 - 96	=	range
	97	=	Don't Know
	98	=	Refuse to Answer
	99	=	Not Applicable

PGA42.	DUSTN. On a typical day in the past 7 days, how many times did someone vacuum/dust/sweep in your home during the Night (Midnight to 6AM)?
DUSTN	DUSTN. On a typical day in the past 7 days, how many times did someone vacuum/dust/sweep in your home during the Night (Midnight to 6AM)?	5
	0 - 96	=	range
	97	=	Don't Know
	98	=	Refuse to Answer
	99	=	Not Applicable

PGA43.	APGA7. In the past 7 days, how many days did you or someone in your home do anything else that generates smoke, dust or particles? (specify)?
INTERVIEWER: Click "Not Applicable" if there is no such activity in the home, to skip the next 5 questions.
Examples to prompt the respondent:
·renovating (drywall, demolition, painting) producing dust, fumes, etc.
·started fire in fireplace or wood stove
·noticed pollen/spores coming inside
·sanding or wood work producing dust
·anything producing vapor, steam, droplets
·welding or work producing smoke, dust
APGA7	APGA7. In the past 7 days, how many days did you or someone in your home do anything else that generates smoke, dust or particles? (specify)?	5
	0 - 7	=	range
	97	=	Don't Know
	98	=	Refuse to Answer
	99	=	Not Applicable

PGA44.	APGA7SP. Specify Other particle generating item/activity
APGA7SP	APGA7SP. Specify Other particle generating item/activity	20

PGA45.	APGAWH. Where did someone do anything else that generates smoke, dust or particles?
(Check all that apply)
INTERVIEWER: Use Floor Plan Sketch to prompt respondent.
APGAWHA	APGAWH. Where did someone do anything else that generates smoke, dust or particles?: LIV = Living Room	1
	0	=	No
	1	=	Yes
	97	=	Don't Know
	98	=	Refuse to Answer
	99	=	Not Applicable

APGAWHB	APGAWH. Where did someone do anything else that generates smoke, dust or particles?: KIT = Kitchen	1
	0	=	No
	1	=	Yes
	97	=	Don't Know
	98	=	Refuse to Answer
	99	=	Not Applicable

APGAWHC	APGAWH. Where did someone do anything else that generates smoke, dust or particles?: DIN = Dining Room	1
	0	=	No
	1	=	Yes
	97	=	Don't Know
	98	=	Refuse to Answer
	99	=	Not Applicable

APGAWHD	APGAWH. Where did someone do anything else that generates smoke, dust or particles?: BED1 = 1st Bedroom	1
	0	=	No
	1	=	Yes
	97	=	Don't Know
	98	=	Refuse to Answer
	99	=	Not Applicable

APGAWHE	APGAWH. Where did someone do anything else that generates smoke, dust or particles?: BED2 = 2nd Bedroom	1
	0	=	No
	1	=	Yes
	97	=	Don't Know
	98	=	Refuse to Answer
	99	=	Not Applicable

APGAWHF	APGAWH. Where did someone do anything else that generates smoke, dust or particles?: BED3 = 3rd Bedroom	1
	0	=	No
	1	=	Yes
	97	=	Don't Know
	98	=	Refuse to Answer
	99	=	Not Applicable

APGAWHG	APGAWH. Where did someone do anything else that generates smoke, dust or particles?: BED4 = 4th Bedroom	1
	0	=	No
	1	=	Yes
	97	=	Don't Know
	98	=	Refuse to Answer
	99	=	Not Applicable

APGAWHH	APGAWH. Where did someone do anything else that generates smoke, dust or particles?: BATH1 = 1st Bathroom	1
	0	=	No
	1	=	Yes
	97	=	Don't Know
	98	=	Refuse to Answer
	99	=	Not Applicable

APGAWHI	APGAWH. Where did someone do anything else that generates smoke, dust or particles?: BATH2 = 2nd Bathroom	1
	0	=	No
	1	=	Yes
	97	=	Don't Know
	98	=	Refuse to Answer
	99	=	Not Applicable

APGAWHJ	APGAWH. Where did someone do anything else that generates smoke, dust or particles?: BATH3 = 3rd Bathroom	1
	0	=	No
	1	=	Yes
	97	=	Don't Know
	98	=	Refuse to Answer
	99	=	Not Applicable

APGAWHK	APGAWH. Where did someone do anything else that generates smoke, dust or particles?: BAL1 = Balcony1	1
	0	=	No
	1	=	Yes
	97	=	Don't Know
	98	=	Refuse to Answer
	99	=	Not Applicable

APGAWHL	APGAWH. Where did someone do anything else that generates smoke, dust or particles?: BAL2 = Balcony2	1
	0	=	No
	1	=	Yes
	97	=	Don't Know
	98	=	Refuse to Answer
	99	=	Not Applicable

APGAWHM	APGAWH. Where did someone do anything else that generates smoke, dust or particles?: PAT = Patio	1
	0	=	No
	1	=	Yes
	97	=	Don't Know
	98	=	Refuse to Answer
	99	=	Not Applicable

APGAWHN	APGAWH. Where did someone do anything else that generates smoke, dust or particles?: LOB = Lobby	1
	0	=	No
	1	=	Yes
	97	=	Don't Know
	98	=	Refuse to Answer
	99	=	Not Applicable

APGAWHO	APGAWH. Where did someone do anything else that generates smoke, dust or particles?: HALL1 = Hallway 1	1
	0	=	No
	1	=	Yes
	97	=	Don't Know
	98	=	Refuse to Answer
	99	=	Not Applicable

APGAWHP	APGAWH. Where did someone do anything else that generates smoke, dust or particles?: HALL2 = Hallway 2	1
	0	=	No
	1	=	Yes
	97	=	Don't Know
	98	=	Refuse to Answer
	99	=	Not Applicable

APGAWHQ	APGAWH. Where did someone do anything else that generates smoke, dust or particles?: HALL3 = Hallway 3	1
	0	=	No
	1	=	Yes
	97	=	Don't Know
	98	=	Refuse to Answer
	99	=	Not Applicable

APGAWHR	APGAWH. Where did someone do anything else that generates smoke, dust or particles?: LAUN = Laundry/Utility	1
	0	=	No
	1	=	Yes
	97	=	Don't Know
	98	=	Refuse to Answer
	99	=	Not Applicable

APGAWHS	APGAWH. Where did someone do anything else that generates smoke, dust or particles?: DEN = Rec/Family Rm	1
	0	=	No
	1	=	Yes
	97	=	Don't Know
	98	=	Refuse to Answer
	99	=	Not Applicable

APGAWHT	APGAWH. Where did someone do anything else that generates smoke, dust or particles?: OTH1 = Other Room1	1
	0	=	No
	1	=	Yes
	97	=	Don't Know
	98	=	Refuse to Answer
	99	=	Not Applicable

APGAWHU	APGAWH. Where did someone do anything else that generates smoke, dust or particles?: OTH2 = Other Room2	1
	0	=	No
	1	=	Yes
	97	=	Don't Know
	98	=	Refuse to Answer
	99	=	Not Applicable

APGAWHV	APGAWH. Where did someone do anything else that generates smoke, dust or particles?: OTH3 = Other Room3	1
	0	=	No
	1	=	Yes
	97	=	Don't Know
	98	=	Refuse to Answer
	99	=	Not Applicable


PGA46.	APGAM. On a typical day in the past 7 days, how many times did someone do anything else that generates smoke, dust or particles in your home during the Morning (6 AM to Noon)?
APGAM	APGAM. On a typical day in the past 7 days, how many times did someone do anything else that generates smoke, dust or particles in your home during the Morning (6 AM to Noon)?	5
	0 - 96	=	range
	97	=	Don't Know
	98	=	Refuse to Answer
	99	=	Not Applicable

PGA47.	APGAA. On a typical day in the past 7 days, how many times did someone do anything else that generates smoke, dust or particles  in your home during the Afternoon (Noon to 6PM)?
APGAA	APGAA. On a typical day in the past 7 days, how many times did someone do anything else that generates smoke, dust or particles  in your home during the Afternoon (Noon to 6PM)?	5
	0 - 96	=	range
	97	=	Don't Know
	98	=	Refuse to Answer
	99	=	Not Applicable

PGA48.	APGAE. On a typical day in the past 7 days, how many times did someone do anything else that generates smoke, dust or particles  in your home during the Evening (6PM to Midnight)?
APGAE	APGAE. On a typical day in the past 7 days, how many times did someone do anything else that generates smoke, dust or particles  in your home during the Evening (6PM to Midnight)?	5
	0 - 96	=	range
	97	=	Don't Know
	98	=	Refuse to Answer
	99	=	Not Applicable

PGA49.	APGAN. On a typical day in the past 7 days, how many times did someone do anything else that generates smoke, dust or particles in your home during the Night (Midnight to 6AM)?
APGAN	APGAN. On a typical day in the past 7 days, how many times did someone do anything else that generates smoke, dust or particles in your home during the Night (Midnight to 6AM)?	5
	0 - 96	=	range
	97	=	Don't Know
	98	=	Refuse to Answer
	99	=	Not Applicable

PGA50.	BPGA7. In the past 7 days, how many days did you or someone in your home do anything else that generates smoke, dust or particles?
INTERVIEWER: Click "Not Applicable" if there is no such activity in the home, to skip the next 5 questions.
Examples to prompt the respondent:
·renovating (drywall, demolition, painting) producing dust, fumes, etc.
·started fire in fireplace or wood stove
·noticed pollen/spores coming inside
·sanding or wood work producing dust
·anything producing vapor, steam, droplets
·welding or work producing smoke, dust
BPGA7	BPGA7. In the past 7 days, how many days did you or someone in your home do anything else that generates smoke, dust or particles?	5
	0 - 7	=	range
	97	=	Don't Know
	98	=	Refuse to Answer
	99	=	Not Applicable

PGA51.	BPGA7SP. Specify Other particle generating item/activity
BPGA7SP	BPGA7SP. Specify Other particle generating item/activity	20

PGA52.	BPGAWH. Where did someone do anything else that generates smoke, dust or particles?
(Check all that apply)
INTERVIEWER: Use Floor Plan Sketch to prompt respondent.
BPGAWHA	BPGAWH. Where did someone do anything else that generates smoke, dust or particles?: LIV = Living Room	1
	0	=	No
	1	=	Yes
	97	=	Don't Know
	98	=	Refuse to Answer
	99	=	Not Applicable

BPGAWHB	BPGAWH. Where did someone do anything else that generates smoke, dust or particles?: KIT = Kitchen	1
	0	=	No
	1	=	Yes
	97	=	Don't Know
	98	=	Refuse to Answer
	99	=	Not Applicable

BPGAWHC	BPGAWH. Where did someone do anything else that generates smoke, dust or particles?: DIN = Dining Room	1
	0	=	No
	1	=	Yes
	97	=	Don't Know
	98	=	Refuse to Answer
	99	=	Not Applicable

BPGAWHD	BPGAWH. Where did someone do anything else that generates smoke, dust or particles?: BED1 = 1st Bedroom	1
	0	=	No
	1	=	Yes
	97	=	Don't Know
	98	=	Refuse to Answer
	99	=	Not Applicable

BPGAWHE	BPGAWH. Where did someone do anything else that generates smoke, dust or particles?: BED2 = 2nd Bedroom	1
	0	=	No
	1	=	Yes
	97	=	Don't Know
	98	=	Refuse to Answer
	99	=	Not Applicable

BPGAWHF	BPGAWH. Where did someone do anything else that generates smoke, dust or particles?: BED3 = 3rd Bedroom	1
	0	=	No
	1	=	Yes
	97	=	Don't Know
	98	=	Refuse to Answer
	99	=	Not Applicable

BPGAWHG	BPGAWH. Where did someone do anything else that generates smoke, dust or particles?: BED4 = 4th Bedroom	1
	0	=	No
	1	=	Yes
	97	=	Don't Know
	98	=	Refuse to Answer
	99	=	Not Applicable

BPGAWHH	BPGAWH. Where did someone do anything else that generates smoke, dust or particles?: BATH1 = 1st Bathroom	1
	0	=	No
	1	=	Yes
	97	=	Don't Know
	98	=	Refuse to Answer
	99	=	Not Applicable

BPGAWHI	BPGAWH. Where did someone do anything else that generates smoke, dust or particles?: BATH2 = 2nd Bathroom	1
	0	=	No
	1	=	Yes
	97	=	Don't Know
	98	=	Refuse to Answer
	99	=	Not Applicable

BPGAWHJ	BPGAWH. Where did someone do anything else that generates smoke, dust or particles?: BATH3 = 3rd Bathroom	1
	0	=	No
	1	=	Yes
	97	=	Don't Know
	98	=	Refuse to Answer
	99	=	Not Applicable

BPGAWHK	BPGAWH. Where did someone do anything else that generates smoke, dust or particles?: BAL1 = Balcony1	1
	0	=	No
	1	=	Yes
	97	=	Don't Know
	98	=	Refuse to Answer
	99	=	Not Applicable

BPGAWHL	BPGAWH. Where did someone do anything else that generates smoke, dust or particles?: BAL2 = Balcony2	1
	0	=	No
	1	=	Yes
	97	=	Don't Know
	98	=	Refuse to Answer
	99	=	Not Applicable

BPGAWHM	BPGAWH. Where did someone do anything else that generates smoke, dust or particles?: PAT = Patio	1
	0	=	No
	1	=	Yes
	97	=	Don't Know
	98	=	Refuse to Answer
	99	=	Not Applicable

BPGAWHN	BPGAWH. Where did someone do anything else that generates smoke, dust or particles?: LOB = Lobby	1
	0	=	No
	1	=	Yes
	97	=	Don't Know
	98	=	Refuse to Answer
	99	=	Not Applicable

BPGAWHO	BPGAWH. Where did someone do anything else that generates smoke, dust or particles?: HALL1 = Hallway 1	1
	0	=	No
	1	=	Yes
	97	=	Don't Know
	98	=	Refuse to Answer
	99	=	Not Applicable

BPGAWHP	BPGAWH. Where did someone do anything else that generates smoke, dust or particles?: HALL2 = Hallway 2	1
	0	=	No
	1	=	Yes
	97	=	Don't Know
	98	=	Refuse to Answer
	99	=	Not Applicable

BPGAWHQ	BPGAWH. Where did someone do anything else that generates smoke, dust or particles?: HALL3 = Hallway 3	1
	0	=	No
	1	=	Yes
	97	=	Don't Know
	98	=	Refuse to Answer
	99	=	Not Applicable

BPGAWHR	BPGAWH. Where did someone do anything else that generates smoke, dust or particles?: LAUN = Laundry/Utility	1
	0	=	No
	1	=	Yes
	97	=	Don't Know
	98	=	Refuse to Answer
	99	=	Not Applicable

BPGAWHS	BPGAWH. Where did someone do anything else that generates smoke, dust or particles?: DEN = Rec/Family Rm	1
	0	=	No
	1	=	Yes
	97	=	Don't Know
	98	=	Refuse to Answer
	99	=	Not Applicable

BPGAWHT	BPGAWH. Where did someone do anything else that generates smoke, dust or particles?: OTH1 = Other Room1	1
	0	=	No
	1	=	Yes
	97	=	Don't Know
	98	=	Refuse to Answer
	99	=	Not Applicable

BPGAWHU	BPGAWH. Where did someone do anything else that generates smoke, dust or particles?: OTH2 = Other Room2	1
	0	=	No
	1	=	Yes
	97	=	Don't Know
	98	=	Refuse to Answer
	99	=	Not Applicable

BPGAWHV	BPGAWH. Where did someone do anything else that generates smoke, dust or particles?: OTH3 = Other Room3	1
	0	=	No
	1	=	Yes
	97	=	Don't Know
	98	=	Refuse to Answer
	99	=	Not Applicable


PGA53.	BPGAM. On a typical day in the past 7 days, how many times did someone do anything else that generates smoke, dust or particles in your home during the Morning (6 AM to Noon)?
BPGAM	BPGAM. On a typical day in the past 7 days, how many times did someone do anything else that generates smoke, dust or particles in your home during the Morning (6 AM to Noon)?	5
	0 - 96	=	range
	97	=	Don't Know
	98	=	Refuse to Answer
	99	=	Not Applicable

PGA54.	BPGAA. On a typical day in the past 7 days, how many times did someone do anything else that generates smoke, dust or particles in your home during the Afternoon (Noon to 6PM)?
BPGAA	BPGAA. On a typical day in the past 7 days, how many times did someone do anything else that generates smoke, dust or particles in your home during the Afternoon (Noon to 6PM)?	5
	0 - 96	=	range
	97	=	Don't Know
	98	=	Refuse to Answer
	99	=	Not Applicable

PGA55.	BPGAE. On a typical day in the past 7 days, how many times did someone do anything else that generates smoke, dust or particles in your home during the Evening (6PM to Midnight)?
BPGAE	BPGAE. On a typical day in the past 7 days, how many times did someone do anything else that generates smoke, dust or particles in your home during the Evening (6PM to Midnight)?	5
	0 - 96	=	range
	97	=	Don't Know
	98	=	Refuse to Answer
	99	=	Not Applicable

PGA56.	BPGAN. On a typical day in the past 7 days, how many times did someone do anything else that generates smoke, dust or particles in your home during the Night (Midnight to 6AM)?
BPGAN	BPGAN. On a typical day in the past 7 days, how many times did someone do anything else that generates smoke, dust or particles in your home during the Night (Midnight to 6AM)?	5
	0 - 96	=	range
	97	=	Don't Know
	98	=	Refuse to Answer
	99	=	Not Applicable

PGA57.	JUMPBK6
Interviewer: Do you need to jump to a previous section?
If so, click YES to return to the start of the interview, where you can then choose to jump ahead to any section.
Click NO to continue.
JUMPBK6	JUMPBK6. Jump back to a previous section?	1
	0	=	No
	1	=	Yes
	7	=	Don't Know
	8	=	Refuse to Answer
	9	=	Not Applicable

RTU1.	TYPEC1. Which types of tobacco has [Response to RES1] ever smoked, even a puff? 
(mark all that apply)
TYPEC1A	TYPEC1. Which types of tobacco has [TC] ever smoked, even a puff? (mark all that apply): cigarette	1
	0	=	No
	1	=	Yes
	7	=	Don't Know
	8	=	Refuse to Answer
	9	=	Not Applicable

TYPEC1B	TYPEC1. Which types of tobacco has [TC] ever smoked, even a puff? (mark all that apply): cigar	1
	0	=	No
	1	=	Yes
	7	=	Don't Know
	8	=	Refuse to Answer
	9	=	Not Applicable

TYPEC1C	TYPEC1. Which types of tobacco has [TC] ever smoked, even a puff? (mark all that apply): pipe	1
	0	=	No
	1	=	Yes
	7	=	Don't Know
	8	=	Refuse to Answer
	9	=	Not Applicable

TYPEC1D	TYPEC1. Which types of tobacco has [TC] ever smoked, even a puff? (mark all that apply): waterpipe/hookah	1
	0	=	No
	1	=	Yes
	7	=	Don't Know
	8	=	Refuse to Answer
	9	=	Not Applicable

TYPEC1E	TYPEC1. Which types of tobacco has [TC] ever smoked, even a puff? (mark all that apply): other (specify)	1
	0	=	No
	1	=	Yes
	7	=	Don't Know
	8	=	Refuse to Answer
	9	=	Not Applicable

TYPEC1F	TYPEC1. Which types of tobacco has [TC] ever smoked, even a puff? (mark all that apply): none	1
	0	=	No
	1	=	Yes
	7	=	Don't Know
	8	=	Refuse to Answer
	9	=	Not Applicable


RTU2.	TYPEC1S. Please specify other type of tobacco smoked ever
TYPEC1S	TYPEC1S. Please specify other type of tobacco smoked ever	20

RTU3.	TYP30C1. During the past 30 days which of the following types of tobacco has [Response to RES1] smoked? 
(mark all that apply)
TYP30C1A	TYP30C1. During the past 30 days which of the following types of tobacco has [TC] smoked?: cigarette	1
	0	=	No
	1	=	Yes
	7	=	Don't Know
	8	=	Refuse to Answer
	9	=	Not Applicable

TYP30C1B	TYP30C1. During the past 30 days which of the following types of tobacco has [TC] smoked?: cigar	1
	0	=	No
	1	=	Yes
	7	=	Don't Know
	8	=	Refuse to Answer
	9	=	Not Applicable

TYP30C1C	TYP30C1. During the past 30 days which of the following types of tobacco has [TC] smoked?: pipe	1
	0	=	No
	1	=	Yes
	7	=	Don't Know
	8	=	Refuse to Answer
	9	=	Not Applicable

TYP30C1D	TYP30C1. During the past 30 days which of the following types of tobacco has [TC] smoked?: waterpipe/hookah	1
	0	=	No
	1	=	Yes
	7	=	Don't Know
	8	=	Refuse to Answer
	9	=	Not Applicable

TYP30C1E	TYP30C1. During the past 30 days which of the following types of tobacco has [TC] smoked?: other (specify)	1
	0	=	No
	1	=	Yes
	7	=	Don't Know
	8	=	Refuse to Answer
	9	=	Not Applicable

TYP30C1F	TYP30C1. During the past 30 days which of the following types of tobacco has [TC] smoked?: none	1
	0	=	No
	1	=	Yes
	7	=	Don't Know
	8	=	Refuse to Answer
	9	=	Not Applicable


RTU4.	TYP30C1S. Please specify other type of tobacco smoked during the past 30 days
TYP30C1S	TYP30C1S. Please specify other type of tobacco smoked during the past 30 days	20

RTU5.	LIFEC1. Has [Response to RES1] smoked more than 100 cigarettes in their lifetime?
LIFEC1	LIFEC1. Has [TC] smoked more than 100 cigarettes in their lifetime?	1
	0	=	No
	1	=	Yes
	7	=	Don't Know
	8	=	Refuse to Answer
	9	=	Not Applicable

RTU6.	SMKNOWC1. Does [Response to RES1] currently smoke cigarettes not at all, some days, or every day?
SMKNOWC1	SMKNOWC1. Does [TC] currently smoke cigarettes not at all, some days, or every day?	1
	0	=	Not at All
	1	=	Some Days
	2	=	Every Day
	7	=	Don't Know
	8	=	Refuse to Answer
	9	=	Not Applicable

RTU7.	TYPEC2. Which types of tobacco has [Response to RES4] ever smoked, even a puff? 
(mark all that apply)
TYPEC2A	TYPEC2. Which types of tobacco has [FNCHLD2] ever smoked, even a puff? (mark all that apply): cigarette	1
	0	=	No
	1	=	Yes
	7	=	Don't Know
	8	=	Refuse to Answer
	9	=	Not Applicable

TYPEC2B	TYPEC2. Which types of tobacco has [FNCHLD2] ever smoked, even a puff? (mark all that apply): cigar	1
	0	=	No
	1	=	Yes
	7	=	Don't Know
	8	=	Refuse to Answer
	9	=	Not Applicable

TYPEC2C	TYPEC2. Which types of tobacco has [FNCHLD2] ever smoked, even a puff? (mark all that apply): pipe	1
	0	=	No
	1	=	Yes
	7	=	Don't Know
	8	=	Refuse to Answer
	9	=	Not Applicable

TYPEC2D	TYPEC2. Which types of tobacco has [FNCHLD2] ever smoked, even a puff? (mark all that apply): waterpipe/hookah	1
	0	=	No
	1	=	Yes
	7	=	Don't Know
	8	=	Refuse to Answer
	9	=	Not Applicable

TYPEC2E	TYPEC2. Which types of tobacco has [FNCHLD2] ever smoked, even a puff? (mark all that apply): other (specify)	1
	0	=	No
	1	=	Yes
	7	=	Don't Know
	8	=	Refuse to Answer
	9	=	Not Applicable

TYPEC2F	TYPEC2. Which types of tobacco has [FNCHLD2] ever smoked, even a puff? (mark all that apply): none	1
	0	=	No
	1	=	Yes
	7	=	Don't Know
	8	=	Refuse to Answer
	9	=	Not Applicable


RTU8.	TYPEC2S. Please specify other type of tobacco smoked ever
TYPEC2S	TYPEC2S. Please specify other type of tobacco smoked ever	20

RTU9.	TYP30C2. During the past 30 days which of the following types of tobacco has [Response to RES4] smoked? 
(mark all that apply)
TYP30C2A	TYP30C2. During the past 30 days which of the following types of tobacco has [FNCHLD2] smoked?: cigarette	1
	0	=	No
	1	=	Yes
	7	=	Don't Know
	8	=	Refuse to Answer
	9	=	Not Applicable

TYP30C2B	TYP30C2. During the past 30 days which of the following types of tobacco has [FNCHLD2] smoked?: cigar	1
	0	=	No
	1	=	Yes
	7	=	Don't Know
	8	=	Refuse to Answer
	9	=	Not Applicable

TYP30C2C	TYP30C2. During the past 30 days which of the following types of tobacco has [FNCHLD2] smoked?: pipe	1
	0	=	No
	1	=	Yes
	7	=	Don't Know
	8	=	Refuse to Answer
	9	=	Not Applicable

TYP30C2D	TYP30C2. During the past 30 days which of the following types of tobacco has [FNCHLD2] smoked?: waterpipe/hookah	1
	0	=	No
	1	=	Yes
	7	=	Don't Know
	8	=	Refuse to Answer
	9	=	Not Applicable

TYP30C2E	TYP30C2. During the past 30 days which of the following types of tobacco has [FNCHLD2] smoked?: other (specify)	1
	0	=	No
	1	=	Yes
	7	=	Don't Know
	8	=	Refuse to Answer
	9	=	Not Applicable

TYP30C2F	TYP30C2. During the past 30 days which of the following types of tobacco has [FNCHLD2] smoked?: none	1
	0	=	No
	1	=	Yes
	7	=	Don't Know
	8	=	Refuse to Answer
	9	=	Not Applicable


RTU10.	TYP30C2S. Please specify other type of tobacco smoked during the past 30 days
TYP30C2S	TYP30C2S. Please specify other type of tobacco smoked during the past 30 days	20

RTU11.	LIFEC2. Has [Response to RES4] smoked more than 100 cigarettes in their lifetime?
LIFEC2	LIFEC2. Has [FNCHLD2] smoked more than 100 cigarettes in their lifetime?	1
	0	=	No
	1	=	Yes
	7	=	Don't Know
	8	=	Refuse to Answer
	9	=	Not Applicable

RTU12.	SMKNOWC2. Does [Response to RES4] currently smoke cigarettes not at all, some days, or every day?
SMKNOWC2	SMKNOWC2. Does [FNCHLD2] currently smoke cigarettes not at all, some days, or every day?	1
	0	=	Not at All
	1	=	Some Days
	2	=	Every Day
	7	=	Don't Know
	8	=	Refuse to Answer
	9	=	Not Applicable

RTU13.	TYPEC3. Which types of tobacco has [Response to RES9] ever smoked, even a puff? 
(mark all that apply)
TYPEC3A	TYPEC3. Which types of tobacco has [FNCHLD3] ever smoked, even a puff? (mark all that apply): cigarette	1
	0	=	No
	1	=	Yes
	7	=	Don't Know
	8	=	Refuse to Answer
	9	=	Not Applicable

TYPEC3B	TYPEC3. Which types of tobacco has [FNCHLD3] ever smoked, even a puff? (mark all that apply): cigar	1
	0	=	No
	1	=	Yes
	7	=	Don't Know
	8	=	Refuse to Answer
	9	=	Not Applicable

TYPEC3C	TYPEC3. Which types of tobacco has [FNCHLD3] ever smoked, even a puff? (mark all that apply): pipe	1
	0	=	No
	1	=	Yes
	7	=	Don't Know
	8	=	Refuse to Answer
	9	=	Not Applicable

TYPEC3D	TYPEC3. Which types of tobacco has [FNCHLD3] ever smoked, even a puff? (mark all that apply): waterpipe/hookah	1
	0	=	No
	1	=	Yes
	7	=	Don't Know
	8	=	Refuse to Answer
	9	=	Not Applicable

TYPEC3E	TYPEC3. Which types of tobacco has [FNCHLD3] ever smoked, even a puff? (mark all that apply): other (specify)	1
	0	=	No
	1	=	Yes
	7	=	Don't Know
	8	=	Refuse to Answer
	9	=	Not Applicable

TYPEC3F	TYPEC3. Which types of tobacco has [FNCHLD3] ever smoked, even a puff? (mark all that apply): none	1
	0	=	No
	1	=	Yes
	7	=	Don't Know
	8	=	Refuse to Answer
	9	=	Not Applicable


RTU14.	TYPEC3S. Please specify other type of tobacco smoked ever
TYPEC3S	TYPEC3S. Please specify other type of tobacco smoked ever	20

RTU15.	TYP30C3. During the past 30 days which of the following types of tobacco has [Response to RES9] smoked? 
(mark all that apply)
TYP30C3A	TYP30C3. During the past 30 days which of the following types of tobacco has [FNCHLD3] smoked?: cigarette	1
	0	=	No
	1	=	Yes
	7	=	Don't Know
	8	=	Refuse to Answer
	9	=	Not Applicable

TYP30C3B	TYP30C3. During the past 30 days which of the following types of tobacco has [FNCHLD3] smoked?: cigar	1
	0	=	No
	1	=	Yes
	7	=	Don't Know
	8	=	Refuse to Answer
	9	=	Not Applicable

TYP30C3C	TYP30C3. During the past 30 days which of the following types of tobacco has [FNCHLD3] smoked?: pipe	1
	0	=	No
	1	=	Yes
	7	=	Don't Know
	8	=	Refuse to Answer
	9	=	Not Applicable

TYP30C3D	TYP30C3. During the past 30 days which of the following types of tobacco has [FNCHLD3] smoked?: waterpipe/hookah	1
	0	=	No
	1	=	Yes
	7	=	Don't Know
	8	=	Refuse to Answer
	9	=	Not Applicable

TYP30C3E	TYP30C3. During the past 30 days which of the following types of tobacco has [FNCHLD3] smoked?: other (specify)	1
	0	=	No
	1	=	Yes
	7	=	Don't Know
	8	=	Refuse to Answer
	9	=	Not Applicable

TYP30C3F	TYP30C3. During the past 30 days which of the following types of tobacco has [FNCHLD3] smoked?: none	1
	0	=	No
	1	=	Yes
	7	=	Don't Know
	8	=	Refuse to Answer
	9	=	Not Applicable


RTU16.	TYP30C3S. Please specify other type of tobacco smoked during the past 30 days
TYP30C3S	TYP30C3S. Please specify other type of tobacco smoked during the past 30 days	20

RTU17.	LIFEC3. Has [Response to RES9] smoked more than 100 cigarettes in their lifetime?
LIFEC3	LIFEC3. Has [FNCHLD3] smoked more than 100 cigarettes in their lifetime?	1
	0	=	No
	1	=	Yes
	7	=	Don't Know
	8	=	Refuse to Answer
	9	=	Not Applicable

RTU18.	SMKNOWC3. Does [Response to RES9] currently smoke cigarettes not at all, some days, or every day?
SMKNOWC3	SMKNOWC3. Does [FNCHLD3] currently smoke cigarettes not at all, some days, or every day?	1
	0	=	Not at All
	1	=	Some Days
	2	=	Every Day
	7	=	Don't Know
	8	=	Refuse to Answer
	9	=	Not Applicable

RTU19.	TYPEC4. Which types of tobacco has [Response to RES14] ever smoked, even a puff? 
(mark all that apply)
TYPEC4A	TYPEC4. Which types of tobacco has [FNCHLD4] ever smoked, even a puff? (mark all that apply): cigarette	1
	0	=	No
	1	=	Yes
	7	=	Don't Know
	8	=	Refuse to Answer
	9	=	Not Applicable

TYPEC4B	TYPEC4. Which types of tobacco has [FNCHLD4] ever smoked, even a puff? (mark all that apply): cigar	1
	0	=	No
	1	=	Yes
	7	=	Don't Know
	8	=	Refuse to Answer
	9	=	Not Applicable

TYPEC4C	TYPEC4. Which types of tobacco has [FNCHLD4] ever smoked, even a puff? (mark all that apply): pipe	1
	0	=	No
	1	=	Yes
	7	=	Don't Know
	8	=	Refuse to Answer
	9	=	Not Applicable

TYPEC4D	TYPEC4. Which types of tobacco has [FNCHLD4] ever smoked, even a puff? (mark all that apply): waterpipe/hookah	1
	0	=	No
	1	=	Yes
	7	=	Don't Know
	8	=	Refuse to Answer
	9	=	Not Applicable

TYPEC4E	TYPEC4. Which types of tobacco has [FNCHLD4] ever smoked, even a puff? (mark all that apply): other (specify)	1
	0	=	No
	1	=	Yes
	7	=	Don't Know
	8	=	Refuse to Answer
	9	=	Not Applicable

TYPEC4F	TYPEC4. Which types of tobacco has [FNCHLD4] ever smoked, even a puff? (mark all that apply): none	1
	0	=	No
	1	=	Yes
	7	=	Don't Know
	8	=	Refuse to Answer
	9	=	Not Applicable


RTU20.	TYPEC4S. Please specify other type of tobacco smoked ever
TYPEC4S	TYPEC4S. Please specify other type of tobacco smoked ever	20

RTU21.	TYP30C4. During the past 30 days which of the following types of tobacco has [Response to RES14] smoked? 
(mark all that apply)
TYP30C4A	TYP30C4. During the past 30 days which of the following types of tobacco has [FNCHLD4] smoked?: cigarette	1
	0	=	No
	1	=	Yes
	7	=	Don't Know
	8	=	Refuse to Answer
	9	=	Not Applicable

TYP30C4B	TYP30C4. During the past 30 days which of the following types of tobacco has [FNCHLD4] smoked?: cigar	1
	0	=	No
	1	=	Yes
	7	=	Don't Know
	8	=	Refuse to Answer
	9	=	Not Applicable

TYP30C4C	TYP30C4. During the past 30 days which of the following types of tobacco has [FNCHLD4] smoked?: pipe	1
	0	=	No
	1	=	Yes
	7	=	Don't Know
	8	=	Refuse to Answer
	9	=	Not Applicable

TYP30C4D	TYP30C4. During the past 30 days which of the following types of tobacco has [FNCHLD4] smoked?: waterpipe/hookah	1
	0	=	No
	1	=	Yes
	7	=	Don't Know
	8	=	Refuse to Answer
	9	=	Not Applicable

TYP30C4E	TYP30C4. During the past 30 days which of the following types of tobacco has [FNCHLD4] smoked?: other (specify)	1
	0	=	No
	1	=	Yes
	7	=	Don't Know
	8	=	Refuse to Answer
	9	=	Not Applicable

TYP30C4F	TYP30C4. During the past 30 days which of the following types of tobacco has [FNCHLD4] smoked?: none	1
	0	=	No
	1	=	Yes
	7	=	Don't Know
	8	=	Refuse to Answer
	9	=	Not Applicable


RTU22.	TYP30C4S. Please specify other type of tobacco smoked during the past 30 days
TYP30C4S	TYP30C4S. Please specify other type of tobacco smoked during the past 30 days	20

RTU23.	LIFEC4. Has [Response to RES14] smoked more than 100 cigarettes in their lifetime?
LIFEC4	LIFEC4. Has [FNCHLD4] smoked more than 100 cigarettes in their lifetime?	1
	0	=	No
	1	=	Yes
	7	=	Don't Know
	8	=	Refuse to Answer
	9	=	Not Applicable

RTU24.	SMKNOWC4. Does [Response to RES14] currently smoke cigarettes not at all, some days, or every day?
SMKNOWC4	SMKNOWC4. Does [FNCHLD4] currently smoke cigarettes not at all, some days, or every day?	1
	0	=	Not at All
	1	=	Some Days
	2	=	Every Day
	7	=	Don't Know
	8	=	Refuse to Answer
	9	=	Not Applicable

RTU25.	TYPEC5. Which types of tobacco has [Response to RES19] ever smoked, even a puff? 
(mark all that apply)
TYPEC5A	TYPEC5. Which types of tobacco has [FNCHLD5] ever smoked, even a puff? (mark all that apply): cigarette	1
	0	=	No
	1	=	Yes
	7	=	Don't Know
	8	=	Refuse to Answer
	9	=	Not Applicable

TYPEC5B	TYPEC5. Which types of tobacco has [FNCHLD5] ever smoked, even a puff? (mark all that apply): cigar	1
	0	=	No
	1	=	Yes
	7	=	Don't Know
	8	=	Refuse to Answer
	9	=	Not Applicable

TYPEC5C	TYPEC5. Which types of tobacco has [FNCHLD5] ever smoked, even a puff? (mark all that apply): pipe	1
	0	=	No
	1	=	Yes
	7	=	Don't Know
	8	=	Refuse to Answer
	9	=	Not Applicable

TYPEC5D	TYPEC5. Which types of tobacco has [FNCHLD5] ever smoked, even a puff? (mark all that apply): waterpipe/hookah	1
	0	=	No
	1	=	Yes
	7	=	Don't Know
	8	=	Refuse to Answer
	9	=	Not Applicable

TYPEC5E	TYPEC5. Which types of tobacco has [FNCHLD5] ever smoked, even a puff? (mark all that apply): other (specify)	1
	0	=	No
	1	=	Yes
	7	=	Don't Know
	8	=	Refuse to Answer
	9	=	Not Applicable

TYPEC5F	TYPEC5. Which types of tobacco has [FNCHLD5] ever smoked, even a puff? (mark all that apply): none	1
	0	=	No
	1	=	Yes
	7	=	Don't Know
	8	=	Refuse to Answer
	9	=	Not Applicable


RTU26.	TYPEC5S. Please specify other type of tobacco smoked ever
TYPEC5S	TYPEC5S. Please specify other type of tobacco smoked ever	20

RTU27.	TYP30C5. During the past 30 days which of the following types of tobacco has [Response to RES19] smoked? 
(mark all that apply)
TYP30C5A	TYP30C5. During the past 30 days which of the following types of tobacco has [FNCHLD5] smoked?: cigarette	1
	0	=	No
	1	=	Yes
	7	=	Don't Know
	8	=	Refuse to Answer
	9	=	Not Applicable

TYP30C5B	TYP30C5. During the past 30 days which of the following types of tobacco has [FNCHLD5] smoked?: cigar	1
	0	=	No
	1	=	Yes
	7	=	Don't Know
	8	=	Refuse to Answer
	9	=	Not Applicable

TYP30C5C	TYP30C5. During the past 30 days which of the following types of tobacco has [FNCHLD5] smoked?: pipe	1
	0	=	No
	1	=	Yes
	7	=	Don't Know
	8	=	Refuse to Answer
	9	=	Not Applicable

TYP30C5D	TYP30C5. During the past 30 days which of the following types of tobacco has [FNCHLD5] smoked?: waterpipe/hookah	1
	0	=	No
	1	=	Yes
	7	=	Don't Know
	8	=	Refuse to Answer
	9	=	Not Applicable

TYP30C5E	TYP30C5. During the past 30 days which of the following types of tobacco has [FNCHLD5] smoked?: other (specify)	1
	0	=	No
	1	=	Yes
	7	=	Don't Know
	8	=	Refuse to Answer
	9	=	Not Applicable

TYP30C5F	TYP30C5. During the past 30 days which of the following types of tobacco has [FNCHLD5] smoked?: none	1
	0	=	No
	1	=	Yes
	7	=	Don't Know
	8	=	Refuse to Answer
	9	=	Not Applicable


RTU28.	TYP30C5S. Please specify other type of tobacco smoked during the past 30 days
TYP30C5S	TYP30C5S. Please specify other type of tobacco smoked during the past 30 days	20

RTU29.	LIFEC5. Has [Response to RES19] smoked more than 100 cigarettes in their lifetime?
LIFEC5	LIFEC5. Has [FNCHLD5] smoked more than 100 cigarettes in their lifetime?	1
	0	=	No
	1	=	Yes
	7	=	Don't Know
	8	=	Refuse to Answer
	9	=	Not Applicable

RTU30.	SMKNOWC5. Does [Response to RES19] currently smoke cigarettes not at all, some days, or every day?
SMKNOWC5	SMKNOWC5. Does [FNCHLD5] currently smoke cigarettes not at all, some days, or every day?	1
	0	=	Not at All
	1	=	Some Days
	2	=	Every Day
	7	=	Don't Know
	8	=	Refuse to Answer
	9	=	Not Applicable

RTU31.	TYPEA1. Which types of tobacco has [Response to RES24] ever smoked, even a puff? 
(mark all that apply)
TYPEA1A	TYPEA1. Which types of tobacco has [TP] ever smoked, even a puff? (mark all that apply): cigarette	1
	0	=	No
	1	=	Yes
	7	=	Don't Know
	8	=	Refuse to Answer
	9	=	Not Applicable

TYPEA1B	TYPEA1. Which types of tobacco has [TP] ever smoked, even a puff? (mark all that apply): cigar	1
	0	=	No
	1	=	Yes
	7	=	Don't Know
	8	=	Refuse to Answer
	9	=	Not Applicable

TYPEA1C	TYPEA1. Which types of tobacco has [TP] ever smoked, even a puff? (mark all that apply): pipe	1
	0	=	No
	1	=	Yes
	7	=	Don't Know
	8	=	Refuse to Answer
	9	=	Not Applicable

TYPEA1D	TYPEA1. Which types of tobacco has [TP] ever smoked, even a puff? (mark all that apply): waterpipe/hookah	1
	0	=	No
	1	=	Yes
	7	=	Don't Know
	8	=	Refuse to Answer
	9	=	Not Applicable

TYPEA1E	TYPEA1. Which types of tobacco has [TP] ever smoked, even a puff? (mark all that apply): other (specify)	1
	0	=	No
	1	=	Yes
	7	=	Don't Know
	8	=	Refuse to Answer
	9	=	Not Applicable

TYPEA1F	TYPEA1. Which types of tobacco has [TP] ever smoked, even a puff? (mark all that apply): none	1
	0	=	No
	1	=	Yes
	7	=	Don't Know
	8	=	Refuse to Answer
	9	=	Not Applicable


RTU32.	TYPEA1S. Please specify other type of tobacco smoked ever
TYPEA1S	TYPEA1S. Please specify other type of tobacco smoked ever	20

RTU33.	TYP30A1. During the past 30 days which of the following types of tobacco has [Response to RES24] smoked? 
(mark all that apply)
TYP30A1A	TYP30A1. During the past 30 days which of the following types of tobacco has [TP] smoked?: cigarette	1
	0	=	No
	1	=	Yes
	7	=	Don't Know
	8	=	Refuse to Answer
	9	=	Not Applicable

TYP30A1B	TYP30A1. During the past 30 days which of the following types of tobacco has [TP] smoked?: cigar	1
	0	=	No
	1	=	Yes
	7	=	Don't Know
	8	=	Refuse to Answer
	9	=	Not Applicable

TYP30A1C	TYP30A1. During the past 30 days which of the following types of tobacco has [TP] smoked?: pipe	1
	0	=	No
	1	=	Yes
	7	=	Don't Know
	8	=	Refuse to Answer
	9	=	Not Applicable

TYP30A1D	TYP30A1. During the past 30 days which of the following types of tobacco has [TP] smoked?: waterpipe/hookah	1
	0	=	No
	1	=	Yes
	7	=	Don't Know
	8	=	Refuse to Answer
	9	=	Not Applicable

TYP30A1E	TYP30A1. During the past 30 days which of the following types of tobacco has [TP] smoked?: other (specify)	1
	0	=	No
	1	=	Yes
	7	=	Don't Know
	8	=	Refuse to Answer
	9	=	Not Applicable

TYP30A1F	TYP30A1. During the past 30 days which of the following types of tobacco has [TP] smoked?: none	1
	0	=	No
	1	=	Yes
	7	=	Don't Know
	8	=	Refuse to Answer
	9	=	Not Applicable


RTU34.	TYP30A1S. Please specify other type of tobacco smoked during the past 30 days
TYP30A1S	TYP30A1S. Please specify other type of tobacco smoked during the past 30 days	20

RTU35.	LIFEA1. Has [Response to RES24] smoked more than 100 cigarettes in their lifetime?
LIFEA1	LIFEA1. Has [TP] smoked more than 100 cigarettes in their lifetime?	1
	0	=	No
	1	=	Yes
	7	=	Don't Know
	8	=	Refuse to Answer
	9	=	Not Applicable

RTU36.	SMKNOWA1. Does [Response to RES24] currently smoke cigarettes not at all, some days, or every day?
SMKNOWA1	SMKNOWA1. Does [TP] currently smoke cigarettes not at all, some days, or every day?	1
	0	=	Not at All
	1	=	Some Days
	2	=	Every Day
	7	=	Don't Know
	8	=	Refuse to Answer
	9	=	Not Applicable

RTU37.	TYPEA2. Which types of tobacco has [Response to RES29] ever smoked, even a puff? 
(mark all that apply)
TYPEA2A	TYPEA2. Which types of tobacco has [FNADLT2] ever smoked, even a puff? (mark all that apply): cigarette	1
	0	=	No
	1	=	Yes
	7	=	Don't Know
	8	=	Refuse to Answer
	9	=	Not Applicable

TYPEA2B	TYPEA2. Which types of tobacco has [FNADLT2] ever smoked, even a puff? (mark all that apply): cigar	1
	0	=	No
	1	=	Yes
	7	=	Don't Know
	8	=	Refuse to Answer
	9	=	Not Applicable

TYPEA2C	TYPEA2. Which types of tobacco has [FNADLT2] ever smoked, even a puff? (mark all that apply): pipe	1
	0	=	No
	1	=	Yes
	7	=	Don't Know
	8	=	Refuse to Answer
	9	=	Not Applicable

TYPEA2D	TYPEA2. Which types of tobacco has [FNADLT2] ever smoked, even a puff? (mark all that apply): waterpipe/hookah	1
	0	=	No
	1	=	Yes
	7	=	Don't Know
	8	=	Refuse to Answer
	9	=	Not Applicable

TYPEA2E	TYPEA2. Which types of tobacco has [FNADLT2] ever smoked, even a puff? (mark all that apply): other (specify)	1
	0	=	No
	1	=	Yes
	7	=	Don't Know
	8	=	Refuse to Answer
	9	=	Not Applicable

TYPEA2F	TYPEA2. Which types of tobacco has [FNADLT2] ever smoked, even a puff? (mark all that apply): none	1
	0	=	No
	1	=	Yes
	7	=	Don't Know
	8	=	Refuse to Answer
	9	=	Not Applicable


RTU38.	TYPEA2S. Please specify other type of tobacco smoked ever
TYPEA2S	TYPEA2S. Please specify other type of tobacco smoked ever	20

RTU39.	TYP30A2. During the past 30 days which of the following types of tobacco has [Response to RES29] smoked? 
(mark all that apply)
TYP30A2A	TYP30A2. During the past 30 days which of the following types of tobacco has [FNADLT2] smoked?: cigarette	1
	0	=	No
	1	=	Yes
	7	=	Don't Know
	8	=	Refuse to Answer
	9	=	Not Applicable

TYP30A2B	TYP30A2. During the past 30 days which of the following types of tobacco has [FNADLT2] smoked?: cigar	1
	0	=	No
	1	=	Yes
	7	=	Don't Know
	8	=	Refuse to Answer
	9	=	Not Applicable

TYP30A2C	TYP30A2. During the past 30 days which of the following types of tobacco has [FNADLT2] smoked?: pipe	1
	0	=	No
	1	=	Yes
	7	=	Don't Know
	8	=	Refuse to Answer
	9	=	Not Applicable

TYP30A2D	TYP30A2. During the past 30 days which of the following types of tobacco has [FNADLT2] smoked?: waterpipe/hookah	1
	0	=	No
	1	=	Yes
	7	=	Don't Know
	8	=	Refuse to Answer
	9	=	Not Applicable

TYP30A2E	TYP30A2. During the past 30 days which of the following types of tobacco has [FNADLT2] smoked?: other (specify)	1
	0	=	No
	1	=	Yes
	7	=	Don't Know
	8	=	Refuse to Answer
	9	=	Not Applicable

TYP30A2F	TYP30A2. During the past 30 days which of the following types of tobacco has [FNADLT2] smoked?: none	1
	0	=	No
	1	=	Yes
	7	=	Don't Know
	8	=	Refuse to Answer
	9	=	Not Applicable


RTU40.	TYP30A2S. Please specify other type of tobacco smoked during the past 30 days
TYP30A2S	TYP30A2S. Please specify other type of tobacco smoked during the past 30 days	20

RTU41.	LIFEA2. Has [Response to RES29] smoked more than 100 cigarettes in their lifetime?
LIFEA2	LIFEA2. Has [FNADLT2] smoked more than 100 cigarettes in their lifetime?	1
	0	=	No
	1	=	Yes
	7	=	Don't Know
	8	=	Refuse to Answer
	9	=	Not Applicable

RTU42.	SMKNOWA2. Does [Response to RES29] currently smoke cigarettes not at all, some days, or every day?
SMKNOWA2	SMKNOWA2. Does [FNADLT2] currently smoke cigarettes not at all, some days, or every day?	1
	0	=	Not at All
	1	=	Some Days
	2	=	Every Day
	7	=	Don't Know
	8	=	Refuse to Answer
	9	=	Not Applicable

RTU43.	TYPEA3. Which types of tobacco has [Response to RES32] ever smoked, even a puff? 
(mark all that apply)
TYPEA3A	TYPEA3. Which types of tobacco has [FNADLT3] ever smoked, even a puff? (mark all that apply): cigarette	1
	0	=	No
	1	=	Yes
	7	=	Don't Know
	8	=	Refuse to Answer
	9	=	Not Applicable

TYPEA3B	TYPEA3. Which types of tobacco has [FNADLT3] ever smoked, even a puff? (mark all that apply): cigar	1
	0	=	No
	1	=	Yes
	7	=	Don't Know
	8	=	Refuse to Answer
	9	=	Not Applicable

TYPEA3C	TYPEA3. Which types of tobacco has [FNADLT3] ever smoked, even a puff? (mark all that apply): pipe	1
	0	=	No
	1	=	Yes
	7	=	Don't Know
	8	=	Refuse to Answer
	9	=	Not Applicable

TYPEA3D	TYPEA3. Which types of tobacco has [FNADLT3] ever smoked, even a puff? (mark all that apply): waterpipe/hookah	1
	0	=	No
	1	=	Yes
	7	=	Don't Know
	8	=	Refuse to Answer
	9	=	Not Applicable

TYPEA3E	TYPEA3. Which types of tobacco has [FNADLT3] ever smoked, even a puff? (mark all that apply): other (specify)	1
	0	=	No
	1	=	Yes
	7	=	Don't Know
	8	=	Refuse to Answer
	9	=	Not Applicable

TYPEA3F	TYPEA3. Which types of tobacco has [FNADLT3] ever smoked, even a puff? (mark all that apply): none	1
	0	=	No
	1	=	Yes
	7	=	Don't Know
	8	=	Refuse to Answer
	9	=	Not Applicable


RTU44.	TYPEA3S. Please specify other type of tobacco smoked ever
TYPEA3S	TYPEA3S. Please specify other type of tobacco smoked ever	20

RTU45.	TYP30A3. During the past 30 days which of the following types of tobacco has [Response to RES32] smoked? 
(mark all that apply)
TYP30A3A	TYP30A3. During the past 30 days which of the following types of tobacco has [FNADLT3] smoked?: cigarette	1
	0	=	No
	1	=	Yes
	7	=	Don't Know
	8	=	Refuse to Answer
	9	=	Not Applicable

TYP30A3B	TYP30A3. During the past 30 days which of the following types of tobacco has [FNADLT3] smoked?: cigar	1
	0	=	No
	1	=	Yes
	7	=	Don't Know
	8	=	Refuse to Answer
	9	=	Not Applicable

TYP30A3C	TYP30A3. During the past 30 days which of the following types of tobacco has [FNADLT3] smoked?: pipe	1
	0	=	No
	1	=	Yes
	7	=	Don't Know
	8	=	Refuse to Answer
	9	=	Not Applicable

TYP30A3D	TYP30A3. During the past 30 days which of the following types of tobacco has [FNADLT3] smoked?: waterpipe/hookah	1
	0	=	No
	1	=	Yes
	7	=	Don't Know
	8	=	Refuse to Answer
	9	=	Not Applicable

TYP30A3E	TYP30A3. During the past 30 days which of the following types of tobacco has [FNADLT3] smoked?: other (specify)	1
	0	=	No
	1	=	Yes
	7	=	Don't Know
	8	=	Refuse to Answer
	9	=	Not Applicable

TYP30A3F	TYP30A3. During the past 30 days which of the following types of tobacco has [FNADLT3] smoked?: none	1
	0	=	No
	1	=	Yes
	7	=	Don't Know
	8	=	Refuse to Answer
	9	=	Not Applicable


RTU46.	TYP30A3S. Please specify other type of tobacco smoked during the past 30 days
TYP30A3S	TYP30A3S. Please specify other type of tobacco smoked during the past 30 days	20

RTU47.	LIFEA3. Has [Response to RES32] smoked more than 100 cigarettes in their lifetime?
LIFEA3	LIFEA3. Has [FNADLT3] smoked more than 100 cigarettes in their lifetime?	1
	0	=	No
	1	=	Yes
	7	=	Don't Know
	8	=	Refuse to Answer
	9	=	Not Applicable

RTU48.	SMKNOWA3. Does [Response to RES32] currently smoke cigarettes not at all, some days, or every day?
SMKNOWA3	SMKNOWA3. Does [FNADLT3] currently smoke cigarettes not at all, some days, or every day?	1
	0	=	Not at All
	1	=	Some Days
	2	=	Every Day
	7	=	Don't Know
	8	=	Refuse to Answer
	9	=	Not Applicable

RTU49.	TYPEA4. Which types of tobacco has [Response to RES35] ever smoked, even a puff? 
(mark all that apply)
TYPEA4A	TYPEA4. Which types of tobacco has [FNADLT4] ever smoked, even a puff? (mark all that apply): cigarette	1
	0	=	No
	1	=	Yes
	7	=	Don't Know
	8	=	Refuse to Answer
	9	=	Not Applicable

TYPEA4B	TYPEA4. Which types of tobacco has [FNADLT4] ever smoked, even a puff? (mark all that apply): cigar	1
	0	=	No
	1	=	Yes
	7	=	Don't Know
	8	=	Refuse to Answer
	9	=	Not Applicable

TYPEA4C	TYPEA4. Which types of tobacco has [FNADLT4] ever smoked, even a puff? (mark all that apply): pipe	1
	0	=	No
	1	=	Yes
	7	=	Don't Know
	8	=	Refuse to Answer
	9	=	Not Applicable

TYPEA4D	TYPEA4. Which types of tobacco has [FNADLT4] ever smoked, even a puff? (mark all that apply): waterpipe/hookah	1
	0	=	No
	1	=	Yes
	7	=	Don't Know
	8	=	Refuse to Answer
	9	=	Not Applicable

TYPEA4E	TYPEA4. Which types of tobacco has [FNADLT4] ever smoked, even a puff? (mark all that apply): other (specify)	1
	0	=	No
	1	=	Yes
	7	=	Don't Know
	8	=	Refuse to Answer
	9	=	Not Applicable

TYPEA4F	TYPEA4. Which types of tobacco has [FNADLT4] ever smoked, even a puff? (mark all that apply): none	1
	0	=	No
	1	=	Yes
	7	=	Don't Know
	8	=	Refuse to Answer
	9	=	Not Applicable


RTU50.	TYPEA4S. Please specify other type of tobacco smoked ever
TYPEA4S	TYPEA4S. Please specify other type of tobacco smoked ever	20

RTU51.	TYP30A4. During the past 30 days which of the following types of tobacco has [Response to RES35] smoked? 
(mark all that apply)
TYP30A4A	TYP30A4. During the past 30 days which of the following types of tobacco has [FNADLT4] smoked?: cigarette	1
	0	=	No
	1	=	Yes
	7	=	Don't Know
	8	=	Refuse to Answer
	9	=	Not Applicable

TYP30A4B	TYP30A4. During the past 30 days which of the following types of tobacco has [FNADLT4] smoked?: cigar	1
	0	=	No
	1	=	Yes
	7	=	Don't Know
	8	=	Refuse to Answer
	9	=	Not Applicable

TYP30A4C	TYP30A4. During the past 30 days which of the following types of tobacco has [FNADLT4] smoked?: pipe	1
	0	=	No
	1	=	Yes
	7	=	Don't Know
	8	=	Refuse to Answer
	9	=	Not Applicable

TYP30A4D	TYP30A4. During the past 30 days which of the following types of tobacco has [FNADLT4] smoked?: waterpipe/hookah	1
	0	=	No
	1	=	Yes
	7	=	Don't Know
	8	=	Refuse to Answer
	9	=	Not Applicable

TYP30A4E	TYP30A4. During the past 30 days which of the following types of tobacco has [FNADLT4] smoked?: other (specify)	1
	0	=	No
	1	=	Yes
	7	=	Don't Know
	8	=	Refuse to Answer
	9	=	Not Applicable

TYP30A4F	TYP30A4. During the past 30 days which of the following types of tobacco has [FNADLT4] smoked?: none	1
	0	=	No
	1	=	Yes
	7	=	Don't Know
	8	=	Refuse to Answer
	9	=	Not Applicable


RTU52.	TYP30A4S. Please specify other type of tobacco smoked during the past 30 days
TYP30A4S	TYP30A4S. Please specify other type of tobacco smoked during the past 30 days	20

RTU53.	LIFEA4. Has [Response to RES35] smoked more than 100 cigarettes in their lifetime?
LIFEA4	LIFEA4. Has [FNADLT4] smoked more than 100 cigarettes in their lifetime?	1
	0	=	No
	1	=	Yes
	7	=	Don't Know
	8	=	Refuse to Answer
	9	=	Not Applicable

RTU54.	SMKNOWA4. Does [Response to RES35] currently smoke cigarettes not at all, some days, or every day?
SMKNOWA4	SMKNOWA4. Does [FNADLT4] currently smoke cigarettes not at all, some days, or every day?	1
	0	=	Not at All
	1	=	Some Days
	2	=	Every Day
	7	=	Don't Know
	8	=	Refuse to Answer
	9	=	Not Applicable

RTU55.	TYPEA5. Which types of tobacco has [Response to RES38] ever smoked, even a puff? 
(mark all that apply)
TYPEA5A	TYPEA5. Which types of tobacco has [FNADLT5] ever smoked, even a puff? (mark all that apply): cigarette	1
	0	=	No
	1	=	Yes
	7	=	Don't Know
	8	=	Refuse to Answer
	9	=	Not Applicable

TYPEA5B	TYPEA5. Which types of tobacco has [FNADLT5] ever smoked, even a puff? (mark all that apply): cigar	1
	0	=	No
	1	=	Yes
	7	=	Don't Know
	8	=	Refuse to Answer
	9	=	Not Applicable

TYPEA5C	TYPEA5. Which types of tobacco has [FNADLT5] ever smoked, even a puff? (mark all that apply): pipe	1
	0	=	No
	1	=	Yes
	7	=	Don't Know
	8	=	Refuse to Answer
	9	=	Not Applicable

TYPEA5D	TYPEA5. Which types of tobacco has [FNADLT5] ever smoked, even a puff? (mark all that apply): waterpipe/hookah	1
	0	=	No
	1	=	Yes
	7	=	Don't Know
	8	=	Refuse to Answer
	9	=	Not Applicable

TYPEA5E	TYPEA5. Which types of tobacco has [FNADLT5] ever smoked, even a puff? (mark all that apply): other (specify)	1
	0	=	No
	1	=	Yes
	7	=	Don't Know
	8	=	Refuse to Answer
	9	=	Not Applicable

TYPEA5F	TYPEA5. Which types of tobacco has [FNADLT5] ever smoked, even a puff? (mark all that apply): none	1
	0	=	No
	1	=	Yes
	7	=	Don't Know
	8	=	Refuse to Answer
	9	=	Not Applicable


RTU56.	TYPEA5S. Please specify other type of tobacco smoked ever
TYPEA5S	TYPEA5S. Please specify other type of tobacco smoked ever	20

RTU57.	TYP30A5. During the past 30 days which of the following types of tobacco has [Response to RES38] smoked? 
(mark all that apply)
TYP30A5A	TYP30A5. During the past 30 days which of the following types of tobacco has [FNADLT5] smoked?: cigarette	1
	0	=	No
	1	=	Yes
	7	=	Don't Know
	8	=	Refuse to Answer
	9	=	Not Applicable

TYP30A5B	TYP30A5. During the past 30 days which of the following types of tobacco has [FNADLT5] smoked?: cigar	1
	0	=	No
	1	=	Yes
	7	=	Don't Know
	8	=	Refuse to Answer
	9	=	Not Applicable

TYP30A5C	TYP30A5. During the past 30 days which of the following types of tobacco has [FNADLT5] smoked?: pipe	1
	0	=	No
	1	=	Yes
	7	=	Don't Know
	8	=	Refuse to Answer
	9	=	Not Applicable

TYP30A5D	TYP30A5. During the past 30 days which of the following types of tobacco has [FNADLT5] smoked?: waterpipe/hookah	1
	0	=	No
	1	=	Yes
	7	=	Don't Know
	8	=	Refuse to Answer
	9	=	Not Applicable

TYP30A5E	TYP30A5. During the past 30 days which of the following types of tobacco has [FNADLT5] smoked?: other (specify)	1
	0	=	No
	1	=	Yes
	7	=	Don't Know
	8	=	Refuse to Answer
	9	=	Not Applicable

TYP30A5F	TYP30A5. During the past 30 days which of the following types of tobacco has [FNADLT5] smoked?: none	1
	0	=	No
	1	=	Yes
	7	=	Don't Know
	8	=	Refuse to Answer
	9	=	Not Applicable


RTU58.	TYP30A5S. Please specify other type of tobacco smoked during the past 30 days
TYP30A5S	TYP30A5S. Please specify other type of tobacco smoked during the past 30 days	20

RTU59.	LIFEA5. Has [Response to RES38] smoked more than 100 cigarettes in their lifetime?
LIFEA5	LIFEA5. Has [FNADLT5] smoked more than 100 cigarettes in their lifetime?	1
	0	=	No
	1	=	Yes
	7	=	Don't Know
	8	=	Refuse to Answer
	9	=	Not Applicable

RTU60.	SMKNOWA5. Does [Response to RES38] currently smoke cigarettes not at all, some days, or every day?
SMKNOWA5	SMKNOWA5. Does [FNADLT5] currently smoke cigarettes not at all, some days, or every day?	1
	0	=	Not at All
	1	=	Some Days
	2	=	Every Day
	7	=	Don't Know
	8	=	Refuse to Answer
	9	=	Not Applicable

RTU61.	SHARED. Is there a tobacco smoker living on the other side of a shared wall, or above or below your home? (check all that apply)
INTERVIEWER: If none apply, click "Next Question".
SHAREDA	SHARED. Is there a tobacco smoker living on the other side of a shared wall, or above or below your home? (check all that apply): other side of wall	1
	0	=	No
	1	=	Yes
	7	=	Don't Know
	8	=	Refuse to Answer
	9	=	Not Applicable

SHAREDB	SHARED. Is there a tobacco smoker living on the other side of a shared wall, or above or below your home? (check all that apply): above	1
	0	=	No
	1	=	Yes
	7	=	Don't Know
	8	=	Refuse to Answer
	9	=	Not Applicable

SHAREDC	SHARED. Is there a tobacco smoker living on the other side of a shared wall, or above or below your home? (check all that apply): below	1
	0	=	No
	1	=	Yes
	7	=	Don't Know
	8	=	Refuse to Answer
	9	=	Not Applicable


RTU62.	JUMPBK7
Interviewer: Do you need to jump to a previous section?
If so, click YES to return to the start of the interview, where you can then choose to jump ahead to any section.
Click NO to continue.
JUMPBK7	JUMPBK7. Jump back To a previous section?	1
	0	=	No
	1	=	Yes
	7	=	Don't Know
	8	=	Refuse to Answer
	9	=	Not Applicable

HTU1.	CIG7. How often in the past 7 days did anyone smoke cigarettes in your home?
CIG7	CIG7. How often in the past 7 days did anyone smoke cigarettes  in your home?	1
	0	=	never
	1	=	1 to 3 times
	2	=	4 to 6 times
	3	=	7 to 9 times
	4	=	10 or more times
	7	=	Don't Know
	8	=	Refuse to Answer
	9	=	Not Applicable

HTU2.	CIGAR7. How often in the past 7 days did anyone smoke cigars in your home?
CIGAR7	CIGAR7. How often in the past 7 days did anyone smoke cigars in your home?	1
	0	=	never
	1	=	1 to 3 times
	2	=	4 to 6 times
	3	=	7 to 9 times
	4	=	10 or more times
	7	=	Don't Know
	8	=	Refuse to Answer
	9	=	Not Applicable

HTU3.	PIPE7. How often in the past 7 days did anyone smoke pipe tobacco in your home?
PIPE7	PIPE7. How often in the past 7 days did anyone smoke pipe tobacco in your home?	1
	0	=	never
	1	=	1 to 3 times
	2	=	4 to 6 times
	3	=	7 to 9 times
	4	=	10 or more times
	7	=	Don't Know
	8	=	Refuse to Answer
	9	=	Not Applicable

HTU4.	HOOKAH7. How often in the past 7 days did anyone smoke hookah/waterpipe in your home?
HOOKAH7	HOOKAH7. How often in the past 7 days did anyone smoke hookah/waterpipe in your home?	1
	0	=	never
	1	=	1 to 3 times
	2	=	4 to 6 times
	3	=	7 to 9 times
	4	=	10 or more times
	7	=	Don't Know
	8	=	Refuse to Answer
	9	=	Not Applicable

HTU5.	ECIG7. How often in the past 7 days did anyone smoke electronic cigarettes in your home?
ECIG7	ECIG7. How often in the past 7 days did anyone smoke electronic cigarettes in your home?	1
	0	=	never
	1	=	1 to 3 times
	2	=	4 to 6 times
	3	=	7 to 9 times
	4	=	10 or more times
	7	=	Don't Know
	8	=	Refuse to Answer
	9	=	Not Applicable

HTU6.	MJ7. How often in the past 7 days did anyone smoke medicinal or recreational marijuana in your home?
MJ7	MJ7. How often in the past 7 days did anyone smoke medicinal or recreational marijuana in your home?	1
	0	=	never
	1	=	1 to 3 times
	2	=	4 to 6 times
	3	=	7 to 9 times
	4	=	10 or more times
	7	=	Don't Know
	8	=	Refuse to Answer
	9	=	Not Applicable

HTU7.	DRUGS7. How often in the past 7 days did anyone smoke other recreational drugs in your home?
DRUGS7	DRUGS7. How often in the past 7 days did anyone smoke other recreational drugs in your home?	1
	0	=	never
	1	=	1 to 3 times
	2	=	4 to 6 times
	3	=	7 to 9 times
	4	=	10 or more times
	7	=	Don't Know
	8	=	Refuse to Answer
	9	=	Not Applicable

HTU8.	COFFEE7. How often in the past 7 days did anyone drink coffee while smoking in your home?
COFFEE7	COFFEE7. How often in the past 7 days did anyone drink coffee while smoking in your home?	1
	0	=	never
	1	=	1 to 3 times
	2	=	4 to 6 times
	3	=	7 to 9 times
	4	=	10 or more times
	7	=	Don't Know
	8	=	Refuse to Answer
	9	=	Not Applicable

HTU9.	ALCOHOL7. How often in the past 7 days did anyone drink alcohol while smoking in your home?
ALCOHOL7	ALCOHOL7. How often in the past 7 days did anyone drink alcohol while smoking in your home?	1
	0	=	never
	1	=	1 to 3 times
	2	=	4 to 6 times
	3	=	7 to 9 times
	4	=	10 or more times
	7	=	Don't Know
	8	=	Refuse to Answer
	9	=	Not Applicable

SSE1.	CIGEXP. In the past 7 days, was [Response to RES1] exposed to any cigarettes in your home, a car, or any other place?
INTERVIEWER: "Other places" include garage, patio, front yard, back yard, park, etc.
If none apply, click "Next Question".
CIGEXP	CIGEXP. In the past 7 days, was [TC] exposed to any cigarettes in your home, a car, or any other place?	1
	0	=	No
	1	=	Yes
	7	=	Don't Know
	8	=	Refuse to Answer
	9	=	Not Applicable

SSE2.	CIGLOC. [IF YES cigarettes]  
Where? (check all that apply)
CIGLOCA	CIGLOC. [IF YES cigarettes]  Where? (check all that apply): your home	1
	0	=	No
	1	=	Yes
	7	=	Don't Know
	8	=	Refuse to Answer
	9	=	Not Applicable

CIGLOCB	CIGLOC. [IF YES cigarettes]  Where? (check all that apply): a car	1
	0	=	No
	1	=	Yes
	7	=	Don't Know
	8	=	Refuse to Answer
	9	=	Not Applicable

CIGLOCC	CIGLOC. [IF YES cigarettes]  Where? (check all that apply): other place	1
	0	=	No
	1	=	Yes
	7	=	Don't Know
	8	=	Refuse to Answer
	9	=	Not Applicable


SSE3.	CIGNUM. [IF YES cigarettes]  
What was the average number of cigarettes per day that [Response to RES1] was exposed to in your home, a car, or any other place in the past 7 days?
CIGNUM	CIGNUM. [IF YES cigarettes]  What was the average number of cigarettes per day that [TC] was exposed to in your home, a car, or any other place in the past 7 days?	3
	0 - 996	=	range
	997	=	Don't Know
	998	=	Refuse to Answer
	999	=	Not Applicable

SSE4.	OTHREXP. In the past 7 days, was [Response to RES1] exposed to any other tobacco products in the home, a car, or any other place? (check all that apply)
INTERVIEWER: "Other places" include garage, patio, front yard, back yard, park, etc.
If none apply, click "Next Question".
OTHREXPA	OTHREXP. In the past 7 days, was [TC] exposed to any other tobacco products in the home, a car, or any other place? (check all that apply): cigar	1
	0	=	No
	1	=	Yes
	7	=	Don't Know
	8	=	Refuse to Answer
	9	=	Not Applicable

OTHREXPB	OTHREXP. In the past 7 days, was [TC] exposed to any other tobacco products in the home, a car, or any other place? (check all that apply): pipes	1
	0	=	No
	1	=	Yes
	7	=	Don't Know
	8	=	Refuse to Answer
	9	=	Not Applicable

OTHREXPC	OTHREXP. In the past 7 days, was [TC] exposed to any other tobacco products in the home, a car, or any other place? (check all that apply): waterpipe/hookah	1
	0	=	No
	1	=	Yes
	7	=	Don't Know
	8	=	Refuse to Answer
	9	=	Not Applicable


SSE5.	CIGARNUM. [If YES cigars] 
What was the average number of cigars per day that [Response to RES1] was exposed to in your home, a car, or any other place in the past 7 days?
CIGARNUM	CIGARNUM. [If YES cigars] What was the average number of cigars per day that [TC] was exposed to in your home, a car, or any other place in the past 7 days?	3
	0 - 996	=	range
	997	=	Don't Know
	998	=	Refuse to Answer
	999	=	Not Applicable

SSE6.	PIPENUM. [If YES pipes] 
What was the average number of pipes per day that [Response to RES1] was exposed to in your home, a car, or any other place in the past 7 days?
PIPENUM	PIPENUM. [If YES pipes] What was the average number of pipes per day that [TC] was exposed to in your home, a car, or any other place in the past 7 days?	3
	0 - 996	=	range
	997	=	Don't Know
	998	=	Refuse to Answer
	999	=	Not Applicable

SSE7.	HOOKNUM. [If YES waterpipe/hookah]
What was the average number of hookah heads per day that [Response to RES1] was exposed to in your home, a car, or any other place in the past 7 days?
HOOKNUM	HOOKNUM. [If YES waterpipe/hookah] What was the average number of hookah heads per day that [TC] was exposed to in your home, a car, or any other place in the past 7 days?	3
	0 - 996	=	range
	997	=	Don't Know
	998	=	Refuse to Answer
	999	=	Not Applicable

SSE8.	JUMPBK8
Interviewer: Do you need to jump to a previous section?
If so, click YES to return to the start of the interview, where you can then choose to jump ahead to any section.
Click NO to continue.
JUMPBK8	JUMPBK8. Jump back To a previous section?	1
	0	=	No
	1	=	Yes
	7	=	Don't Know
	8	=	Refuse to Answer
	9	=	Not Applicable

SR1.	HOMRULE. How is tobacco smoking handled in your home? (check all that apply)
HOMRULEA	HOMRULE. How is tobacco smoking handled in your home? (check all that apply): no one allowed to smoke in the house	1
	0	=	No
	1	=	Yes
	7	=	Don't Know
	8	=	Refuse to Answer
	9	=	Not Applicable

HOMRULEB	HOMRULE. How is tobacco smoking handled in your home? (check all that apply): certain people or special guests allowed to smoke	1
	0	=	No
	1	=	Yes
	7	=	Don't Know
	8	=	Refuse to Answer
	9	=	Not Applicable

HOMRULEC	HOMRULE. How is tobacco smoking handled in your home? (check all that apply): allow smoking in certain areas inside the home	1
	0	=	No
	1	=	Yes
	7	=	Don't Know
	8	=	Refuse to Answer
	9	=	Not Applicable

HOMRULED	HOMRULE. How is tobacco smoking handled in your home? (check all that apply): allow smoking anywhere inside the home	1
	0	=	No
	1	=	Yes
	7	=	Don't Know
	8	=	Refuse to Answer
	9	=	Not Applicable


SR2.	CERTAIN - If certain people or special guests are allowed to smoke tobacco, who are these people? (check all that apply)
INTERVIEWER: If none apply, click "Next Question".
CERTAINA	CERTAIN. Who are certain people or special guests who are allowed to smoke tobacco? (check all that apply): Mother, father, mother-in-law, or father-in-law	1
	0	=	No
	1	=	Yes
	7	=	Don't Know
	8	=	Refuse to Answer
	9	=	Not Applicable

CERTAINB	CERTAIN. Who are certain people or special guests who are allowed to smoke tobacco? (check all that apply): Siblings	1
	0	=	No
	1	=	Yes
	7	=	Don't Know
	8	=	Refuse to Answer
	9	=	Not Applicable

CERTAINC	CERTAIN. Who are certain people or special guests who are allowed to smoke tobacco? (check all that apply): Other close relatives	1
	0	=	No
	1	=	Yes
	7	=	Don't Know
	8	=	Refuse to Answer
	9	=	Not Applicable

CERTAIND	CERTAIN. Who are certain people or special guests who are allowed to smoke tobacco? (check all that apply): Friends of the family	1
	0	=	No
	1	=	Yes
	7	=	Don't Know
	8	=	Refuse to Answer
	9	=	Not Applicable

CERTAINE	CERTAIN. Who are certain people or special guests who are allowed to smoke tobacco? (check all that apply): Acquaintances	1
	0	=	No
	1	=	Yes
	7	=	Don't Know
	8	=	Refuse to Answer
	9	=	Not Applicable

CERTAINF	CERTAIN. Who are certain people or special guests who are allowed to smoke tobacco? (check all that apply): Other persons (specify)	1
	0	=	No
	1	=	Yes
	7	=	Don't Know
	8	=	Refuse to Answer
	9	=	Not Applicable


SR3.	CERTSP. Specify Other: certain people or special guests who are allowed to smoke
CERTSP	CERTSP. Specify Other: certain people or special guests who are allowed to smoke	20

SR4.	LONG. About how long have you had these rules about tobacco smoking in your home?
Interviewer: Select unit of time on the next screen.
LONG	LONG. About how long have you had these rules about tobacco smoking in your home?	3
	0 - 996	=	range
	997	=	Don't Know
	998	=	Refuse to Answer
	999	=	Not Applicable

SR5.	LONGUT. Interviewer: Select unit of time that the participant has had these rules about tobacco smoking in the home
LONGUT	LONGUT. Unit of time that the participant has had these rules about tobacco smoking in the home	1
	1	=	days
	2	=	weeks
	3	=	months
	4	=	years
	7	=	Don't Know
	8	=	Refuse to Answer
	9	=	Not Applicable

SR6.	BROKEN. About how often are the rules about tobacco smoking in your home broken?
BROKEN	BROKEN. About how often are the rules about tobacco smoking in your home broken?	2
	0	=	Never
	1	=	less than once a year
	2	=	once a year
	3	=	once every several months
	4	=	once a month
	5	=	once a week
	6	=	daily
	7	=	more than once a day
	97	=	Don't Know
	98	=	Refuse to Answer
	99	=	Not Applicable

SR7.	ENFORCE. How are the rules about tobacco smoking in your home enforced? (check all that apply)
INTERVIEWER: If none apply, click "Next Question".
ENFORCEA	ENFORCE. How are the rules about tobacco smoking in your home enforced? (check all that apply): Telling people not to smoke in the home	1
	0	=	No
	1	=	Yes
	7	=	Don't Know
	8	=	Refuse to Answer
	9	=	Not Applicable

ENFORCEB	ENFORCE. How are the rules about tobacco smoking in your home enforced? (check all that apply): Keeping ashtrays out of the home	1
	0	=	No
	1	=	Yes
	7	=	Don't Know
	8	=	Refuse to Answer
	9	=	Not Applicable

ENFORCEC	ENFORCE. How are the rules about tobacco smoking in your home enforced? (check all that apply): Putting up No Smoking signs	1
	0	=	No
	1	=	Yes
	7	=	Don't Know
	8	=	Refuse to Answer
	9	=	Not Applicable

ENFORCED	ENFORCE. How are the rules about tobacco smoking in your home enforced? (check all that apply): Asking people to go outside when they want to smoke	1
	0	=	No
	1	=	Yes
	7	=	Don't Know
	8	=	Refuse to Answer
	9	=	Not Applicable

ENFORCEE	ENFORCE. How are the rules about tobacco smoking in your home enforced? (check all that apply): Other ways (specify)	1
	0	=	No
	1	=	Yes
	7	=	Don't Know
	8	=	Refuse to Answer
	9	=	Not Applicable


SR8.	ENFORCSP. Specify Other: how are rules in home enforced
ENFORCSP	ENFORCSP. Specify Other: how are rules in home enforced	20

SR9.	WHORULE. Who was mostly responsible for establishing the rules on smoking inside your current home?
WHORULE	WHORULE. Who was mostly responsible for establishing the rules on smoking inside your current home?	2
	1	=	The Participant
	2	=	Their Spouse/Partner
	3	=	Their Children
	4	=	Their Parents
	5	=	Their Siblings
	6	=	Their Roomate(s)
	7	=	Other Residents
	8	=	Other Nonresidents
	97	=	Don't Know
	98	=	Refuse to Answer
	99	=	Not Applicable

SR10.	SRRES_SP. Specify the resident who was mostly responsible for establishing the rules on smoking inside your current home.
SRRES_SP	SRRES_SP. Specify the resident who was mostly responsible for establishing the rules on smoking inside your current home.	20

SR11.	SRNON_SP. Specify the non-resident who was mostly responsible for establishing the rules on smoking inside your current home.
SRNON_SP	SRNON_SP. Specify the non-resident who was mostly responsible for establishing the rules on smoking inside your current home.	20

SR12.	SRHELP. How much do the household residents help to enforce the rules or customs regarding smoking?
SRHELP	SRHELP. How much do the household residents help to enforce the rules or customs regarding smoking?	1
	1	=	A lot
	2	=	Somewhat
	3	=	Not at all
	7	=	Don't Know
	8	=	Refuse to Answer
	9	=	Not Applicable

SR13.	SRQUIT. To what degree have the rules helped a household resident quit or reduce smoking? Have they helped…
SRQUIT	SRQUIT. To what degree have the rules helped a household resident quit or reduce smoking? Have they helped?	1
	1	=	A lot
	2	=	Somewhat
	3	=	Not at all
	7	=	Don't Know
	8	=	Refuse to Answer
	9	=	Not Applicable

SR14.	SRWHY. Did you create a home ban on tobacco smoking for any or all of the following reasons?
SRWHYA	SRWHY. Did you create a home ban on tobacco smoking for any or all of the following reasons?: to protect yourself from secondhand smoke	1
	0	=	No
	1	=	Yes
	97	=	Don't Know
	98	=	Refuse to Answer
	99	=	Not Applicable

SRWHYB	SRWHY. Did you create a home ban on tobacco smoking for any or all of the following reasons?: to protect your youngest child from secondhand smoke	1
	0	=	No
	1	=	Yes
	97	=	Don't Know
	98	=	Refuse to Answer
	99	=	Not Applicable

SRWHYC	SRWHY. Did you create a home ban on tobacco smoking for any or all of the following reasons?: to protect others from secondhand smoke	1
	0	=	No
	1	=	Yes
	97	=	Don't Know
	98	=	Refuse to Answer
	99	=	Not Applicable

SRWHYD	SRWHY. Did you create a home ban on tobacco smoking for any or all of the following reasons?: to encourage smoking residents to quit or reduce smoking	1
	0	=	No
	1	=	Yes
	97	=	Don't Know
	98	=	Refuse to Answer
	99	=	Not Applicable

SRWHYE	SRWHY. Did you create a home ban on tobacco smoking for any or all of the following reasons?: to avoid odors from tobacco smoking	1
	0	=	No
	1	=	Yes
	97	=	Don't Know
	98	=	Refuse to Answer
	99	=	Not Applicable

SRWHYF	SRWHY. Did you create a home ban on tobacco smoking for any or all of the following reasons?: to avoid being bothered by tobacco smoke	1
	0	=	No
	1	=	Yes
	97	=	Don't Know
	98	=	Refuse to Answer
	99	=	Not Applicable

SRWHYG	SRWHY. Did you create a home ban on tobacco smoking for any or all of the following reasons?: to avoid bothering others with tobacco smoke	1
	0	=	No
	1	=	Yes
	97	=	Don't Know
	98	=	Refuse to Answer
	99	=	Not Applicable

SRWHYH	SRWHY. Did you create a home ban on tobacco smoking for any or all of the following reasons?: Other (specify)	1
	0	=	No
	1	=	Yes
	97	=	Don't Know
	98	=	Refuse to Answer
	99	=	Not Applicable


SR15.	SRWHYSP. Specify Other: reason for creating a home ban on tobacco smoking
SRWHYSP	SRWHYSP. Specify Other: reason for creating a home ban on tobacco smoking	20

SR16.	INTENT. Which of the following best describes your intentions to set up a rule that bans smoking in your home? Would you say that you…
INTENT	INTENT. Which best describes your intentions to set up a rule that bans smoking in your home? Would you say that you	1
	1	=	Never expect to set up a rule that bans smoking in your home
	2	=	May set up a rule in the future, but not in the next 6 months
	3	=	Will set up a rule in the next 6 months
	4	=	Will set up a rule in the next month
	7	=	Don't Know
	8	=	Refuse to Answer
	9	=	Not Applicable

SR17.	CONSID. Would you consider creating a home ban on tobacco smoking  for any or all of the following reasons? (check all that apply)
CONSIDA	CONSID. Would you consider creating a home ban on tobacco smoking  for any or all of the following reasons?: to protect yourself from secondhand smoke	1
	0	=	No
	1	=	Yes
	97	=	Don't Know
	98	=	Refuse to Answer
	99	=	Not Applicable

CONSIDB	CONSID. Would you consider creating a home ban on tobacco smoking  for any or all of the following reasons?: to protect your youngest child from secondhand smoke	1
	0	=	No
	1	=	Yes
	97	=	Don't Know
	98	=	Refuse to Answer
	99	=	Not Applicable

CONSIDC	CONSID. Would you consider creating a home ban on tobacco smoking  for any or all of the following reasons?: to protect others from secondhand smoke	1
	0	=	No
	1	=	Yes
	97	=	Don't Know
	98	=	Refuse to Answer
	99	=	Not Applicable

CONSIDD	CONSID. Would you consider creating a home ban on tobacco smoking  for any or all of the following reasons?: to encourage smoking residents to quit or reduce smoking	1
	0	=	No
	1	=	Yes
	97	=	Don't Know
	98	=	Refuse to Answer
	99	=	Not Applicable

CONSIDE	CONSID. Would you consider creating a home ban on tobacco smoking  for any or all of the following reasons?: to avoid odors from tobacco smoking	1
	0	=	No
	1	=	Yes
	97	=	Don't Know
	98	=	Refuse to Answer
	99	=	Not Applicable

CONSIDF	CONSID. Would you consider creating a home ban on tobacco smoking  for any or all of the following reasons?: to avoid being bothered by tobacco smoke	1
	0	=	No
	1	=	Yes
	97	=	Don't Know
	98	=	Refuse to Answer
	99	=	Not Applicable

CONSIDG	CONSID. Would you consider creating a home ban on tobacco smoking  for any or all of the following reasons?: to avoid bothering others with tobacco smoke	1
	0	=	No
	1	=	Yes
	97	=	Don't Know
	98	=	Refuse to Answer
	99	=	Not Applicable

CONSIDH	CONSID. Would you consider creating a home ban on tobacco smoking  for any or all of the following reasons?: other (specify)	1
	0	=	No
	1	=	Yes
	97	=	Don't Know
	98	=	Refuse to Answer
	99	=	Not Applicable


SR18.	CONSIDSP. Specify the reason for creating a home ban on tobacco smoking
CONSIDSP	CONSIDSP. Specify the reason for creating a home ban on tobacco smoking	20

SR19.	HOMRUL2. How is smoking something other than tobacco (for example, marijuana) handled in your home? (check all that apply)
HOMRUL2A	HOMRUL2. How is smoking something other than tobacco (for example, marijuana) handled in your home?: no one allowed to smoke in the house	1
	0	=	No
	1	=	Yes
	7	=	Don't Know
	8	=	Refuse to Answer
	9	=	Not Applicable

HOMRUL2B	HOMRUL2. How is smoking something other than tobacco (for example, marijuana) handled in your home?: certain people or special guests allowed to smoke	1
	0	=	No
	1	=	Yes
	7	=	Don't Know
	8	=	Refuse to Answer
	9	=	Not Applicable

HOMRUL2C	HOMRUL2. How is smoking something other than tobacco (for example, marijuana) handled in your home?: allow smoking in certain areas inside the home	1
	0	=	No
	1	=	Yes
	7	=	Don't Know
	8	=	Refuse to Answer
	9	=	Not Applicable

HOMRUL2D	HOMRUL2. How is smoking something other than tobacco (for example, marijuana) handled in your home?: allow smoking anywhere inside the home	1
	0	=	No
	1	=	Yes
	7	=	Don't Know
	8	=	Refuse to Answer
	9	=	Not Applicable


SR20.	JUMPBK9
Interviewer: Do you need to jump to a previous section?
If so, click YES to return to the start of the interview, where you can then choose to jump ahead to any section.
Click NO to continue.
JUMPBK9	JUMPBK9. Jump back to a previous section?	1
	0	=	No
	1	=	Yes
	7	=	Don't Know
	8	=	Refuse to Answer
	9	=	Not Applicable

AV1.	AVBOTHER. I feel bothered when somebody smokes around me.
AVBOTHER	AVBOTHER. I feel bothered when somebody smokes around me.	1
	1	=	strongly agree
	2	=	somewhat agree
	3	=	do not agree at all
	7	=	Don't Know
	8	=	Refuse to Answer
	9	=	Not Applicable

AV2.	AVWORK. I prefer to work in smoke-free workplaces
AVWORK	AVWORK. I prefer to work in smoke-free workplaces	1
	1	=	strongly agree
	2	=	somewhat agree
	3	=	do not agree at all
	7	=	Don't Know
	8	=	Refuse to Answer
	9	=	Not Applicable

AV3.	AVPUBLIC. I prefer to be in smoke-free public places, such as restaurants, movie theaters, and public transportation
AVPUBLIC	AVPUBLIC. I prefer to be in smoke-free public places, such as restaurants, movie theaters, and public transportation	1
	1	=	strongly agree
	2	=	somewhat agree
	3	=	do not agree at all
	7	=	Don't Know
	8	=	Refuse to Answer
	9	=	Not Applicable

AV4A.	AVBAN. How much do you support or would you support a law banning smoking inside Workplaces? Would you support it a lot, somewhat, or not at all?
AVBANA	AVBAN. How much do you support or would you support a law banning smoking inside Workplaces? Would you support it a lot, somewhat, or not at all?	1
	1	=	a lot
	2	=	somewhat
	3	=	not at all
	7	=	Don't Know
	8	=	Refuse to Answer
	9	=	Not Applicable

AV4B.	AVBAN. How much do you support or would you support a law banning smoking inside Restaurants? Would you support it a lot, somewhat, or not at all?
AVBANB	AVBAN. How much do you support or would you support a law banning smoking inside Restaurants? Would you support it a lot, somewhat, or not at all?	1
	1	=	a lot
	2	=	somewhat
	3	=	not at all
	7	=	Don't Know
	8	=	Refuse to Answer
	9	=	Not Applicable

AV4C.	AVBAN. How much do you support or would you support a law banning smoking inside Public Transportation? Would you support it a lot, somewhat, or not at all?
AVBANC	AVBAN. How much do you support or would you support a law banning smoking inside Public Transportation? Would you support it a lot, somewhat, or not at all?	1
	1	=	a lot
	2	=	somewhat
	3	=	not at all
	7	=	Don't Know
	8	=	Refuse to Answer
	9	=	Not Applicable

AV4D.	AVBAN. How much do you support or would you support a law banning smoking inside Schools? Would you support it a lot, somewhat, or not at all?
AVBAND	AVBAN. How much do you support or would you support a law banning smoking inside Schools? Would you support it a lot, somewhat, or not at all?	1
	1	=	a lot
	2	=	somewhat
	3	=	not at all
	7	=	Don't Know
	8	=	Refuse to Answer
	9	=	Not Applicable

AV4E.	AVBAN. How much do you support or would you support a law banning smoking inside Health centers and hospitals? Would you support it a lot, somewhat, or not at all?
AVBANE	AVBAN. How much do you support or would you support a law banning smoking inside Health centers and hospitals? Would you support it a lot, somewhat, or not at all?	1
	1	=	a lot
	2	=	somewhat
	3	=	not at all
	7	=	Don't Know
	8	=	Refuse to Answer
	9	=	Not Applicable

AV5.	JUMPBK10
Interviewer: Do you need to jump to a previous section?
If so, click YES to return to the start of the interview, where you can then choose to jump ahead to any section.
Click NO to continue.
JUMPBK10	JUMPBK10. Jump back To a previous section?	1
	0	=	No
	1	=	Yes
	7	=	Don't Know
	8	=	Refuse to Answer
	9	=	Not Applicable

SI1.	FRIENDS. When you and [Response to RES1] visit the home of a friend who smokes, how often do you ask the friend not to smoke around [Response to RES1]?
FRIENDS	FRIENDS. When you and [TC] visit the home of a friend who smokes, how often do you ask the friend not to smoke around [TC]?	1
	0	=	Never
	1	=	Rarely
	2	=	Sometimes
	3	=	Usually
	4	=	Always
	5	=	Never, because no friends smoke around &[TC]
	7	=	Don't Know
	8	=	Refuse to Answer
	9	=	Not Applicable

SI2.	FAMILY. When you and [Response to RES1] visit the home of a family member who smokes, how often do you ask the family member not to smoke around [Response to RES1]?
FAMILY	FAMILY. When you and [TC] visit the home of a family member who smokes, how often do you ask the family member not to smoke around [TC]?	1
	0	=	Never
	1	=	Rarely
	2	=	Sometimes
	3	=	Usually
	4	=	Always
	5	=	Never, because no family members smoke around &[TC]
	7	=	Don't Know
	8	=	Refuse to Answer
	9	=	Not Applicable

SI3A.	ENCOUR. Do(es) partner encourage, discourage, or neither encourage nor discourage, smoking in your home?
ENCOURA	ENCOUR. Do(es) partner encourage, discourage, or neither encourage nor discourage, smoking in your home?	1
	1	=	encourage
	2	=	discourage
	3	=	neither encourage nor discourage
	7	=	Don't Know
	8	=	Refuse to Answer
	9	=	Not Applicable

SI3B.	ENCOUR. Do(es) parents encourage, discourage, or neither encourage nor discourage, smoking in your home?
ENCOURB	ENCOUR. Do(es) parents encourage, discourage, or neither encourage nor discourage, smoking in your home?	1
	1	=	encourage
	2	=	discourage
	3	=	neither encourage nor discourage
	7	=	Don't Know
	8	=	Refuse to Answer
	9	=	Not Applicable

SI3C.	ENCOUR. Do(es) siblings encourage, discourage, or neither encourage nor discourage, smoking in your home?
ENCOURC	ENCOUR. Do(es) siblings encourage, discourage, or neither encourage nor discourage, smoking in your home?	1
	1	=	encourage
	2	=	discourage
	3	=	neither encourage nor discourage
	7	=	Don't Know
	8	=	Refuse to Answer
	9	=	Not Applicable

SI3D.	ENCOUR. Do(es) friends encourage, discourage, or neither encourage nor discourage, smoking in your home?
ENCOURD	ENCOUR. Do(es) friends encourage, discourage, or neither encourage nor discourage, smoking in your home?	1
	1	=	encourage
	2	=	discourage
	3	=	neither encourage nor discourage
	7	=	Don't Know
	8	=	Refuse to Answer
	9	=	Not Applicable

SI3E.	ENCOUR. Do(es) children  encourage, discourage, or neither encourage nor discourage, smoking in your home?
ENCOURE	ENCOUR. Do(es) children  encourage, discourage, or neither encourage nor discourage, smoking in your home?	1
	1	=	encourage
	2	=	discourage
	3	=	neither encourage nor discourage
	7	=	Don't Know
	8	=	Refuse to Answer
	9	=	Not Applicable

SI3F.	ENCOUR. Do(es) grandparents encourage, discourage, or neither encourage nor discourage, smoking in your home?
ENCOURF	ENCOUR. Do(es) grandparents encourage, discourage, or neither encourage nor discourage, smoking in your home?	1
	1	=	encourage
	2	=	discourage
	3	=	neither encourage nor discourage
	7	=	Don't Know
	8	=	Refuse to Answer
	9	=	Not Applicable

SI3G.	ENCOUR. Do(es) aunts/uncles encourage, discourage, or neither encourage nor discourage, smoking in your home?
ENCOURG	ENCOUR. Do(es) aunts/uncles encourage, discourage, or neither encourage nor discourage, smoking in your home?	1
	1	=	encourage
	2	=	discourage
	3	=	neither encourage nor discourage
	7	=	Don't Know
	8	=	Refuse to Answer
	9	=	Not Applicable

SI3H.	ENCOUR. Do(es) co-workers encourage, discourage, or neither encourage nor discourage, smoking in your home?
ENCOURH	ENCOUR. Do(es) co-workers encourage, discourage, or neither encourage nor discourage, smoking in your home?	1
	1	=	encourage
	2	=	discourage
	3	=	neither encourage nor discourage
	7	=	Don't Know
	8	=	Refuse to Answer
	9	=	Not Applicable

SI3I.	ENCOUR. Do(es) healthcare providers encourage, discourage, or neither encourage nor discourage, smoking in your home?
ENCOURI	ENCOUR. Do(es) healthcare providers encourage, discourage, or neither encourage nor discourage, smoking in your home?	1
	1	=	encourage
	2	=	discourage
	3	=	neither encourage nor discourage
	7	=	Don't Know
	8	=	Refuse to Answer
	9	=	Not Applicable

SI3J.	ENCOUR. Do(es) anyone else? encourage, discourage, or neither encourage nor discourage, smoking in your home?
ENCOURJ	ENCOUR. Do(es) anyone else? encourage, discourage, or neither encourage nor discourage, smoking in your home?	1
	1	=	encourage
	2	=	discourage
	3	=	neither encourage nor discourage
	7	=	Don't Know
	8	=	Refuse to Answer
	9	=	Not Applicable

SI4.	ENCOURJS. Specify Other: Who else regularly encourages, discourages or neither encourages nor discourages smoking in your home?
ENCOURJS	ENCOURJS. Specify Other: Who else regularly encourages, discourages or neither encourages nor discourages smoking in your home?	20

SI5.	CRITICIZ. How often have you seen someone criticized for smoking in public?
CRITICIZ	CRITICIZ. How often have you seen someone criticized for smoking in public?	1
	0	=	not at all
	1	=	not very often
	2	=	often
	3	=	very often
	7	=	Don't Know
	8	=	Refuse to Answer
	9	=	Not Applicable

SI6.	PRAISE. How often have you seen someone praised for putting out a cigarette while smoking in public?
PRAISE	PRAISE. How often have you seen someone praised for putting out a cigarette while smoking in public?	1
	0	=	not at all
	1	=	not very often
	2	=	often
	3	=	very often
	7	=	Don't Know
	8	=	Refuse to Answer
	9	=	Not Applicable

SI7.	DISAPPRV. How often have you seen someone show signs of disapproval when they see others smoking in public?
DISAPPRV	DISAPPRV. How often have you seen someone show signs of disapproval when they see others smoking in public?	1
	0	=	not at all
	1	=	not very often
	2	=	often
	3	=	very often
	7	=	Don't Know
	8	=	Refuse to Answer
	9	=	Not Applicable

SI8.	PERCENT. What percentage of smokers in California do you think will be asked not to smoke around children in public?
PERCENT	PERCENT. What percentage of smokers in California do you think will be asked not to smoke around children in public?	3
	0 - 100	=	range
	997	=	Don't Know
	998	=	Refuse to Answer
	999	=	Not Applicable

SI9.	JUMPBK11
Interviewer: Do you need to jump to a previous section?
If so, click YES to return to the start of the interview, where you can then choose to jump ahead to any section.
Click NO to continue.
JUMPBK11	JUMPBK11. Jump back To a previous section?	1
	0	=	No
	1	=	Yes
	7	=	Don't Know
	8	=	Refuse to Answer
	9	=	Not Applicable

DMG1.	EDUC. What is the highest level of education that you completed?
EDUC	EDUC. What is the highest level of education that you completed?	2
	1	=	Never went to school
	2	=	Some elementary school
	3	=	Elementary school
	4	=	Junior high school
	5	=	High School
	6	=	Trade, Vocational Training
	7	=	College, no degree
	8	=	College degree
	9	=	Graduate studies or Doctoral
	10	=	Other (specify):_____________
	97	=	Don't Know
	98	=	Refuse to Answer
	99	=	Not Applicable

DMG2.	EDUCSP. Please specify the other type of education you received that is not listed.
EDUCSP	EDUCSP. Please specify the other type of education you received that is not listed.	20

DMG3.	EDUCYRS. How many years of education did you complete?
EDUCYRS	EDUCYRS. How many years of education did you complete?	4
	0 - 96	=	range
	97	=	Don't Know
	98	=	Refuse to Answer
	99	=	Not Applicable

DMG4.	COUNTRY. In what country were you born?
COUNTRY	COUNTRY. In what country were you born?	20

DMG5.	EMPLOY. What is your current employment status?
EMPLOY	EMPLOY. What is your current employment status?	1
	0	=	Not currently employed
	1	=	Part-time employed
	2	=	Full-time Employed
	3	=	Homemaker
	4	=	Retired
	7	=	Don't Know
	8	=	Refuse to Answer
	9	=	Not Applicable

DMG6.	WORKRULE. What are the rules regarding smoking INSIDE your workplace?
WORKRULE	WORKRULE. What are the rules regarding smoking INSIDE your workplace?	1
	1	=	No one is allowed to smoke inside the workplace
	2	=	People are allowed to smoke only in certain area inside the workplace
	3	=	Only special people, such as customers or visitors are allowed to smoke inside the workplace
	4	=	Everyone is allowed to smoke everywhere
	7	=	Don't Know
	8	=	Refuse to Answer
	9	=	Not Applicable

DMG7.	MARITAL. What is your current marital status?
MARITAL	MARITAL. What is your current marital status?	1
	1	=	Single/Never Married
	2	=	Married
	3	=	Not married but living with partner
	4	=	Divorced/Separated
	5	=	Widowed
	7	=	Don't Know
	8	=	Refuse to Answer
	9	=	Not Applicable

DMG8.	STUDENT. What is your current student status?
STUDENT	STUDENT. What is your current student status?	1
	0	=	Not currently enrolled as a student
	1	=	Part-time student
	2	=	Full-time student
	7	=	Don't Know
	8	=	Refuse to Answer
	9	=	Not Applicable

DMG9.	TPHISP. Do you consider yourself Hispanic or Latino? Hispanic or Latino? That is, a person of Spanish culture or origin, such as a Mexican, Central American, Puerto Rican, or Cuban?
TPHISP	TPHISP. Do you consider yourself Hispanic?	1
	0	=	No
	1	=	Yes
	7	=	Don't Know
	8	=	Refuse to Answer
	9	=	Not Applicable

DMG10.	TPRACE. Which of the following best describes your race? Select all that apply.
TPRACEB	TPRACE. Which of the following best describes your race? Select all that apply.: American Indian or Alaskan Native	1
	0	=	No
	1	=	Yes
	7	=	Don't Know
	8	=	Refuse to Answer
	9	=	Not Applicable

TPRACEC	TPRACE. Which of the following best describes your race? Select all that apply.: Asian	1
	0	=	No
	1	=	Yes
	7	=	Don't Know
	8	=	Refuse to Answer
	9	=	Not Applicable

TPRACED	TPRACE. Which of the following best describes your race? Select all that apply.: Native Hawaiian or Other Pacific Islander	1
	0	=	No
	1	=	Yes
	7	=	Don't Know
	8	=	Refuse to Answer
	9	=	Not Applicable

TPRACEE	TPRACE. Which of the following best describes your race? Select all that apply.: Black or African American	1
	0	=	No
	1	=	Yes
	7	=	Don't Know
	8	=	Refuse to Answer
	9	=	Not Applicable

TPRACEF	TPRACE. Which of the following best describes your race? Select all that apply.: White (Caucasian)	1
	0	=	No
	1	=	Yes
	7	=	Don't Know
	8	=	Refuse to Answer
	9	=	Not Applicable

TPRACEG	TPRACE. Which of the following best describes your race? Select all that apply.: Other (specify)	1
	0	=	No
	1	=	Yes
	7	=	Don't Know
	8	=	Refuse to Answer
	9	=	Not Applicable


DMG11.	TPRACESP. Please specify your racial identity as best as you can.
TPRACESP	TPRACESP. Please specify your racial identity as best as you can	20

DMG12.	TCHISP. Do you consider [Response to RES1] Hispanic or Latino? That is, a person of Spanish culture or origin, such as a Mexican, Central American, Puerto Rican, or Cuban?
TCHISP	TCHISP. Do you consider [TC] Hispanic or Latino?	1
	0	=	No
	1	=	Yes
	7	=	Don't Know
	8	=	Refuse to Answer
	9	=	Not Applicable

DMG13.	TCRACE. Which of the following best describe [Response to RES1]'s race? Select all that apply.
TCRACEB	TCRACE. Which of the following best describe [TC]‘s race? Select all that apply.: American Indian or Alaskan Native	1
	0	=	No
	1	=	Yes
	7	=	Don't Know
	8	=	Refuse to Answer
	9	=	Not Applicable

TCRACEC	TCRACE. Which of the following best describe [TC]‘s race? Select all that apply.: Asian	1
	0	=	No
	1	=	Yes
	7	=	Don't Know
	8	=	Refuse to Answer
	9	=	Not Applicable

TCRACED	TCRACE. Which of the following best describe [TC]‘s race? Select all that apply.: Native Hawaiian or Other Pacific Islander	1
	0	=	No
	1	=	Yes
	7	=	Don't Know
	8	=	Refuse to Answer
	9	=	Not Applicable

TCRACEE	TCRACE. Which of the following best describe [TC]‘s race? Select all that apply.: Black or African American	1
	0	=	No
	1	=	Yes
	7	=	Don't Know
	8	=	Refuse to Answer
	9	=	Not Applicable

TCRACEF	TCRACE. Which of the following best describe [TC]‘s race? Select all that apply.: White (Caucasian)	1
	0	=	No
	1	=	Yes
	7	=	Don't Know
	8	=	Refuse to Answer
	9	=	Not Applicable

TCRACEG	TCRACE. Which of the following best describe [TC]‘s race? Select all that apply.: Other (specify)	1
	0	=	No
	1	=	Yes
	7	=	Don't Know
	8	=	Refuse to Answer
	9	=	Not Applicable


DMG14.	TCRACESP. Please specify [Response to RES1]'s racial identify as best as you can..
TCRACESP	TCRACESP. Please specify [TC]'s racial identify as best as you can.	20

DMG15.	INCOME. What is your total combined household income before taxes in the past year? Please include income from all sources including wages, salaries, social security, retirement, benefits, and help from relatives or friends.

INTERVIEWER: Do not read choices aloud.

INCOME	INCOME. What is your total combined household income before taxes in 2011? Please include income from all sources including wages, salaries, social security, retirement, benefits, and help from relatives or friends.	2
	0	=	less than $10,000
	1	=	$10,000 - $19,999
	2	=	$20,000 - $29,999
	3	=	$30,000 - $39,999
	4	=	$40,000 - $49,999
	5	=	$50,000 - $59,999
	6	=	$60,000 - $69,999
	7	=	$70,000 - $79,999
	8	=	$80,000 - $89,999
	9	=	$90,000 - $99,999
	10	=	$100,000 or more
	97	=	Don't Know
	98	=	Refuse to Answer
	99	=	Not Applicable

DMG16.	JUMPBK12
Interviewer: Do you need to jump to a previous section?
If so, click YES to return to the start of the interview, where you can then choose to jump ahead to any section.
Click NO to continue.
JUMPBK12	JUMPBK12. Jump back To a previous section?	1
	0	=	No
	1	=	Yes
	7	=	Don't Know
	8	=	Refuse to Answer
	9	=	Not Applicable

Calculated Variable
ENDTIME	ENDTIME
 	ENDTIME = Current time

DMG17.	JUMPBK13
Interviewer: Do you need to jump to a previous section?
If so, click YES to return to the start of the interview, where you can then choose to jump ahead to any section.
Click NO to continue.
JUMPBK13	JUMPBK13. Jump back To a previous section?	1
	0	=	No
	1	=	Yes
	7	=	Don't Know
	8	=	Refuse to Answer
	9	=	Not Applicable

ADM1.	NDMOVE. Was the air nicotine dosimeter still in the same room in which it was installed, as shown on the Floor Plan?
NDMOVE	NDMOVE. Was the air nicotine dosimeter still in the same room in which it was installed, as shown on the Floor Plan?	1
	0	=	No
	1	=	Yes

ADM2.	MNTRMOVE. Was each air monitor still in the same room in which it was installed, as shown on the Floor Plan?
MNTRMOVE	MNTRMOVE. Was each air monitor still in the same room in which it was installed, as shown on the Floor Plan?	1
	0	=	No
	1	=	Yes

ADM3.	MNTRSP. If NO, which monitor was moved? (check all that apply)
INTERVIEWER: If none apply, click "Next Question".
MNTRSPA	MNTRSP. If NO, which monitor was moved? (check all that apply): Monitor 1	1
	0	=	No
	1	=	Yes

MNTRSPB	MNTRSP. If NO, which monitor was moved? (check all that apply): Monitor 2	1
	0	=	No
	1	=	Yes


ADM4.	LANGUAGE. What language was the interview conducted in?
LANGUAGE	LANGUAGE. What language was the interview conducted in?	1
	1	=	English
	2	=	Spanish
	3	=	Other (Specify)
	7	=	Don't Know
	8	=	Refuse to Answer
	9	=	Not Applicable

ADM4sp.	LANGSP. Please specify the language conducted for this interview.
LANGSP	LANGSP. Please specify the language conducted for this interview.	20

ADM4spa.	LANGPROX. If proxy was used, record relationship to respondent and age
LANGPROX	LANGPROX. If proxy was used, record relationship to respondent and age	20

ADM5.	CESSINFO. Did TP ask you for any information about smoking cessation methods or programs?
CESSINFO	CESSINFO. Did TP ask you for any information about smoking cessation methods or programs?	1
	0	=	No
	1	=	Yes

ADM6.	CESSCOMM. If YES, provide comments if possible:
CESSCOMM	CESSCOMM. If YES, provide comments if possible:	20

ADM7.	REFER. Did you refer a smoking adult to their health-care provider for information or assistance with smoking cessation?
REFER	REFER. Did you refer a smoking adult to their health-care provider for information or assistance with smoking cessation?	1
	0	=	No
	1	=	Yes
	7	=	Don't Know
	8	=	Refuse to Answer
	9	=	Not Applicable

ADM8.	QUALITY. Please describe anything that you think might have affected the quality of this interview (if any; e.g., other family member present during interview):
QUALITY	QUALITY. Please describe anything that you think might have affected the quality of this interview (if any; e.g., other family member present during interview):	20

ADM9.	COMMENTS. Other comments that you think are important to know about this interview:
COMMENTS	COMMENTS. Other comments that you think are important to know about this interview:	20

ADM10.	ADVERSE. Did you note any potential adverse events during this interview? 
IF YES, COMPLETE ADVERSE EVENTS REPORT FORM ! ! ! )  
ADVERSE	ADVERSE. Did you note any potential adverse events during this interview? (IF YES, COMPLETE ADVERSE	1
	0	=	No
	1	=	Yes

ADM11.	JUMPBK14
Interviewer: Do you need to jump to a previous section?
If so, click YES to return to the start of the interview, where you can then choose to jump ahead to any section.
Click NO to continue.
JUMPBK14	JUMPBK14. Jump back To a previous section?	1
	0	=	No
	1	=	Yes
	7	=	Don't Know
	8	=	Refuse to Answer
	9	=	Not Applicable
